# A Noncarbenoid Approach to Imidazolidines via ZnCl_2_‑Catalyzed Annulation of 4‑Alkoxycarbonyl-1,2-diaza-1,3-dienes with 1,3,5-Triazinanes

**DOI:** 10.1021/acs.joc.5c01387

**Published:** 2025-08-29

**Authors:** Vittorio Ciccone, Sara Caselli, Giacomo Mari, Fabio Mantellini, Gianfranco Favi

**Affiliations:** Department of Biomolecular Sciences, Section of Chemistry and Pharmaceutical Technologies, 19044 University of Urbino “Carlo Bo”, Via Ca’ Le Suore, 2, 61029 Urbino, Italy

## Abstract

An unprecedented ZnCl_2_-catalyzed formal [2 + 2 + 1] annulation of 1,2-diaza-1,3-dienes (DDs) with hexahydro-1,3,5-triazines (HTs) has been accomplished, which provides imidazolidine frameworks with quaternary carbon centers. Thus, a new opportunity bypassing the use of hazardous diazo reagents is made possible by a unique carbene-like reactivity (C1 synthon) of readily available and safe 4-alkoxycarbonyl-1,2-diaza-1,3-butadienes. Besides, this noncarbenoid transformation can be implemented into a two-step three-component approach by utilizing the aromatic amine, 1,2-diaza-1,3-dienes and 1,3,5-triazines to synthesize differently substituted 1,3-diaryl imidazolidines.

## Graphical abstract

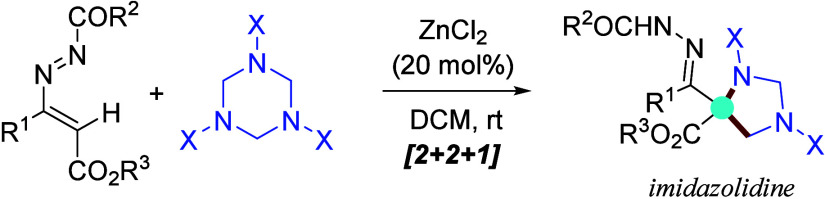

Imidazolidines are prevalent motifs in pharmaceutical, agrochemical, and (bio)­organic chemistry, such as chaetominine^1^ (strong cytotoxic against K562 human leukemia and SW1116 colon cancer cells), alchorneine^2^ with spasmolytic activity, cannabinoid CB2 receptor agonist, and chiral ligands/catalyst (MacMillan’s catalyst).^3^ This type of heterocycle is also useful as an organic synthon for various transformations.^4^

The application of bench-stable hexahydro-1,3,5-triazines (HTs)^5^ has had a profound impact recently, which is attributed to their different reaction modes. HTs have been employed not only in aminomethylation but also in various annulation reactions, where two, three, or four atoms (i.e., C–N, C–N–C, N–C–N, and C–N–C–N) can be incorporated into the final products. On the other hand, 1,2-diaza-1,3-dienes (DDs)^6^ have been recognized as versatile building blocks in organic synthesis, which could undergo a wide array of chemical transformations. Owing to their various possible reactivities as both heterodienes and electrophiles, they have frequently been employed for the assembly of various N-heterocyclic compounds.

Although much effort has been spent for the efficient constructions of imidazolidines,^7^ there is limited literature about building this framework bearing a quaternary stereocenter at the 4- or 5-position.^7c,8a,8c–8e^ Among the developed strategies, [4 + 1]-cycloadditions of diazocarbonyl precursors with hexahydro-1,3–5-triazines are the most straightforward and convenient methods.^8^ In particular, the gold-, copper- and iron-catalyzed cycloadditions have been reported by groups of Lu/Wang,^8b^ Sun,^8c,8d^ and others. Metal-free visible light and base-induced synthesis of imidazolidine scaffolds have also been described by groups of Wang/Xuan^8a^ and Sun^8d^ (Scheme 1a). However, the development of a reliable protocol that circumvents direct human exposure to toxic and explosive diazo reagents remains a significant challenge.

**Scheme 1:**
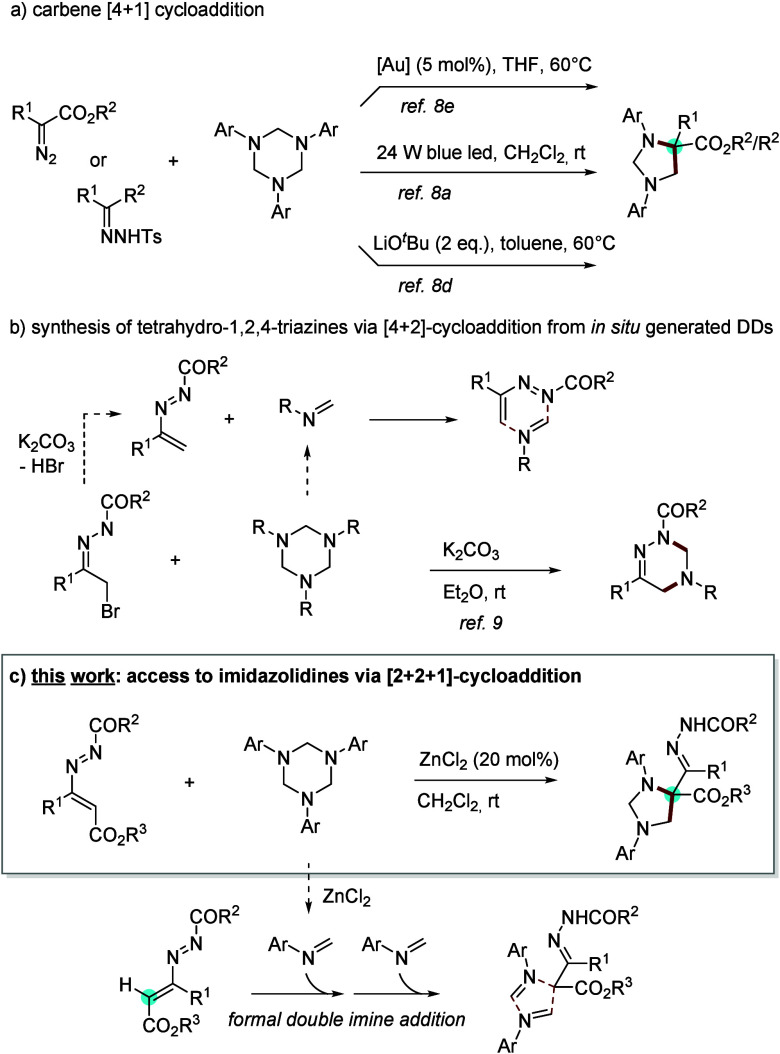
Previous Reports and Our Utilization of 1,3,5-Triazines

Recently, 1,2-diaza-1,3-dienes [azoalkenes], formed *in situ* from α-halo hydrazones in the presence of a base, have been employed as efficient dienes in inverse electron-demand aza-Diels–Alder (IEDDA) reaction with 1,3,5-triazinanes to synthesize tetrahydro-1,2,4-triazine derivatives (Scheme 1b).^9^ Inspired by these reports and as a continuation of our studies on the chemistry of DDs, we envisioned that a different reactivity of 4-alkoxycarbonyl-1,2-diaza-1,3-butadienes might occur upon the presence of a suitable catalyst. Herein, we report the unprecedented zinc-catalyzed [2 + 2 + 1] annulation between 1,2-diaza-1,3-dienes (DDs) and hexahydro-1,3,5-triazines (HTs) providing imidazolidine scaffolds (Scheme 1c). Notably, a rarely reported carbene-like C1 activity of 1,2-diaza-1,3-dienes is exhibited by the presence of an ester group (CO_2_R^3^) at the 4-position of the azoene system. Besides, different from Fang and Wang’s protocol^9^ in which the triazines acted as active imine intermediates undergoing [4 + 2] cycloadditions, we herein employ these as formal 1,4-dipoles under ZnCl_2_ catalysis to realize the synthesis of five-membered N-heterocycles.

At the outset, we selected 1,2-diaza-1,3-diene (**1a**) and commercially available 1,3,5-triphenyl-1,3,5-triazinane (**2a**) as the model substrates. When the reaction was performed in the presence of ZnCl_2_ (20 mol %) in DCM at room temperature by using a molar ratio of 1:1.3 between **1a** and **2a**, the imidazolidine **3a** was obtained in 33% yield (entry 1, Table 1). After a series of screenings (entries 1–6), the 2:1 molar ratio of **1a**:**2a** was found to be optimal delivering the desired product **3a** in nearly quantitative yield. Adding activated 4Å MS (entry 7) or decreasing the catalyst loading to 10 mol % (entry 8) was not beneficial. In the absence of Lewis acid, the reaction did not occur (entry 9). Other Lewis acid catalysts were also investigated, including ZnBr_2_, FeCl_2_, FeCl_3_, Cu­(OTf)_2_, Bi­(OTf)_3_, CuCl, CuCl_2_; however only inferior results were obtained (entries 10–16). A further solvent screen revealed that DMSO, acetonitrile, and THF could not improve the reaction efficiency (entries 17–19). Finally, no improvement was observed when the reaction was performed in DCE at 80 °C (entry 20).

**Table 1:**
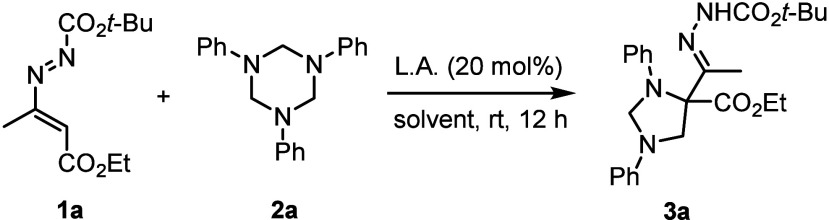
Optimization Studies^a^

entry	ratio **1a**:**2a**	catalyst	solvent	yield (%)^b^
1	1:1.3	ZnCl_2_	DCM	33
2	1:1	ZnCl_2_	DCM	67^c^
3	1.3:1	ZnCl_2_	DCM	72^c^
4	1.5:1	ZnCl_2_	DCM	82
5	1.8:1	ZnCl_2_	DCM	92
6	2:1	ZnCl_2_	DCM	96^c^
7^d^	2:1	ZnCl_2_	DCM	72
8^e^	2:1	ZnCl_2_	DCM	89
9	2:1	–	DCM	0
10	2:1	ZnBr_2_	DCM	84
11	2:1	FeCl_2_	DCM	0
12	2:1	FeCl_3_	DCM	0
13	2:1	Cu(OTf)_2_	DCM	24
14	2:1	Bi(OTf)_3_	DCM	15
15	2:1	CuCl	DCM	0
16	2:1	CuCl_2_	DCM	0
17	2:1	ZnCl_2_	DMSO	0
18	2:1	ZnCl_2_	CH_3_CN	39
19	2:1	ZnCl_2_	THF	77
20^f^	2:1	ZnCl_2_	DCE	86

a Reactions performed at 0.1 M solution of **2a** (0.15 mmol) unless otherwise noted.

b Determined by crude ^1^H NMR analysis using 2,5-dimethylfuran as the internal standard.

c Isolated yields.

d With activated 4 Å MS.

e 10 mol % of ZnCl_2_ was used.

f Performed at 80 °C.

With optimal conditions established, the scope of this [2 + 2 + 1] cycloaddition was explored. As shown in Scheme 2, a wide range of differently substituted azoalkenes (**1a**–**l**) (R^1^ = Me, *n*-Pr; R^2^ = OMe, OEt, *t*-Bu, OBn, NH_2_, NHPh; R^3^ = Me, Et, *t*-Bu, Bn, (CH_2_)_2_OMe) participated in the reaction to afford desired imidazolidines **3** in moderate to good yields (35–97%). Then a series of *N*-phenyl triazinanes (**2a**–**e**) bearing an electron-donating (methyl, methoxy), or electron-withdrawing (chloro), group were surveyed in the reaction. While *para*-methoxy and *para*-chloro/fluoro as substituents worked well, a significant effect on the yield was observed when using trimethylbenzyl-1,3,5-triazine (**3p**, 21%).

**Scheme 2:**
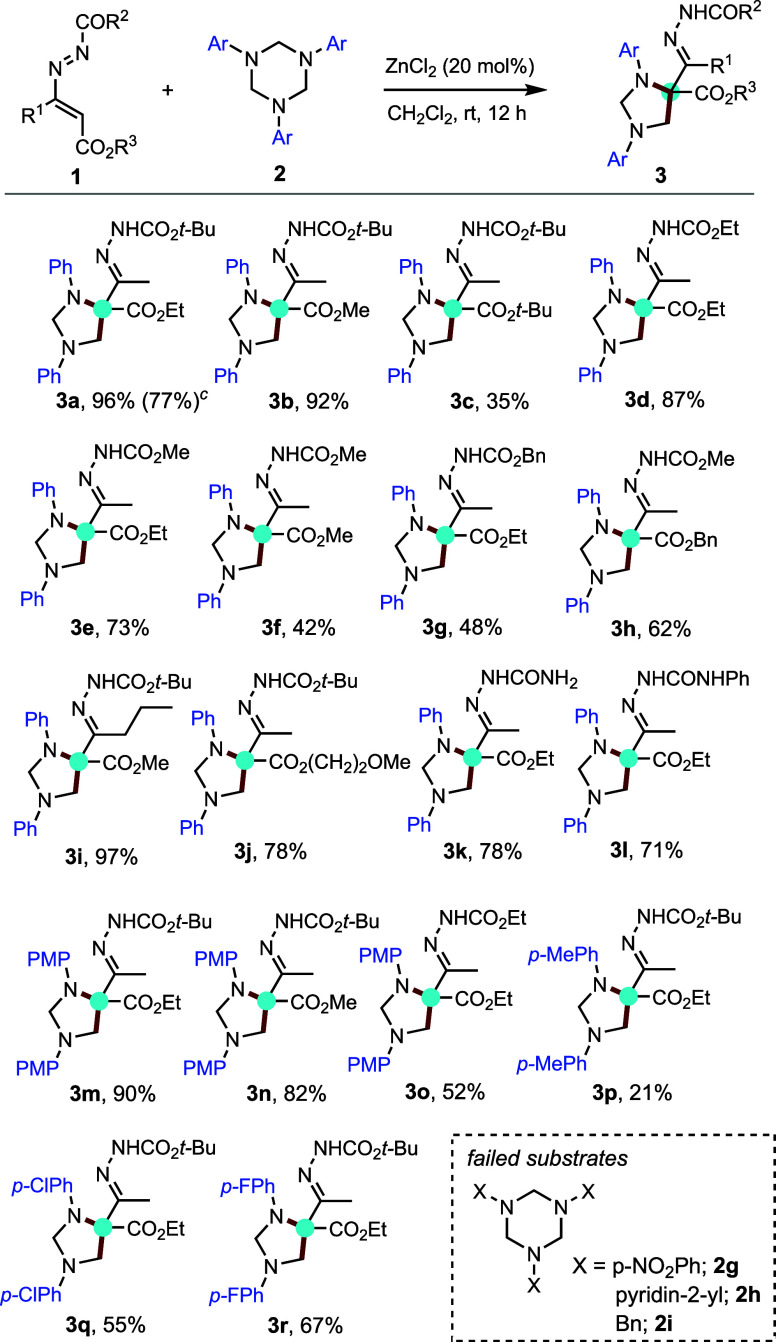
Synthesis of Imidazolidines **3** from 1,2-Diaza-1,3-dienes **1** and 1,3,5-Triaryl-1,3,5-triazinanes **2** ^a^ ^,^ ^b^

a Reactions performed with **1** (0.6 mmol), **2** (0.3 mmol) and ZnCl_2_ (20 mol %) in CH_2_Cl_2_ (3 mL) at rt for 12 h.
b Isolated yields.
c On a 1.0 mmol reaction scale. PMP = *p*-methoxyphenyl; Bn = benzyl.

Disappointingly, the use of 1,3,5-tri­(*p*-nitrophenyl)-1,3,5-triazinane (**2g**), 1,3,5-tri­(pyridin-2-yl)-1,3,5-triazinane (**2h**) and 1,3,5-tri­(benzyl)-1,3,5-triazinane (**2i**) delivered no identifiable imidazolidine products. For deactivated **2g** it was found that the reaction could only furnish a trace (5% yield) of Michael adduct **III** (*vide infra*) along with the recovery unreacted triazinane (92%) despite heating at 60 °C in DCE for 24 h.

The LCMS detection of α-aminohydrazone species **III** (*vide infra*) as s potential intermediate for the generation of cyclic imidazolidine **3a** prompted us to consider the assembly of differently substituted 1,3-diaryl imidazolidine compounds. To check our hypothesis, we carried out a sequential protocol where the α-aminohydrazone^10^ was preformed and employed to couple with 1,3,5-triazinanes. Attempts started with the ZnCl_2_-catalyzed 1,4-conjugate addition reaction between 1,2-diaza-1,3-diene (**1a**) and aniline (**A**) to form a Michael adduct (30 min, TLC monitoring), which was subsequently subjected to reaction with 1,3,5-triphenyl-1,3,5-triazinane **2b**. To our delight, desired imidazolidine **3Ab** was obtained in 84% yield. Thus, the possibility of reacting different α-aminohydrazone intermediates with various 1,3,5-triazinanes was explored to determine the scope of the tandem reaction to produce differently substituted 1,3-diaryl imidazolidines **3**. Various primary aromatic amine/*N*-aryltriazinane combinations worked well in this three-component telescopic reaction with DDs to provide the desired product **3** in good yields.

Interestingly, with this protocol, it is possible to reverse the aromatic substituents on the nitrogen atoms of imidazolidines by a judicious choice of the two *N*-components, namely amines **1** and triazinanes **2** (**3Ab** vs **3Ba**, **3Ac** vs **3Ca**, **3Ad** vs **3Da**, **3Ae** vs **3Ea**, **3Bc** vs **3Cb**, and **3Bd** vs **3Db**; Scheme 3).

**Scheme 3:**
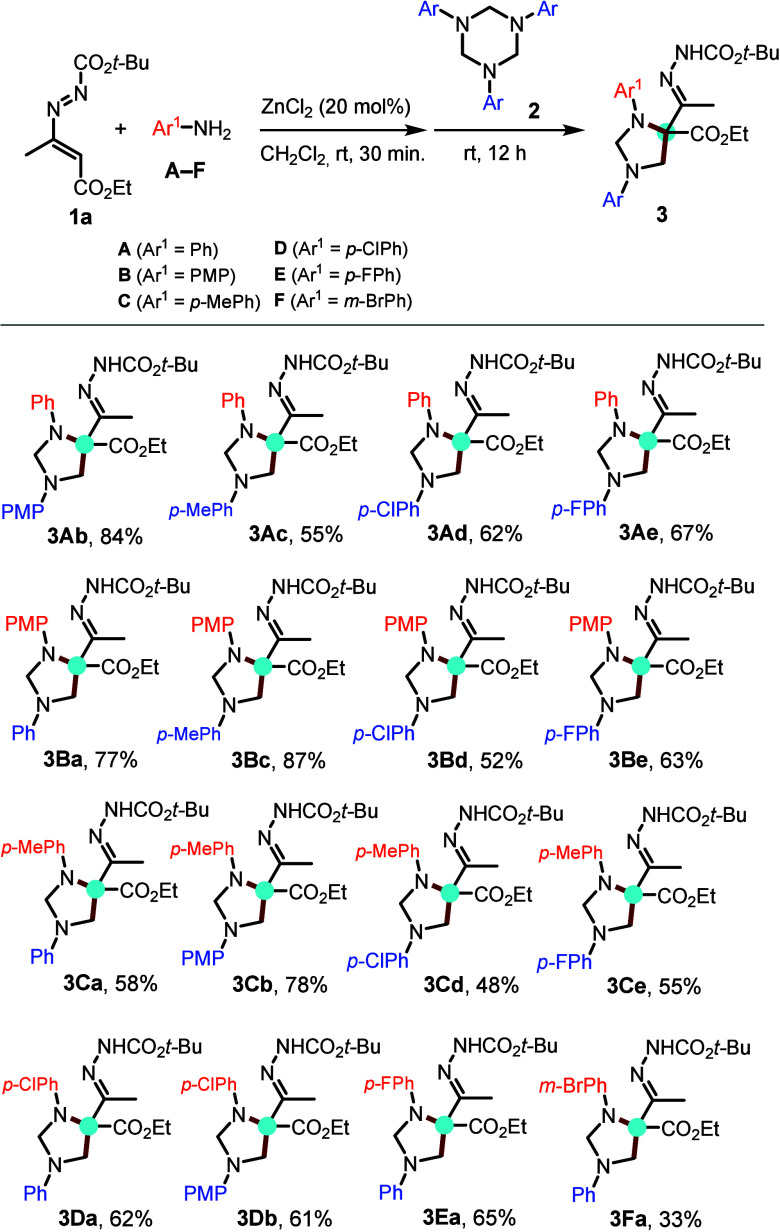
Synthesis of Differently Substituted 1,3-Diaryl Imidazolidines **3** from 1,2-Diaza-1,3-diene **1a**, Aromatic Amines **A**–**F** and 1,3,5-Triaryl-1,3,5-triazinanes **2** ^a^ ^,^ ^b^

a Reactions performed with **1** (0.3 mmol), **A–F** (0.3 mmol) and ZnCl_2_ (20 mol %) in CH_2_Cl_2_ (3 mL) at rt for 30 min.; then **2** (0.3 mmol) at rt for 12 h.
b Isolated yields.

On the basis of the results described above, a possible pathway for the assembly of imidazolidine is proposed (Scheme 4). Upon the ZnCl_2_ catalysis, it is hypothesized that N-aryl amine disassembling from 1,3,5-triazinane (ArNCH_2_)_3_ ^11^ would react with azoalkene **1** to provide an α-aminohydrazone intermediate **III**. The LCMS peak for **III** is consistent with the hypothesis that the aza-Michael addition step is likely to have a rate faster than those of other pathways. Subsequently, a formaldimine unit might react to give intermediate **IV**. Then, a further condensation of **IV** with a formaldehyde molecule followed by proto abstraction/intramolecular five-ring cyclization^12^ takes place to provide the final products **3**. Alternatively, the direct participation of imine monomer **I**,^10c,11,13^ the dimeric intermediate (1,4-dipole) **II**,^11d^ or triazine itself^8b,7e^ (the latter not shown) in the reaction with **1** to give the intermediate **V** should be excluded.

**Scheme 4:**
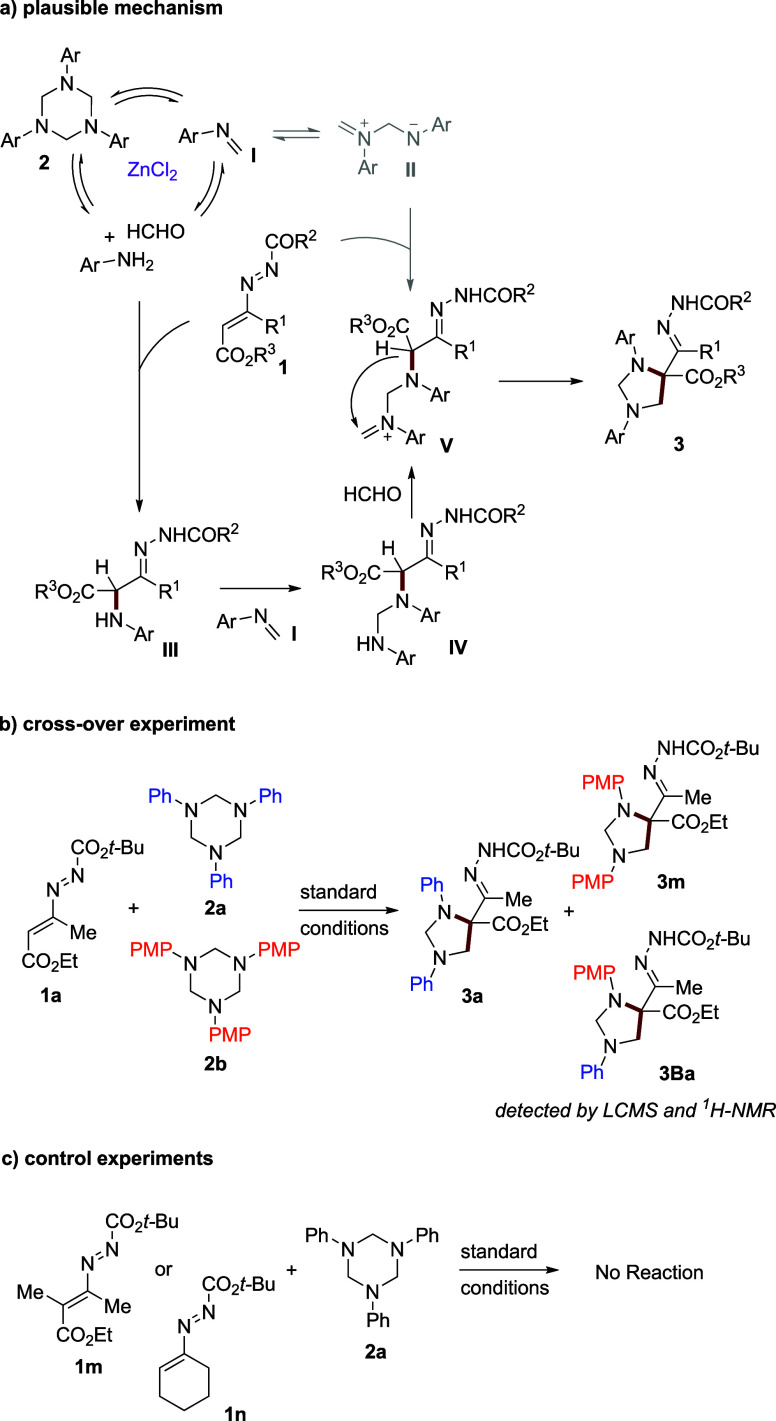
a) Possible Reaction Mechanism; b) Crossover Experiment; c) Control Experiments

A crossover experiment between DD **1a**, N-Ph triazinane **2a** and N-PMP triazinane **2b** provided a mixture of **3a**, **3m**, **3Ba**, which were confirmed by both LCMS and ^1^H NMR analysis (Scheme 4b). The formation of these inseparable cross cycloaddition products suggests that both 1,3,5-triazinanes **2a** and **2b** depolymerize under the reaction conditions into amine and formaldimine to participate in the transformation to final products. Our control experiments (Scheme 4c) also highlighted that both the terminal disubstituted azoalkene **1l** and the cyclic DD **1m** did not furnish the imidazolidine products under the standard conditions, indicating the need for an *acidic proton* in the C4 position of the starting DD.^9^

Hydrolytic cleavage of hydrazone product **3a** was performed to further demonstrate the utility of the present approach (Scheme 5). By treatment of **3a** with Amberlyst-15H and formaldehyde in acetone/H_2_O (9:1) solution^14^ the conversion of **3a** into the corresponding ketone **4a** proceeded in good yield.

**Scheme 5:**
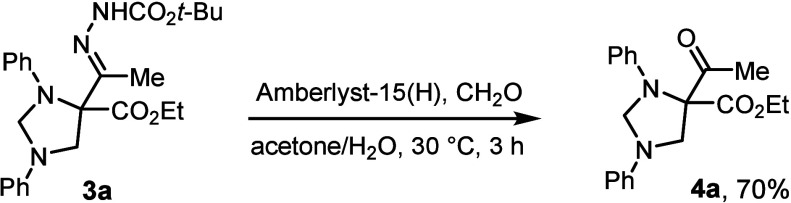


In conclusion, we have developed a formal [2 + 2 + 1] annulation of 1,2-diaza-1,3-dienes (DDs) with hexahydro-1,3,5-triazines (HTs) leading to 1,3-diaryl imidazolidines with quaternary carbon centers. Under the catalysis of a simple Lewis acid (ZnCl_2_) catalyst, 4-alkoxycarbonyl-DDs demonstrated unique carbene-like reactivity (C1 synthon), offering a safe alternative to the use of explosive/hazardous α-diazo compounds. The preliminary mechanistic studies indicate that the amine and formaldehyde (or formaldimine) derived in situ from depolymerization of the trimer (ArNCH_2_)_3_, instead of 1,3,5-triazine itself, are involved in the transformation. Meanwhile, this approach could be implemented in a three-component reaction by utilizing the aromatic amine, 1,2-diaza-1,3-dienes (DDs) and hexahydro-1,3,5-triazines (HTs) to reach differently substituted 1,3-diaryl imidazolidines. Further investigations based on this chemistry are currently underway.

## Supplementary Material





## Data Availability

The data underlying this study are available in the published article and its .

## Notes

Experimental procedures, characterization data, and NMR spectra of products (PDF)
FAIR data, including the primary NMR FID files, for compounds **3a**–**3r** and **3Ab**–**3Fa** (ZIP)
