# Supplementary material for: A Noncarbenoid Approach to Imidazolidines via ZnCl2‑Catalyzed Annulation of 4‑Alkoxycarbonyl-1,2-diaza-1,3-dienes with 1,3,5-Triazinanes
Source: J Org Chem. 2025 Aug 29;90(36):12820–5. doi: 10.1021/acs.joc.5c01387 (PMC12442072; doi:10.1021/acs.joc.5c01387)

Supporting Information

A Noncarbenoid Approach to Imidazolidines via $ZnCl_2$-Catalyzed Annulation of 4-Alkoxycarbonyl-1,2-diaza-1,3-dienes with 1,3,5-Triazinanes

*Vittorio Ciccone‡, Sara Caselli‡, Giacomo Mari, Fabio Mantellini, and Gianfranco Favi**

Department of Biomolecular Sciences, Section of Chemistry and Pharmaceutical Technologies, University of Urbino "Carlo Bo", Via Cà Le Suore, 2, 61029 Urbino, Italy

‡ These authors contributed equally to this work.

Email: gianfranco.favi@uniurb.it

Table of Contents

1. **General remarks** S2
2. **Substrates involved in the manuscript** S3
3. **Synthesis of 1,3,5-triazinanes 2** S4
4. **Synthesis and characterization of 1,3-diaryl imidazolidines 3a-r** S4–S14
   4.1 Procedures for the synthesis of **3a-r** S4
   4.2 Characterization of **3a-r** S4–S14
5. **Procedure for scale-up reaction** S14
6. **Synthesis and characterization of 1,3-diaryl imidazolidines 3Ab-Fa** S15–S24
   6.1 Procedures for the synthesis of **3Ab-Fa** S15
   6.2 Characterization of **3Ab-Fa** S15–S24
7. **Access to hydrolyzed imidazolidine 4a** S25
8. **Mechanistic investigation** S26–S27
9. **References** S27
10. **Copies of ^{1}H and ^{13}C NMR spectra** S28–S67

1. General Remarks

All the commercially available reagents and solvents were used without further purification. Amberlyst-15(H) DRY was purchased from Sigma-Aldrich. 1,3,5-Triphenyl-1,3,5-triazinane **2a**, amines **A–F**, and paraformaldehyde were commercial materials; 1,2-diaza-1,3-dienes (DDs) **1a–n**[1] and 1,3,5-triazinanes **2b–i**[2] were synthesized according to literature procedures. Chromatographic purification of compounds was carried out on silica gel (60–200 µm). TLC analysis was performed on pre-loaded (0.25 mm) glass supported silica gel plates (Kieselgel 60); compounds were visualized by exposure to UV light and by dipping the plates in 1% $Ce(SO_4)\cdot4H_2O$, 2.5% $(NH_4)_6Mo_7O_{24}\cdot4H_2O$ in 10% sulphuric acid followed by heating on a hot plate. All ^{1}H NMR, ^{13}C NMR and ^{19}F NMR spectra were recorded at 400, 101 and 376 MHz using DMSO-*d*$_6$ or $CDCl_3$ as solvent on a Bruker Ultrashield 400 spectrometer (Bruker, Billerica, MA, USA). Chemical shift (δ scale) are reported in parts per million (ppm) relative to the central peak of the solvent and are sorted in descending order within each group. The following abbreviations are used to describe peak patterns where appropriate: s = singlet, d = doublet, dd = doublet of doublet, dt = doublet of triplet, t = triplet, q = quartet, sept = septet, m = multiplet and bs = broad signal. All coupling constants (J value) are given in Hertz [Hz]. Structural assignments were made with additional information from gradient heteronuclear multiple quantum correlation (gHMQC), and gradient heteronuclear multiple bond correlation (gHMBC). High-resolution mass spectral (HRMS) analyses were performed using Orbitrap Exploris 240 Mass Spectrometers (Thermo Scientific) equipped with an ESI source. Melting points were determined in open capillary tubes and are uncorrected.

2. Substrates involved in the manuscript.

Azoalkenes

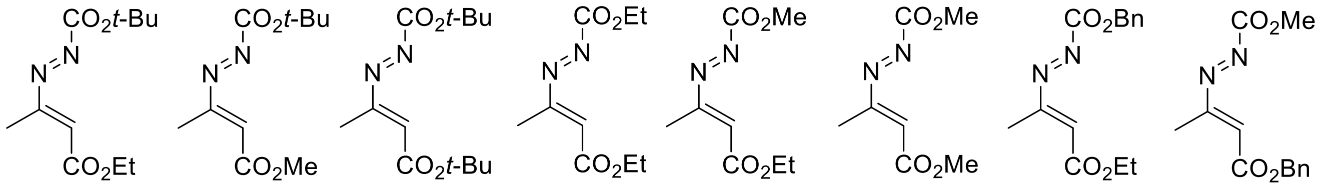

1a **1b** **1c** **1d** **1e** **1f** **1g** **1h**

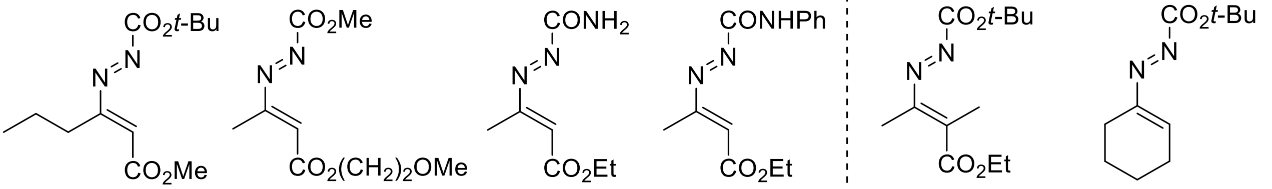

1i **1j** **1k** **1l** **1m** **1n**

failed azoalkenes

Triazinanes

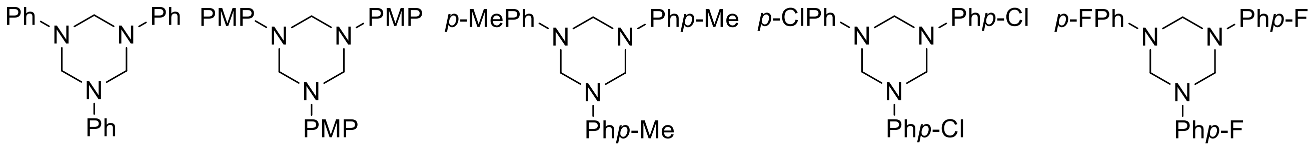

2a **2b** **2c** **2d** **2e**

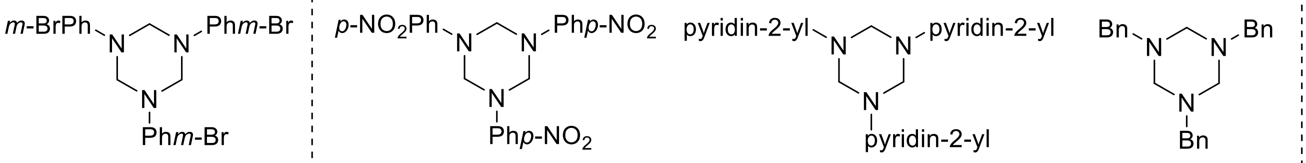

2f **2g** **2h** **2i**

failed triazinanes

Amines

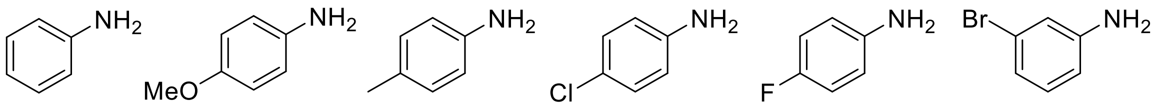

A **B** **C** **D** **E** **F**

3. Synthesis of 1,3,5-triazinanes 2.[2]

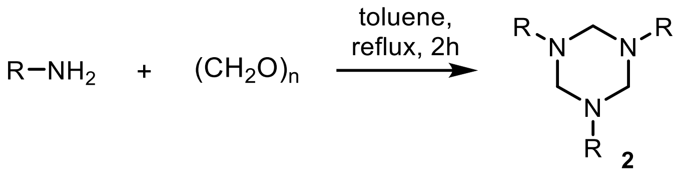

To a solution of amine (30 mmol) in anhydrous toluene (50 mL), paraformaldehyde (0.991 g, 33 mmol, 1.1 equiv) was added, and the mixture was stirred at reflux for 2 h. Then, the solvent was removed under reduced pressure, and a precipitate came out from the mixture. The precipitate was collected by filtration, washed with *n*-hexane several times, and dried to obtain 1,3,5-triazinane **2**.

4. Synthesis and characterization of 1,3-diaryl imidazolidines 3.

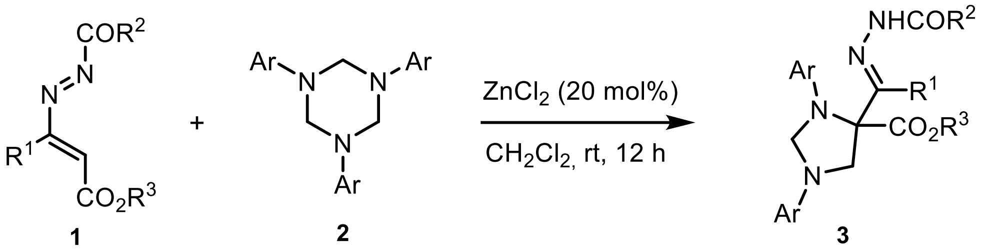

*4.1 Synthesis of imidazolidines **3** from 1,2-diaza-1,3-dienes **1** and 1,3,5-triaryl-1,3,5-triazinanes **2**.* To a solution of 1,2-diaza-1,3-diene **1** (0.6 mmol, 2 equiv) in CH_2Cl_2 (3 mL), 1,3,5-triaryl-1,3,5-triazinane **2** (0.3 mmol) and $ZnCl_2$ (8.2 mg, 0.06 mmol, 20 mol %) were added. After stirring for 12 h (TLC monitoring) at rt, the solvent was removed and the crude mixture directly purified through flash column chromatography on silica gel to afford the desired imidazolidine **3**.

*4.2 Characterization of imidazolidines **3a–r**:*

Ethyl-4-(1-(2-(*tert*-butoxycarbonyl)hydrazineylidene)ethyl)-1,3-diphenylimidazolidine-4-carboxylate (3a): compound **3a** was obtained by column chromatography (cycloehexane/ethyl acetate 8:2) in **96%** yield (130.2 mg), 12 h; white solid; mp: 181–183°C.

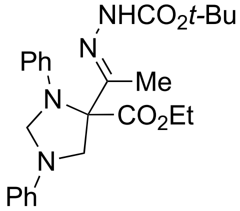

^{1}H NMR (400 MHz, DMSO-*d*$_6$) δ 9.83 (s, 1H), 7.26 (dd, *J* = 8.6, 7.3 Hz, 2H), 7.15 (dd, *J* = 8.6, 7.3 Hz, 2H), 6.90 – 6.87 (m, 5H), 6.72 (t, *J* = 7.3 Hz, 1H), 4.88 (d, *J* = 4.1 Hz, 1H), 4.66 (d, *J* = 4.1 Hz, 1H), 4.35 (d, *J* = 9.6 Hz, 1H), 4.20 – 4.03 (m, 2H), 3.42 (d, *J* = 9.6 Hz, 1H), 1.69 (s, 3H), 1.47 (s, 9H), 1.18 (t, *J* = 7.1 Hz, 3H).

^{13}C{^{1}H} NMR (101 MHz, DMSO-*d*$_6$) δ 170.7, 153.1, 149.6, 146.6, 144.6, 129.5, 128.8, 119.0, 117.9, 114.3, 114.1, 80.0, 75.8, 67.7, 61.4, 55.4, 28.5, 14.4, 13.6.

HRMS (ESI-Orbitrap, *m/z*): $[M+Na]^+$ Calcd for $C_{25}H_{32}N_4NaO_4$ 475.2321; Found 475.2337.

Methyl-4-(1-(2-(*tert*-butoxycarbonyl)hydrazineylidene)ethyl)-1,3-diphenylimidazolidine-4-carboxylate (3b): compound **3b** was obtained by column chromatography (cycloehexane/ethyl acetate 9:1) in **92%** yield (121.2 mg), 12 h; colorless oil.

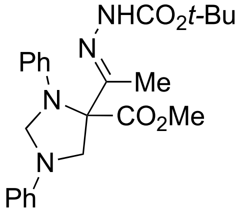

^{1}H NMR (400 MHz, CDCl$_3$) δ 7.90 (bs, 1H), 7.32 (t, *J* = 7.9 Hz, 2H), 7.23 (t, *J* = 8.4 Hz, 2H), 6.89 (t, *J* = 7.9 Hz, 1H), 6.85 – 6.80 (m, 3H), 6.73 (d, *J* = 8.4 Hz, 2H), 4.95 (d, *J* = 3.1 Hz, 1H), 4.81 (d, *J* = 3.1 Hz, 1H), 4.51 (d, *J* = 9.3 Hz, 1H), 3.81 (s, 3H), 3.46 (d, *J* = 9.3 Hz, 1H), 1.73 (s, 3H), 1.54 (s, 9H).

^{13}C{^{1}H} NMR (101 MHz, CDCl$_3$) δ 170.9, 147.8, 147.5, 146.0, 144.0, 129.4, 128.9, 119.1, 118.4, 113.7, 113.5, 81.7, 75.5, 67.4, 55.6, 52.7, 28.3, 11.7.

HRMS (ESI-Orbitrap, *m/z*): $[M+Na]^+$ Calcd for $C_{24}H_{30}N_4NaO_4$ 461.2165; Found 461.2149.

***tert*-Butyl-4-(1-(2-(*tert*-butoxycarbonyl)hydrazineylidene)ethyl)-1,3-diphenylimidazolidine-4-carboxylate (3c):** compound **3c** was obtained by column chromatography (cycloehexane/ethyl acetate 9:1) in **35%** yield (46.2 mg), 12 h; colorless oil.

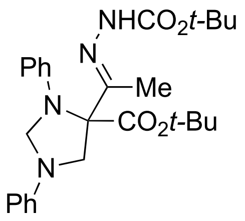

^{1}H NMR (400 MHz, DMSO-*d*$_6$) δ 9.72 (s, 1H), 7.31 – 7.23 (m, 2H), 7.18 – 7.12 (m, 2H), 6.87 – 6.76 (m, 5H), 6.71 (t, *J* = 7.2 Hz, 1H), 4.82 (d, *J* = 4.2 Hz, 1H), 4.69 (d, *J* = 4.2 Hz, 1H), 4.27 (d, *J* = 9.6 Hz, 1H), 3.40 (d, *J* = 9.6 Hz, 1H), 1.72 (s, 3H), 1.46 (s, 9H), 1.33 (s, 9H).

^{13}C{^{1}H} NMR (101 MHz, DMSO-*d*$_6$) δ 169.2, 153.2, 150.1, 146.7, 144.7, 129.5, 128.7, 118.7, 117.9, 114.3, 114.0, 81.7, 80.0, 75.9, 67.6, 55.5, 28.6, 28.2, 13.8.

HRMS (ESI-Orbitrap, *m/z*): $[M+Na]^+$ Calcd for $C_{27}H_{36}N_4NaO_4$ 503.2634; Found 503.2646.

Ethyl-4-(1-(2-(ethoxycarbonyl)hydrazineylidene)ethyl)-1,3-diphenylimidazolidine-4-carboxylate (3d): compound **3d** was obtained by column chromatography (cycloehexane/ethyl acetate 9:1) in **87%** yield (110.5 mg), 12 h; white solid; mp: 138–140 °C.

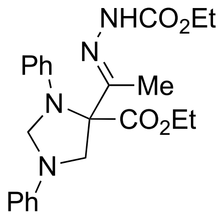

^{1}H NMR (400 MHz, CDCl$_3$) δ 7.86 (bs, 1H), 7.31 (t, *J* = 8.0 Hz, 2H), 7.25 – 7.18 (m, 2H), 6.88 (t, *J* = 8.0 Hz, 1H), 6.84 – 6.78 (m, 3H), 6.73 (d, *J* = 8.0 Hz, 2H), 4.93 (d, *J* = 3.2 Hz, 1H), 4.82 (d, *J* = 3.2 Hz, 1H), 4.48 (d, *J* = 9.4 Hz, 1H), 4.28 (q, *J* = 7.0 Hz, 4H), 3.49 (d, *J* = 9.4 Hz, 1H), 1.74 (s, 3H), 1.33 (t, *J* = 7.1 Hz, 3H), 1.29 (t, *J* = 7.1 Hz, 3H).

^{13}C{^{1}H} NMR (101 MHz, CDCl$_3$) δ 170.3, 153.8, 149.1, 146.1, 144.1, 129.5, 129.0, 119.1, 118.6, 113.9, 113.6, 75.5, 67.6, 62.3, 62.0, 55.8, 14.6, 14.3, 11.8.

HRMS (ESI-Orbitrap, *m/z*): $[M+Na]^+$ Calcd for $C_{23}H_{28}N_4NaO_4$ 447.2008; Found 447.2014.

Ethyl-4-(1-(2-(methoxycarbonyl)hydrazineylidene)ethyl)-1,3-diphenylimidazolidine-4-carboxylate (3e): compound **3e** was obtained by column chromatography (cycloehexane/ethyl acetate 9:1) in **73%** yield (89.4 mg), 12 h; white solid; mp: 145–147°C.

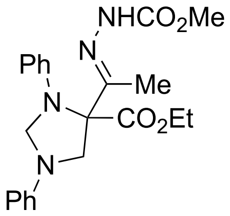

^{1}H NMR (400 MHz, CDCl$_3$) δ 8.15 (bs, 1H), 7.32 (t, *J* = 7.7 Hz, 2H), 7.22 (t, *J* = 7.7 Hz, 2H), 6.88 (t, *J* = 7.7 Hz, 1H), 6.84 – 6.79 (m, 3H), 6.73 (d, *J* = 7.7 Hz, 2H), 4.93 (d, *J* = 3.2 Hz, 1H), 4.83 (d, *J* = 3.2 Hz, 1H), 4.48 (d, *J* = 9.3 Hz, 1H), 4.34 – 4.23 (m, 2H), 3.82 (s, 3H), 3.49 (d, *J* = 9.3 Hz, 1H), 1.75 (s, 3H), 1.29 (t, *J* = 7.1 Hz, 3H).

^{13}C{^{1}H} NMR (101 MHz, CDCl$_3$) δ 170.2, 154.5, 149.4, 146.1, 144.1, 129.5, 128.9, 119.1, 118.6, 113.9, 113.5, 75.4, 67.5, 62.0, 55.7, 53.1, 14.1, 11.9.

HRMS (ESI-Orbitrap, *m/z*): $[M+Na]^+$ Calcd for $C_{22}H_{26}N_4NaO_4$ 433.1852; Found 433.1865.

Methyl-4-(1-(2-(methoxycarbonyl)hydrazineylidene)ethyl)-1,3-diphenylimidazolidine-4-carboxylate (3f): compound **3f** was obtained by column chromatography (cycloehexane/ethyl acetate 9:1) in **42%** yield (50.2 mg), 12 h; white solid; mp: 141–143 °C.

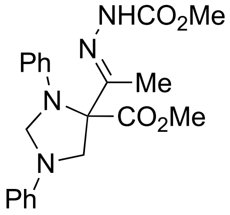

^{1}H NMR (400 MHz, CDCl$_3$) δ 8.06 (bs, 1H), 7.35 – 7.29 (m, 2H), 7.25 – 7.19 (m, 2H), 6.89 (t, *J* = 7.8 Hz, 1H), 6.85 – 6.77 (m, 3H), 6.73 (d, *J* = 7.8 Hz, 2H), 4.94 (d, *J* = 3.2 Hz, 1H), 4.82 (d, *J* = 3.2 Hz, 1H), 4.49 (d, *J* = 9.3 Hz, 1H), 3.83 (s, 3H), 3.81 (s, 3H), 3.48 (d, *J* = 9.3 Hz, 1H), 1.73 (s, 3H).

^{13}C{^{1}H} NMR (101 MHz, CDCl$_3$) δ 170.9, 154.4, 149.3, 146.0, 144.0, 129.5, 129.1, 119.2, 118.6, 113.7, 113.6, 75.6, 67.5, 55.7, 53.2, 52.8, 11.8.

HRMS (ESI-Orbitrap, *m/z*): $[M+Na]^+$ Calcd for $C_{21}H_{24}N_4NaO_4$ 419.1695; Found 419.1704.

Ethyl-4-(1-(2-(phenoxycarbonyl)hydrazineylidene)ethyl)-1,3-diphenylimidazolidine-4-carboxylate (3g): compound **3g** was obtained by column chromatography (cycloehexane/ethyl acetate 9:1) in **48%** yield (70.1 mg), 12 h; white solid; mp: 139–141 °C.

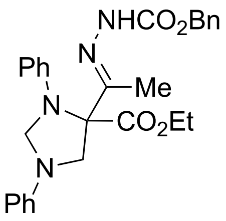

^{1}H NMR (400 MHz, CDCl$_3$) δ 8.16 (bs, 1H), 7.46 – 7.30 (m. 7H), 7.25 – 7.19 (m, 2H), 6.89 (t, *J* = 7.4 Hz, 1H), 6.86 – 6.76 (m, 3H), 6.73 (d, *J* = 8.5 Hz, 2H), 5.25 (s, 2H), 4.94 (d, *J* = 3.1 Hz, 1H), 4.83 (d, *J* = 3.1 Hz, 1H), 4.48 (d, *J* = 9.3 Hz, 1H), 4.27 – 4.09 (m, 2H), 3.49 (d, *J* = 9.3 Hz, 1H), 1.74 (s, 3H), 1.17 (bs, 3H).

^{13}C{^{1}H} NMR (101 MHz, CDCl$_3$) δ 170.2, 154.7, 149.5, 146.1, 144.1, 135.7, 129.5, 128.9, 128.7, 128.5, 128.4, 119.1, 118.6, 113.8, 113.5, 75.4, 67.9, 67.5, 62.0, 55.7, 14.1, 12.0.

HRMS (ESI-Orbitrap, *m/z*): $[M+Na]^+$ Calcd for $C_{28}H_{30}N_4NaO_4$ 509.2165; Found 509.2161.

Benzyl-4-(1-(2-(methoxycarbonyl)hydrazineylidene)ethyl)-1,3-diphenylimidazolidine-4-carboxylate (3h): compound **3h** was obtained by column chromatography (cycloehexane/ethyl acetate 8:2) in **62%** yield (87.6 mg), 12 h; yellow oil.

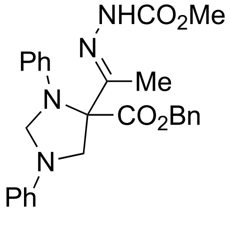

^{1}H NMR (400 MHz, CDCl$_3$) δ 8.06 (s, 1H), 7.34 – 7.30 (m, 5H), 7.24 – 7.18 (m, 4H), 6.94 – 6.87 (m, 1H), 6.85 – 6.80 (m, 3H), 6.75 – 6.70 (m, 2H), 5.28 (d, *J* = 12.5 Hz, 1H), 5.23 (d, *J* = 12.5 Hz, 1H), 4.94 (d, *J* = 3.2 Hz, 1H), 4.85 (d, *J* = 3.2 Hz, 1H), 4.49 (d, *J* = 9.3 Hz, 1H), 3.84 (s, 3H), 3.52 (d, *J* = 9.2 Hz, 1H), 1.78 (s, 3H).

^{13}C{^{1}H} NMR (101 MHz, CDCl$_3$) δ 170.1, 154.1, 149.2, 145.9, 143.9, 135.6, 129.4, 128.9, 128.3, 128.1, 127.9, 119.0, 118.6, 113.8, 113.4, 75.5, 67.6, 67.4, 55.8, 53.1, 11.8.

HRMS (ESI-Orbitrap, *m/z*): $[M+Na]^+$ Calcd for $C_{27}H_{28}N_4NaO_4$ 495.2008; Found 495.1993.

Methyl-4-(1-(2-(*tert*-butoxycarbonyl)hydrazineylidene)butyl)-1,3-diphenylimidazolidine-4-carboxylate (3i): compound **3i** was obtained by column chromatography (cycloehexane/ethyl acetate 9:1) in **97%** yield (135.5 mg), 12 h; white solid; mp: 151–153 °C.

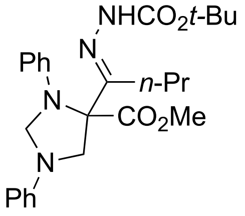

^{1}H NMR (400 MHz, CDCl$_3$) δ 7.76 (s, 1H), 7.35 (t, *J* = 7.3 Hz, 2H), 7.24 (t, *J* = 7.3 Hz, 2H), 6.89 (t, *J* = 7.3 Hz, 1H), 6.88 – 6.80 (m, 3H), 6.74 (d, *J* = 7.3 Hz, 2H), 4.92 (d, *J* = 3.2 Hz, 1H), 4.85 (d, *J* = 3.2 Hz, 1H), 4.44 (d, *J* = 9.2 Hz, 1H), 3.81 (s, 3H), 3.57 (d, *J* = 9.2 Hz, 1H), 2.22 – 1.99 (m, 2H), 1.62 – 1.50 (m, 11H), 0.88 (t, *J* = 7.3 Hz, 3H).

^{13}C{^{1}H} NMR (101 MHz, CDCl$_3$) δ 171.1, 152.3, 150.6, 146.0, 144.2, 129.4, 128.8, 119.1, 118.5, 114.1, 113.5, 81.7, 75.9, 67.5, 65.8, 55.5, 52.6, 28.3, 18.9, 14.8.

HRMS (ESI-Orbitrap, *m/z*): $[M+Na]^+$ Calcd for $C_{26}H_{34}N_4NaO_4$ 489.2478; Found 489.2463.

2-Methoxyethyl-4-(1-(2-(ethoxycarbonyl)hydrazineylidene)ethyl)-1,3-diphenylimidazolidine-4-carboxylate (3j): compound **3j** was obtained by column chromatography (cycloehexane/ethyl acetate 8:2) in **78%** yield (106.4 mg), 12 h; white solid; mp: 163–165 °C.

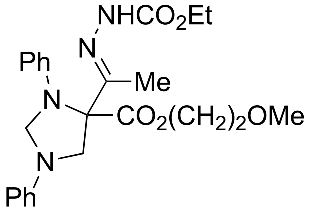

^{1}H NMR (400 MHz, DMSO-*d*$_6$) δ 10.16 (s, 1H), 7.27 (t, *J* = 7.9 Hz, 2H), 7.27 (t, *J* = 7.9 Hz, 2H), 6.89 – 6.79 (m, 5H), 6.73 (t, *J* = 7.9 Hz, 1H), 4.89 (d, *J* = 4.0 Hz, 1H), 4.69 (d, *J* = 4.0 Hz, 1H), 4.36 (d, *J* = 9.6 Hz, 1H), 4.29 – 4.11 (m, 4H), 3.61 – 3.49 (m, 2H), 3.47 (d, *J* = 9.6 Hz, 1H), 3.18 (s, 3H), 1.72 (s, 3H), 1.25 (t, *J* = 7.1 Hz, 3H).

$^{13}C\{^1H\}$ NMR (101 MHz, DMSO-*d*$_6$) δ 170.8, 154.3, 150.3, 146.6, 144.5, 129.5, 128.8, 119.0, 118.0, 114.3, 114.1, 75.9, 69.9, 67.7, 64.6, 61.1, 58.4, 55.3, 15.0, 13.6.

HRMS (ESI-Orbitrap, *m/z*): $[M+Na]^+$ Calcd for $C_{24}H_{30}N_4NaO_5$ 477.2114; Found 477.2123.

Ethyl-4-(1-(2-carbamoylhydrazineylidene)ethyl)-1,3-diphenylimidazolidine-4-carboxylate (3k): compound **3k** was obtained by column chromatography (cycloehexane/ethyl acetate 6:4) in **78%** yield (92.5 mg), 12 h; white solid; mp: 173–175 °C.

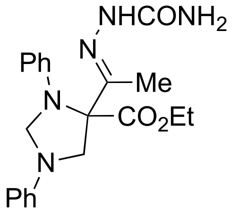

^1H NMR (400 MHz, DMSO-*d*$_6$) δ 9.50 (s, 1H), 7.26 (t, *J* = 7.9 Hz, 2H), 7.21 (bs, 1H), 7.17 (t, *J* = 7.4 Hz, 2H), 6.90 – 6.75 (m, 6H), 6.72 (t, *J* = 7.4 Hz, 1H), 4.86 (d, *J* = 4.0 Hz, 1H), 4.71 (d, *J* = 4.0 Hz, 1H), 4.25 (d, *J* = 9.6 Hz, 1H), 4.22 – 4.05 (m, 2H), 3.57 (d, *J* = 9.6 Hz, 1H), 1.75 (s, 3H), 1.13 (t, *J* = 7.1 Hz, 3H).

$^{13}C\{^1H\}$ NMR (101 MHz, DMSO-*d*$_6$) δ 170.5, 156.6, 146.1, 144.9, 144.1, 129.1, 128.4, 118.4, 117.5, 113.7, 113.4, 75.0, 67.2, 61.2, 55.4, 13.9, 13.3.

HRMS (ESI-Orbitrap, *m/z*): $[M+Na]^+$ Calcd for $C_{21}H_{25}N_5NaO_3$ 418.1855; Found 418.1894.

Ethyl-1,3-diphenyl-4-(1-(2-(phenylcarbamoyl)hydrazineylidene)ethyl)imidazolidine-4-carboxylate (3l): compound **3l** was obtained by column chromatography (cycloehexane/ethyl acetate 8:2) in **71%** yield (102.2 mg), 12 h; white solid; mp: 179–181 °C.

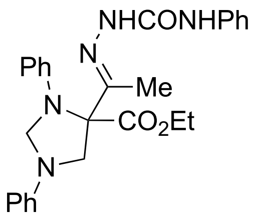

^{1}H NMR (400 MHz, DMSO-*d$_6$*) δ 10.00 (s, 1H), 8.01 (s, 1H), 7.34 – 7.17 (m, 8H), 7.00 (tt, *J* = 7.5, 1.4 Hz, 1H), 6.90 – 6.73 (m, 6H), 4.88 (d, *J* = 4.0 Hz, 1H), 4.84 (d, *J* = 4.0 Hz, 1H), 4.40 – 4.14 (m, 3H), 3.78 (d, *J* = 9.7 Hz, 1H), 1.92 (s, 3H), 1.15 (t, *J* = 7.1 Hz, 3H).

^{13}C{^{1}H} NMR (101 MHz, DMSO-*d$_6$*) δ 171.0, 153.2, 146.4, 144.7, 138.8, 129.6, 129.3, 129.1, 123.0, 118.9, 118.8, 118.1, 114.1, 113.8, 74.9, 67.8, 62.0, 56.4, 26.8, 14.7, 14.4.

HRMS (ESI-Orbitrap, *m/z*): [M+Na]$^+$ Calcd for $C_{27}H_{29}N_5NaO_3$ 494.2168; Found 494.2163.

Ethyl-4-(1-(2-(tert-butoxycarbonyl)hydrazineylidene)ethyl)-1,3-bis(4-methoxyphenyl) imidazolidine-4-carboxylate (3m): compound **3m** was obtained by column chromatography (cycloehexane/ethyl acetate 8:2) in **90%** yield (143.5 mg), 12 h; pink solid; mp: 187–189 °C.

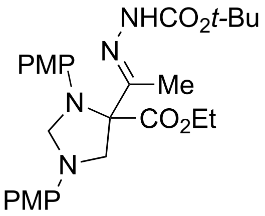

^{1}H NMR (400 MHz, CDCl$_3$) δ 7.69 (s, 1H), 6.88 (d, *J* = 9.0 Hz, 2H), 6.83– 6.76 (m, 4H), 6.68 (d, *J* = 9.0 Hz, 2H), 4.85 (d, *J* = 3.1 Hz, 1H), 4.68 (d, *J* = 3.1 Hz, 1H), 4.39 (d, *J* = 9.2 Hz, 1H), 4.35 – 4.14 (m, 2H), 3.77 (s, 3H), 3.75 (s, 3H), 3.42 (d, *J* = 9.2 Hz, 1H), 1.67 (s, 3H), 1.52 (s, 9H), 1.27 (t, *J* = 7.1 Hz, 3H).

^{13}C{^{1}H} NMR (101 MHz, CDCl$_3$) δ 170.7, 153.2, 152.6, 152.5, 148.3, 140.9, 138.3, 115.1, 115.0, 114.9, 114.4, 81.6, 75.8, 68.8, 61.8, 56.7, 55.8, 55.7, 28.3, 14.3, 12.1.

HRMS (ESI-Orbitrap, *m/z*): [M+Na]$^+$ Calcd for $C_{27}H_{36}N_4NaO_6$ 535.2533; Found 535.2519.

Methyl-4-(1-(2-(*tert*-butoxycarbonyl)hydrazineylidene)ethyl)-1,3-bis(4-methoxyphenyl) imidazolidine-4-carboxylate (3n): compound **3n** was obtained by column chromatography (cycloehexane/ethyl acetate 8:2) in **82%** yield (122.3 mg), 12 h; white solid; mp: 163–165 °C.

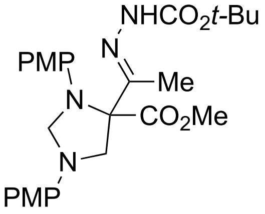

^{1}H NMR (400 MHz, DMSO-*d*$_6$) δ 9.80 (s, 1H), 6.94 – 6.68 (m, 8H), 4.81 (d, *J* = 3.8 Hz, 1H), 4.50 (d, *J* = 3.8 Hz, 1H), 4.28 (d, *J* = 9.5 Hz, 1H), 3.69 (s, 3H), 3.32 (d, *J* = 9.8 Hz, 1H), 3.67 (s, 3H), 3.65 (s, 3H), 1.62 (s, 3H), 1.47 (s, 9H).

^{13}C{^{1}H} NMR (101 MHz, DMSO-*d*$_6$) δ 171.3, 152.6, 151.6, 140.7, 138.1, 119.2, 115.2, 114.6, 114.5, 114.2, 114.0, 79.6, 75.9, 68.4, 55.8, 55.3, 55.2, 52.0, 28.1, 13.2.

HRMS (ESI-Orbitrap, *m/z*): $[M+Na]^+$ Calcd for $C_{26}H_{34}N_4NaO_6$ 521.2376; Found 521.2354

Ethyl-4-(1-(2-(ethoxycarbonyl)hydrazineylidene)ethyl)-1,3-bis(4-methoxyphenyl)imidazolidine-4-carboxylate (3o): compound **3o** was obtained by column chromatography (cycloehexane/ethyl acetate 8:2) in **52%** yield (75.5 mg), 12 h; white solid; mp: 155–157 °C.

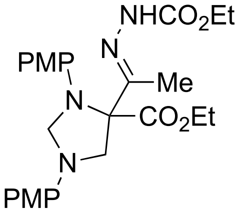

^{1}H NMR (400 MHz, DMSO-d6) δ 10.07 (s, 1H), 6.87 (d, *J* = 9.0 Hz, 2H), 6.81 (d, *J* = 9.0 Hz, 2H), 6.78 – 6.74 (m, 4H), 4.80 (d, *J* = 3.8 Hz, 1H), 4.52 (d, *J* = 3.8 Hz, 1H), 4.27 (d, *J* = 9.4 Hz, 1H), 4.18 – 4.07 (m, 4H), 3.69 (s, 3H), 3.67 (s, 3H), 3.34 (d, *J* = 9.4 Hz, 1H), 1.64 (s, 3H), 1.23 (t, *J* = 7.1 Hz, 3H), 1.20 (t, *J* = 7.1 Hz, 3H).

^{13}C{^{1}H} NMR (101 MHz, DMSO-d6) δ 170.6, 153.8, 152.6, 151.6, 150.0, 140.7, 138.1, 115.2, 114.5 (2C), 113.9, 75.7, 68.4, 60.8, 60.6, 55.9, 55.3, 55.2, 14.5, 14.0, 13.3.

HRMS (ESI-Orbitrap, *m/z*): $[M+Na]^+$ Calcd for $C_{25}H_{32}N_4NaO_6$ 507.2220; Found 507.2236

Ethyl-4-(1-(2-(*tert*-butoxycarbonyl)hydrazineylidene)ethyl)-1,3-di-*p*-tolylimidazolidine-4-carboxylate (3p): compound **3p** was obtained by column chromatography (cycloehexane/ethyl acetate 9:1) in **21%** yield (30.3 mg), 12 h; colorless oil.

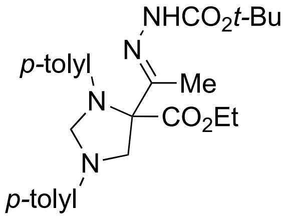

^{1}H NMR (400 MHz, CDCl$_3$) δ 7.61 (s, 1H), 7.11 (d, *J* = 8.3 Hz, 2H), 7.02 (d, *J* = 8.3 Hz, 2H), 6.72 (d, *J* = 8.3 Hz, 2H), 6.63 (d, *J* = 8.3 Hz, 2H), 4.88 (d, *J* = 3.1 Hz, 1H), 4.74 (d, *J* = 3.1 Hz, 1H), 4.43 (d, *J* = 9.2 Hz, 1H), 4.35 – 4.18 (m, 2H), 3.43 (d, *J* = 9.2 Hz, 1H), 2.29 (s, 3H), 2.25 (s, 3H), 1.69 (s, 3H), 1.53 (s, 9H), 1.27 (t, *J* = 7.1 Hz, 3H).

^{13}C{^{1}H} NMR (101 MHz, CDCl$_3$) δ 170.3, 144.1, 143.7, 141.8, 129.8, 129.7, 129.6, 128.2, 127.4, 113.7, 113.6, 81.5, 75.3, 67.9, 61.8, 56.1, 26.9, 20.4, 20.3, 14.2, 11.7.

HRMS (ESI-Orbitrap, *m/z*): $[M+Na]^+$ Calcd for $C_{27}H_{36}N_4NaO_4$ 503.2634; Found 503.2625.

Ethyl-4-(1-(2-(*tert*-butoxycarbonyl)hydrazineylidene)ethyl)-1,3-bis(4-chlorophenyl)imidazolidine-4-carboxylate (3q): compound **3q** was obtained by column chromatography (cycloehexane/ethyl acetate 9:1) in **55%** yield (85.8 mg), 12 h; colorless oil.

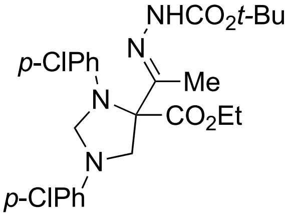

^{1}H NMR (400 MHz, CDCl$_3$) δ 7.70 (s, 1H), 7.25 (d, *J* = 9.1 Hz, 2H), 7.16 (d, *J* = 9.1 Hz, 2H), 6.73 (d, *J* = 9.1 Hz, 2H), 6.61 (d, *J* = 9.1 Hz, 2H), 4.85 (d, *J* = 3.2 Hz, 1H), 4.74 (d, *J* = 3.2 Hz, 1H), 4.42 (d, *J* = 9.4 Hz, 1H), 4.34 – 4.18 (m, 2H), 3.45 (d, *J* = 9.4 Hz, 1H), 1.72 (s, 3H), 1.52 (s, 9H), 1.26 (t, *J* = 7.2 Hz, 3H).

^{13}C{^{1}H} NMR (101 MHz, CDCl$_3$) δ 169.9, 152.2, 144. 5, 144.4, 142.5, 129.3, 128.7, 124.1, 123.6, 115.0, 114.5, 81.8, 75.4, 67.5, 62.1, 55.7, 28.3, 14.2, 11.8.

HRMS (ESI-Orbitrap, *m/z*): $[M+Na]^+$ Calcd for $C_{25}H_{30}Cl_2N_4NaO_4$ 543.1542; Found 543.1554.

Ethyl-4-(1-(2-(*tert*-butoxycarbonyl)hydrazineylidene)ethyl)-1,3-bis(4-fluorophenyl)imidazolidine-4-carboxylate (3r): compound **3r** was obtained by column chromatography (cycloehexane/ethyl acetate 9:1) in **67%** yield (98.0 mg), 12 h; white solid; mp: 171–173 °C.

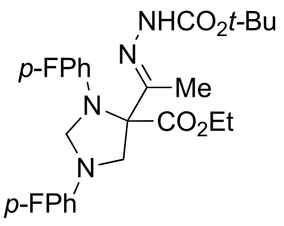

^{1}H NMR (400 MHz, DMSO-*d*$_6$) δ 9.80 (s, 1H), 7.10 (t, *J* = 8.6 Hz, 2H), 7.01 (t, *J* = 8.6 Hz, 2H), 6.91 – 6.83 (m, 2H), 6.83 – 6.75 (m, 2H), 4.84 (d, *J* = 4.0 Hz, 1H), 4.61 (d, *J* = 4.0 Hz, 1H), 4.31 (d, *J* = 9.6 Hz, 1H), 4.21 – 4.03 (m, 2H), 3.41 (d, *J* = 9.6 Hz, 1H), 1.67 (s, 3H), 1.46 (s, 9H), 1.18 (t, *J* = 7.0 Hz, 3H).

^{13}C{^{1}H} NMR (101 MHz, DMSO-*d*$_6$) δ 170.8, 156.4 (d, J_{CF} = 234.6 Hz), 155.7 (d, J_{CF} = 233.8 Hz), 153.1, 149.3, 143.5, 141.2, 116.0 (d, J_{CF} = 22.0 Hz), 115.6 (d, J_{CF} = 7.4 Hz), 115.2 (d, J_{CF} = 21.9 Hz), 115.1 (d, J_{CF} = 7.1 Hz), 80.0, 76.1, 68.5, 61.5, 56.0, 28.5, 14.4, 13.6.

^{19}F{^{1}H} NMR (376 MHz, DMSO-*d*$_6$) δ -126.2, -127.7.

HRMS (ESI-Orbitrap, *m/z*): $[M+Na]^+$ Calcd for $C_{25}H_{30}F_2N_4NaO_4$ 511.2133; Found 511.2124.

5. Procedure for scale-up reaction.

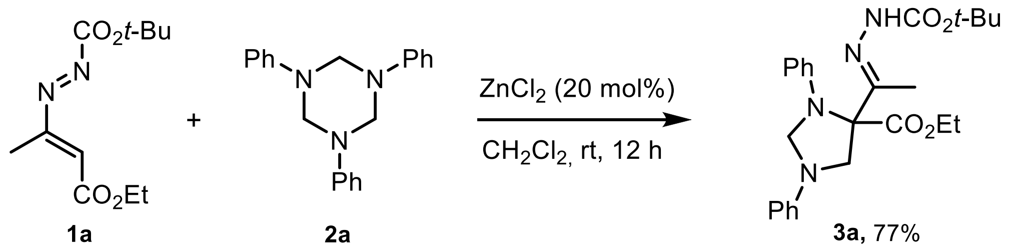

To a solution of 1,2-diaza-1,3-diene **1a** (484.5 mg, 2 mmol, 2 equiv) in CH_2Cl_2 (10 mL), 1,3,5-triaryl-1,3,5-triazinane **2** (315.4 mg, 1 mmol) and $ZnCl_2$ (27.3 mg, 0.2 mmol, 20 mol %) were added. After stirring for 12 h (TLC monitoring) at rt, the solvent was removed and the crude mixture directly purified through flash column chromatography on silica gel with cycloehexane/ethyl acetate (8:2, v/v) as the eluent, yielding compound **3a** (348.9 mg, 77%).

6. Synthesis and characterization of differently substituted 1,3-diaryl imidazolidines 3.

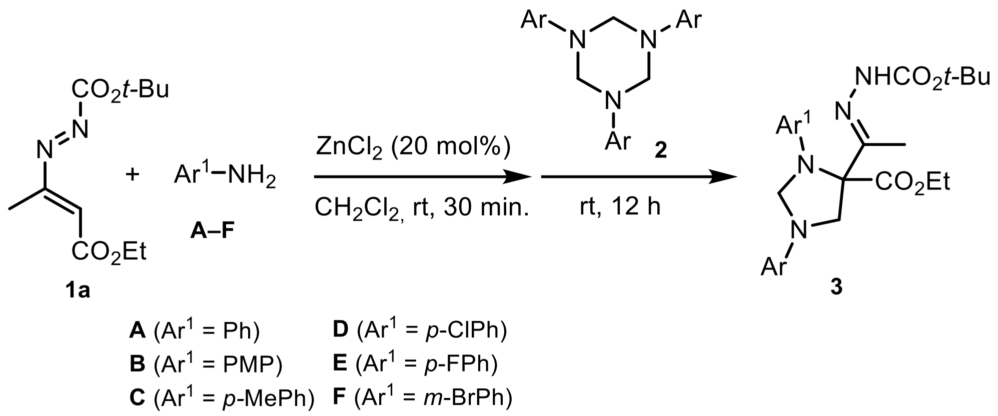

*6.1. Synthesis of differently substituted 1,3-diaryl imidazolidines **3** from 1,2-diaza-1,3-diene **1a**, aromatic amines **A–F** and 1,3,5-triaryl-1,3,5-triazinanes **2**.* To a solution of 1,2-diaza-1,3-diene **1a** (0.3 mmol) in CH_2Cl_2 (3 mL), aromatic amines **A–F** (0.3 mmol, 1 equiv) and $ZnCl_2$ (8.2 mg, 0.06 mmol, 20 mol %) were added, and then the mixture was stirred at rt for 30 min (TLC monitoring). After that, 1,3,5-triaryl-1,3,5-triazinanes **2** (0.3 mmol, 1 equiv) was added and continued stirring for another 12 h (TLC monitoring). Then, the solvent was removed and the crude mixture directly purified through flash column chromatography on silica gel to afford the desired imidazolidine **3**.

*6.2 Characterization of differently substituted 1,3-diaryl imidazolidines **3Ab–Fa**:*

Ethyl-4-(1-(2-(*tert*-butoxycarbonyl)hydrazineylidene)ethyl)-1-(4-methoxyphenyl)-3-phenylimidazolidine-4-carboxylate (3Ab): compound **3Ab** was obtained by column chromatography (cycloehexane/ethyl acetate 8:2) in **84%** yield (121.3 mg), 12 h; white solid; mp: 155–157 °C.

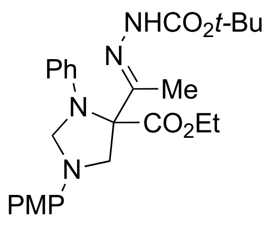

^{1}H NMR (400 MHz, DMSO-*d*$_6$) δ 9.79 (s, 1H), 7.15 (t, J = 8.2, 2H), 6.92 – 6.78 (m, 6H), 6.71 (t, J = 7.2 Hz, 1H), 4.88 (d, J = 4.1 Hz, 1H), 4.54 (d, J = 4.1 Hz, 1H), 4.30 (d, J = 9.4 Hz, 1H), 4.21 – 4.06 (m, 2H), 3.71 (s, 3H), 3.34 (d, J = 9.4 Hz, 1H), 1.66 (s, 3H), 1.47 (s, 9H), 1.20 (t, J = 7.1 Hz, 3H).

$^{13}C\{^1H\}$ NMR (101 MHz, DMSO-*d*$_6$) δ 170.9, 153.2, 153.1, 149.6, 144.5, 141.1, 128.8, 117.8, 115.8, 115.0, 113.9, 80.0, 76.0, 68.6, 61.4, 56.4, 55.8, 28.5, 14.4, 13.7.

HRMS (ESI-Orbitrap, *m/z*): $[M+Na]^+$ Calcd for $C_{26}H_{34}N_4NaO_5$ 505.2427; Found 505.2419.

Ethyl-4-(1-(2-(*tert*-butoxycarbonyl)hydrazineylidene)ethyl)-3-phenyl-1-(*p*-tolyl)imidazolidine-4-carboxylate (3Ac): compound **3Ac** was obtained by column chromatography (cycloehexane/ethyl acetate 8:2) in **55%** yield (80.2 mg), 12 h; white solid; mp: 110–113 °C.

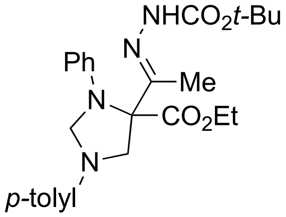

^1H NMR (400 MHz, DMSO-*d*$_6$) δ 9.80 (s, 1H), 7.15 (t, *J* = 8.1, 2H), 7.09 (d, *J* = 8.1 Hz, 2H), 6.85 – 6.68 (m, 5H), 4.87 (d, *J* = 4.1 Hz, 1H), 4.60 (d, *J* = 4.1 Hz, 1H), 4.32 (d, *J* = 9.6 Hz, 1H), 4.21 – 4.02 (m, 2H), 3.38 (d, *J* = 9.6 Hz, 1H), 2.24 (s, 3H), 1.68 (s, 3H), 1.47 (s, 9H), 1.19 (t, *J* = 7.1 Hz, 3H).

$^{13}C\{^1H\}$ NMR (101 MHz, DMSO-*d*$_6$) δ 170.8, 153.1, 149.6, 144.6, 144.5, 130.0, 128 8, 127.8, 117.8, 114.5, 114.0, 80.0, 75.9, 68.0, 61.4, 55.8, 28.5, 20.6, 14.4, 13.7.

HRMS (ESI-Orbitrap, *m/z*): $[M+Na]^+$ Calcd for $C_{26}H_{34}N_4NaO_4$ 489.2478; Found 489.2474.

Ethyl (*E/Z*)-4-(1-(2-(*tert*-butoxycarbonyl)hydrazineylidene)ethyl)-1-(4-chlorophenyl)-3-phenylimidazolidine-4-carboxylate (3Ad): compound **3Ad** was obtained by column chromatography (cycloehexane/ethyl acetate 8:2) in **62%** yield (90.3 mg), 12 h; white solid; mp: 172–175 °C.

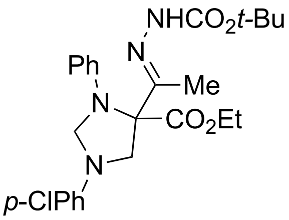

^1H NMR (400 MHz, DMSO-*d*$_6$), mixture of *E/Z* isomers (ratio major/minor = 55/45) δ 9.86 – 9.78 (bs, 1H), 7.33 – 7.23 (m, 2H), 7.16 (t, *J* = 7.8 Hz, 2H), 6.93 – 6.78 (m, 4H), 6.73 (t, *J* = 7.8 Hz, 1H), 4.88 and

4.85 (d, J = 4.1 Hz, and d, J = 4.2 Hz, 1H), 4.69 and 4.67 (d, J = 4.2 Hz, and d, J = 4.1 Hz, 1H), 4.36 and 4.33 (d, J = 9.4 Hz, and d, J = 9.7 Hz, 1H), 4.20 – 4.05 (m, 2H), 3.46 and 3.44 (d, J = 9.7 Hz, and d, J = 9.4 Hz, 1H), 1.71 (s, 3H), 1.47 (s, 9H), 1.18 and 1.17 (t, J = 7.1 Hz, and t, J = 7.1 Hz, 3H).

$^{13}C\{^1H\}$ NMR (101 MHz, DMSO-*d*$_6$) for clarity, the signals (some of which overlapping) of both isomers are reported in sequence: δ 170.7, 170.6, 153.1, 149.6, 146.6, 145.4, 144.6, 144.5, 129.5, 129.2, 128.8, 122.6, 119.0, 118.0, 117.9, 115.7, 114.3, 114.1, 114.0, 80.0, 75.8, 75.7, 67.7, 67.6, 61.5, 61.4, 55.4, 28.5, 14.5, 14.4, 13.6, 13.5.

HRMS (ESI-Orbitrap, *m/z*): $[M+Na]^+$ Calcd for $C_{25}H_{31}ClN_4NaO_4$ 509.1932; Found 509.1923.

Ethyl-4-(1-(2-(*tert*-butoxycarbonyl)hydrazineylidene)ethyl)-1-(4-fluorophenyl)-3-phenylimidazolidine-4-carboxylate (3Ae): compound **3Ae** was obtained by column chromatography (cycloehexane/ethyl acetate 8:2) in **67%** yield (94.5 mg), 12 h; colorless oil.

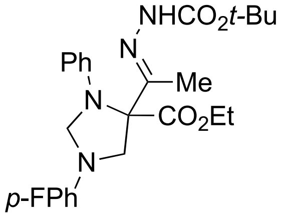

^1H NMR (400 MHz, DMSO-*d*$_6$) δ 9.80 (s, 1H), 7.19 – 7.05 (m, 4H), 6.92 – 6.78 (m, 4H), 6.71 (t, J = 7.3 Hz, 1H), 4.87 (d, J = 4.0 Hz, 1H), 4.62 (d, J = 4.0 Hz, 1H), 4.32 (d, J = 9.6 Hz, 1H), 4.19 – 4.05 (m, 2H), 3.40 (d, J = 9.6 Hz, 1H), 1.68 (s, 3H), 1.46 (s, 9H), 1.18 (t, J = 7.1 Hz, 3H).

$^{13}C\{^1H\}$ NMR (101 MHz, DMSO-*d*$_6$) δ 170.7, 156.4 (d, J_{CF} = 234.9 Hz), 153.1, 149.6, 144.5, 143.5 (d, J_{CF} = 1.7 Hz), 128.8, 117.9, 116.0 (d, J_{CF} = 22.0 Hz), 115.6 (d, J_{CF} = 7.5 Hz), 114.0, 80.0, 75.9, 68.2, 61.4, 56.0, 28.5, 14.4, 13.6.

$^{19}F\{^1H\}$ NMR (376 MHz, DMSO-*d*$_6$) δ -126.1.

HRMS (ESI-Orbitrap, *m/z*): $[M+Na]^+$ Calcd for $C_{25}H_{31}FN_4NaO_4$ 493.2227; Found 493.2221.

Ethyl-4-(1-(2-(*tert*-butoxycarbonyl)hydrazineylidene)ethyl)-3-(4-methoxyphenyl)-1-phenylimidazolidine-4-carboxylate (3Ba): compound **3Ba** was obtained by column chromatography (cycloehexane/ethyl acetate 8:2) in **77%** yield (111.2 mg), 12 h; white solid; mp: 137–139 °C.

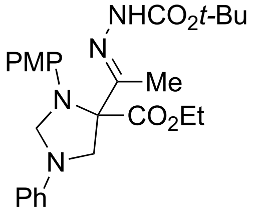

^{1}H NMR (400 MHz, DMSO-*d*$_6$) δ 9.78 (s, 1H), 7.14 (t, *J* = 7.9 Hz, 2H), 6.91 – 6.77 (m, 6H), 6.70 (t, *J* = 7.2 Hz, 1H), 4.87 (d, *J* = 4.0 Hz, 1H), 4.53 (d, *J* = 4.0 Hz, 1H), 4.29 (d, *J* = 9.4 Hz, 1H), 4.21 – 4.03 (m, 2H), 3.70 (s, 3H), 3.33 (d, *J* = 9.4 Hz, 1H), 1.65 (s, 3H), 1.46 (s, 9H), 1.19 (t, *J* = 7.1 Hz, 3H).

^{13}C{^{1}H} NMR (101 MHz, DMSO-*d*$_6$) δ 170.4, 152.7, 152.6, 149.2, 144.1, 140.6, 128.3, 117.3, 115.4, 114.5, 113.4, 79.5, 75.6, 68.1, 60.9, 55.9, 55.3, 28.1, 14.0, 13.3.

HRMS (ESI-Orbitrap, *m/z*): $[M+Na]^+$ Calcd for $C_{26}H_{34}N_4NaO_5$ 505.2427; Found 505.2429.

Ethyl-4-(1-(2-(*tert*-butoxycarbonyl)hydrazineylidene)ethyl)-3-(4-methoxyphenyl)-1-(p-tolyl)imidazolidine-4-carboxylate (3Bc): compound **3Bc** was obtained by column chromatography (cycloehexane/ethyl acetate 8:2) in **87%** yield (129.5 mg), 12 h; white solid; mp: 137–139 °C.

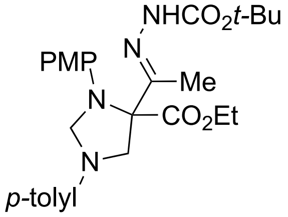

^{1}H NMR (400 MHz, DMSO-*d*$_6$) δ 9.76 (s, 1H), 7.07 (d, *J* = 8.2 Hz, 2H), 6.82 – 6.69 (m, 4H), 6.74 (d, *J* = 8.2 Hz, 2H), 4.79 (d, *J* = 3.9 Hz, 1H), 4.58 (d, *J* = 3.9 Hz, 1H), 4.28 (d, *J* = 9.6 Hz, 1H), 4.16 – 4.05 (m, 2H), 3.67 (s, 3H), 3.37 (d, *J* = 9.6 Hz, 1H), 2.22 (s, 3H), 1.66 (s, 3H), 1.46 (s, 9H), 1.17 (t, *J* = 7.1 Hz, 3H).

^{13}C{^{1}H} NMR (101 MHz, DMSO-*d*$_6$) δ 170.5, 152.6, 151.6, 149.3, 144.2, 138.2, 129.5, 127.1, 114.7, 113.9, 113.8, 79.5, 75.5, 67.8, 60.8, 55.3, 55.2, 28.1, 20.1, 14.0, 13.2.

HRMS (ESI-Orbitrap, *m/z*): $[M+Na]^+$ Calcd for $C_{27}H_{36}N_4NaO_5$ 519.2583; Found 519.2575.

Ethyl-4-(1-(2-(*tert*-butoxycarbonyl)hydrazineylidene)ethyl)-1-(4-chlorophenyl)-3-(4-methoxyphenyl)imidazolidine-4-carboxylate (3Bd): compound **3Bd** was obtained by column chromatography (cycloehexane/ethyl acetate 8:2) in **52%** yield (80.5 mg), 12 h; white solid; mp: 146–148 °C.

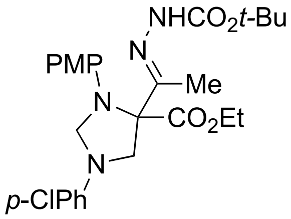

^{1}H NMR (400 MHz, DMSO-*d*$_6$) δ 9.78 (s, 1H), 7.28 (d, *J* = 9.0 Hz, 2H), 6.83 (d, *J* = 9.0 Hz, 2H), 6.80 – 6.76 (m, 4H), 4.76 (d, *J* = 4.1 Hz, 1H), 4.66 (d, *J* = 4.1 Hz, 1H), 4.28 (d, *J* = 9.7 Hz, 1H), 4.18 – 4.01 (m, 2H), 3.67 (s, 3H), 3.45 (d, *J* = 9.7 Hz, 1H), 1.69 (s, 3H), 1.46 (s, 9H), 1.16 (t, *J* = 7.1 Hz, 3H).

^{13}C{^{1}H} NMR (101 MHz, DMSO-*d*$_6$) δ 170.3, 152.6, 151.8, 149.1, 144.9, 138.1, 128.7, 121.9, 115.0 (2C), 113.9, 79.5, 75.5, 67.4, 60.9, 55.2, 54.8, 28.1, 13.9, 13.1.

HRMS (ESI-Orbitrap, *m/z*): $[M+Na]^+$ Calcd for $C_{26}H_{33}ClN_4NaO_5$ 539.2037; Found 539.2023.

Ethyl-4-(1-(2-(*tert*-butoxycarbonyl)hydrazineylidene)ethyl)-1-(4-fluorophenyl)-3-(4-methoxyphenyl)imidazolidine-4-carboxylate (3Be): compound **3Be** was obtained by column chromatography (cycloehexane/ethyl acetate 8:2) in **63%** yield (94.5 mg), 12 h; colorless oil.

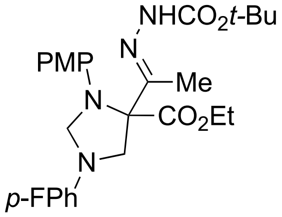

^{1}H NMR (400 MHz, DMSO-*d*$_6$ δ 9.78 (s, 1H), 7.11 (t, *J* = 8.9 Hz, 2H), 6.93 – 6.82 (m, 2H), 6.80 – 6.72 (m, 4H), 4.81 (d, *J* = 3.9 Hz, 1H), 4.61 (d, *J* = 3.9 Hz, 1H), 4.30 (d, *J* = 9.6 Hz, 1H), 4.21 – 4.04 (m, 2H), 3.68 (s, 3H), 3.41 (d, *J* = 9.6 Hz, 1H), 1.68 (s, 3H), 1.47 (s, 9H), 1.18 (t, *J* = 7.1 Hz, 3H).

^{13}C{^{1}H} NMR (101 MHz, DMSO-*d*$_6$) δ 170.5, 155.8 (d, J_{CF} = 234.6 Hz), 152.7, 151.7, 149.2, 143.1 (d, J_{CF} = 1.8 Hz), 138.1, 115.5 (d, J_{CF} = 22.1 Hz), 114.9 (d, J_{CF} = 7.5 Hz), 114.8, 113.9, 79.5, 75.6, 68.0, 60.8, 55.5, 55.2, 28.1, 14.0, 13.2.

^{19}F NMR (376 MHz, DMSO-*d6*) δ -126.4.

HRMS (ESI-Orbitrap, *m/z*): $[M+Na]^+$ Calcd for $C_{26}H_{33}FN_4NaO_5$ 523.2333; Found 523.2351.

Ethyl (*E/Z*)-4-(1-(2-(*tert*-butoxycarbonyl)hydrazineylidene)ethyl)-1-phenyl-3-(*p*-tolyl)imidazolidine-4-carboxylate (3Ca): compound **3Ca** was obtained by column chromatography (cycloehexane/ethyl acetate 8:2) in **58%** yield (81.1mg), 12 h; white solid; mp: 128–131 °C.

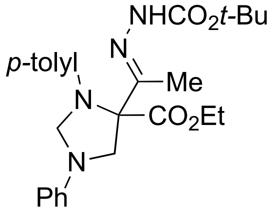

^{1}H NMR (400 MHz, DMSO-*d*$_6$), mixture of *E/Z* isomers (ratio major/minor = 68/32) δ 9.79 and 9.77 (s, 1H), 7.30 – 7.04 (m, 2H) 7.00 – 6.93 (m, 2H), 6.88 – 6.69 (m, 5H), 4.84 and 4.83 (d, *J* = 4.1 Hz, and d, *J* = 4.0 Hz, 1H), 4.66 and 4.58 (d, *J* = 4.1 Hz, and d, *J* = 4.0 Hz, 1H), 4.34 and 4.30 (d, *J* = 9.6 Hz, and d, *J* = 9.6 Hz, 1H), 4.20 – 4.03 (m, 2H), 3.43 and 3.37 (d, *J* = 9.6 Hz, and d, *J* = 9.6 Hz, 1H), 2.23 and 2.20 (s, 3H), 1.69 and 1.66 (s, 3H), 1.48 (s, 9H), 1.19 (t, *J* = 7.1 Hz, 3H).

^{13}C{^{1}H} NMR (101 MHz, DMSO-*d*$_6$) δ for clarity, the signals of both isomers are reported in sequence: 170.4, 170.3, 152.7, 149.3, 146.2, 144.1, 141.8, 129.5, 129.0, 128.8, 127.2, 125.9, 125.8, 118.4, 113.9, 113.7, 113.6, 113.5, 79.5, 75.4, 75.3, 67.6, 67.3, 60.9, 60.8, 55.3, 54.9, 28.1, 20.1, 20.0, 14.0, 13.2, 13.1.

HRMS (ESI-Orbitrap, *m/z*): $[M+Na]^+$ Calcd for $C_{26}H_{34}N_4NaO_4$ 489.2478; Found 489.2498.

Ethyl-4-(1-(2-(*tert*-butoxycarbonyl)hydrazineylidene)ethyl)-1-(4-methoxyphenyl)-3-(p-tolyl)imidazolidine-4-carboxylate (3Cb): compound **3Cb** was obtained by column chromatography (cycloehexane/ethyl acetate 8:2) in **78%** yield (116.0 mg), 12 h; white solid; mp: 134-136 °C.

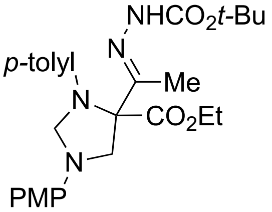

^{1}H NMR (400 MHz, DMSO-*d*$_6$) δ 9.76 (s, 1H), 6.96 (d, *J* = 8.6 Hz, 2H), 6.88 (d, *J* = 9.2 Hz, 2H), 6.83 (d, *J* = 9.2 Hz, 2H), 6.72 (d, *J* = 8.6 Hz, 2H), 4.84 (d, *J* = 4.0 Hz, 1H), 4.52 (d, *J* = 4.0 Hz, 1H), 4.29 (d, *J* = 9.5 Hz, 1H), 4.23 – 4.07 (m, 2H), 3.70 (s, 3H), 3.33 (d, *J* = 9.5 Hz, 1H), 2.19 (s, 3H), 1.64 (s, 3H), 1.48 (s, 9H), 1.20 (t, *J* = 7.1 Hz, 3H).

^{13}C{^{1}H} NMR (101 MHz, DMSO-*d*$_6$) δ 170.5, 152.7, 149.3, 141.8, 140.7, 128.8, 125.8, 119.0, 115.3, 114.5, 113.5, 79.5, 75.6, 68.2, 60.8, 55.9, 55.3, 28.1, 20.0, 14.0, 13.3.

HRMS (ESI-Orbitrap, *m/z*): $[M+Na]^+$ Calcd for $C_{27}H_{36}N_4NaO_5$ 519.2583; Found 519.2580.

Ethyl-4-(1-(2-(*tert*-butoxycarbonyl)hydrazineylidene)ethyl)-1-(4-chlorophenyl)-3-(*p*-tolyl)imidazolidine-4-carboxylate (3Cd): compound **3Cd** was obtained by column chromatography (cycloehexane/ethyl acetate 8:2) in **48%** yield (72.0 mg), 12 h; white solid; mp: 153–155 °C.

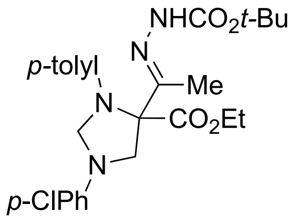

^{1}H NMR (400 MHz, DMSO-*d*$_6$) δ 9.79 (s, 1H), 7.29 (d, *J* = 8.9 Hz, 2H), 6.97 (d, *J* = 8.7 Hz, 2H), 6.86 (d, *J* = 8.9 Hz, 2H), 6.72 (d, *J* = 8.7 Hz, 2H), 4.81 (d, *J* = 4.2 Hz, 1H), 4.67 (d, *J* = 4.2 Hz, 1H), 4.32 (d, *J* = 9.7 Hz, 1H), 4.19 – 4.03 (m, 2H), 3.45 (d, *J* = 9.7 Hz, 1H), 2.20 (s, 3H), 1.69 (s, 3H), 1.47 (s, 9H), 1.17 (t, *J* = 7.1 Hz, 3H).

^{13}C{^{1}H} NMR (101 MHz, DMSO-*d*$_6$) δ 170.7, 153.1, 149.6, 145.4, 142.2, 129.3, 129.2, 126.6, 122.5, 115.7, 114.2, 80.0, 75.8, 67.7, 61.4, 55.4, 28.5, 20.4, 14.4, 13.6.

HRMS (ESI-Orbitrap, *m/z*): $[M+Na]^+$ Calcd for $C_{26}H_{33}ClN_4NaO_4$ 523.2083; Found 523,2076.

Ethyl-4-(1-(2-(*tert*-butoxycarbonyl)hydrazineylidene)ethyl)-1-(4-fluorophenyl)-3-(p-tolyl)imidazolidine-4-carboxylate (3Ce): compound **3Ce** was obtained by column chromatography (cycloehexane/ethyl acetate 8:2) in **55%** yield (80.0 mg), 12 h; colorless oil °C.

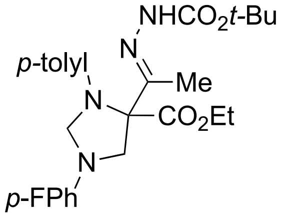

^{1}H NMR (400 MHz, DMSO-*d*$_6$) δ 9.78 (s, 1H), 7.23 – 7.02 (m, 2H), 6.96 (d, *J* = 8.3 Hz, 2H), 6.90 – 6.84 (m, 2H), 6.72 (d, *J* = 8.3 Hz, 2H), 4.84 (d, *J* = 4.1 Hz, 1H), 4.60 (d, *J* = 4.1 Hz, 1H), 4.31 (d, *J* = 9.6 Hz, 1H), 4.21 – 4.04 (m, 2H), 3.39 (d, *J* = 9.6 Hz, 1H), 2.19 (s, 3H), 1.66 (s, 3H), 1.47 (s, 9H), 1.19 (t, *J* = 7.1 Hz, 3H).

^{13}C{^{1}H} NMR (101 MHz, DMSO-*d*$_6$) δ 170.8, 156.3 (d, J_{CF} = 234.6 Hz), 153.1, 149.7, 143.6 (d, J_{CF} = 1.7 Hz), 142.2, 129.3, 126.4, 116.0 (d, J_{CF} = 22.1 Hz), 115.6 (d, J_{CF} = 7.5 Hz), 114.1, 80.0, 76.0, 68.3, 61.4, 56.0, 28.5 20.4, 14.5, 13.7.

HRMS (ESI-Orbitrap, *m/z*): $[M+Na]^+$ Calcd for $C_{26}H_{33}FN_4NaO_4$ 507.2384; Found 507.2369.

Ethyl-4-(1-(2-(*tert*-butoxycarbonyl)hydrazineylidene)ethyl)-3-(4-chlorophenyl)-1-phenylimidazolidine-4-carboxylate (3Da): compound **3Da** was obtained by column chromatography (cycloehexane/ethyl acetate 8:2) in **62%** yield (90.4 mg), 12 h; white solid; mp: 138–140 °C.

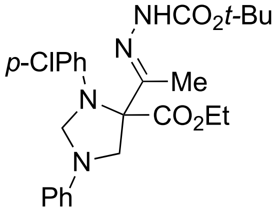

^{1}H NMR (400 MHz, DMSO-*d*$_6$) δ 9.83 (s, 1H), 7.27 (t, *J* = 8.7 Hz, 2H), 7.20 (d, *J* = 9.1 Hz, 2H), 6.90 – 6.79 (m, 5H), 4.88 (d, *J* = 4.2 Hz, 1H), 4.66 (d, *J* = 4.2 Hz, 1H), 4.36 (d, *J* = 9.7 Hz, 1H), 4.23 – 4.09 (m, 2H), 3.45 (d, *J* = 9.7 Hz, 1H), 1.71 (s, 3H), 1.47 (s, 9H), 1.20 (t, *J* = 7.1 Hz, 3H).

^{13}C{^{1}H} NMR (101 MHz, DMSO-*d*$_6$) δ 170.6, 153.1, 149.1, 146.5, 143.4, 129.5, 128.5, 121.7, 119.1, 115.6, 114.4, 80.1, 76.0, 67.8, 61.6, 55.4, 28.5, 14.4, 13.6.

HRMS (ESI-Orbitrap, *m/z*): $[M+Na]^+$ Calcd for $C_{25}H_{31}ClN_4NaO_4$ 509.1932; Found 509.1922.

Ethyl-4-(1-(2-(*tert*-butoxycarbonyl)hydrazineylidene)ethyl)-3-(4-chlorophenyl)-1-(4-methoxyphenyl)imidazolidine-4-carboxylate (3Db): compound **3Db** was obtained by column chromatography (cycloehexane/ethyl acetate 8:2) in **61%** yield (94.5 mg), 12 h; white solid; mp: 117–119 °C.

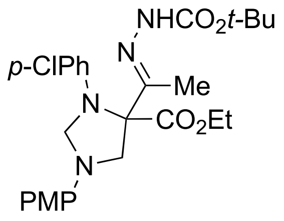

^{1}H NMR (400 MHz, DMSO-*d*$_6$) δ 9.80 (s, 1H), 7.18 (d, *J* = 9.1 Hz, 2H), 6.92 – 6.72 (m, 6H), 4.87 (d, *J* = 4.1 Hz, 1H), 4.51 (d, *J* = 4.1 Hz, 1H), 4.29 (d, *J* = 9.5 Hz, 1H), 4.21 – 4.09 (m, 2H), 3.70 (s, 3H), 3.34 (d, *J* = 9.5 Hz, 1H), 1.65 (s, 3H), 1.46 (s, 9H), 1.20 (t, *J* = 7.1 Hz, 3H).

^{13}C{^{1}H} NMR (101 MHz, DMSO-*d*$_6$) δ 170.7, 153.3, 153.1, 149.2, 143.4, 141.0, 128.5, 121.6, 115.9, 115.4, 115.0, 80.0, 76.1, 68.7, 61.5, 56.4, 55.8, 28.5, 14.4, 13.7.

HRMS (ESI-Orbitrap, *m/z*): $[M+Na]^+$ Calcd for $C_{26}H_{33}ClN_4NaO_5$ 539.2037; Found 539.2043.

Ethyl (*E/Z*)-4-(1-(2-(*tert*-butoxycarbonyl)hydrazineylidene)ethyl)-3-(4-fluorophenyl)-1-phenylimidazolidine-4-carboxylate (3Ea): compound **3Ea** was obtained by column chromatography (cycloehexane/ethyl acetate 8:2) in **65%** yield (91.6 mg), 12 h; colorless oil.

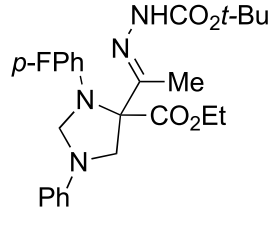

^{1}H NMR (400 MHz, DMSO-*d*$_6$), mixture of *E/Z* isomers (ratio major/minor = 66/34) δ 9.86 – 9.77 (bs, 1H), 7.31 – 6.76 (m, 9H), 4.88 and 4.85 (d, *J* = 4.1 Hz, and *J* = 4.0 Hz, 1H), 4.67 and 4.62 (d, *J* = 4.0 Hz, and *J* = 4.1 Hz, 1H), 4.39 – 4.28 (m, 1H), 4.23 – 4.00 (m, 2H), 3.53 – 3.38 (m, 1H), 1.70 and 1.68 (s, 3H), 1.46 (s, 9H), 1.19 (t, *J* = 7.1 Hz, 3H).

^{13}C{^{1}H} NMR (101 MHz, DMSO-*d*$_6$) for clarity, the signals (some of which overlapping) of both isomers are reported in sequence: δ 170.8, 170.7, 155.7 (d, J_{CF} = 234.9 Hz), 155.6 (d, J_{CF} = 234.9 Hz), 153.2, 153.1, 149.4, 148.7, 146.6, 144.6, 143.5 (d, J_{CF} = 1.7 Hz), 141.2 (d, J_{CF} = 1.7 Hz), 129.5, 128.8, 119.0,

118.0, 116.0 (d, J_{CF} = 22.2 Hz), 115.6 (d, J_{CF} = 7.7 Hz), 115.3 (d, J_{CF} = 15.3 Hz), 115.1, 114.3, 114.2 (d, J_{CF} = 20.8 Hz), 80.1, 80.0, 75.9, 75.8, 68.0, 67.7, 61.5, 61.4, 56.0, 55.3, 28.5, 14.5, 14.4, 13.6, 13.5.

$^{19}F\{^1H\}$ NMR (376 MHz, DMSO-*d*6) δ -126.2, -127.6.

HRMS (ESI-Orbitrap, *m/z*): $[M+Na]^+$ Calcd for $C_{25}H_{31}FN_4NaO_4$ 493.2227; Found 493.2241.

Ethyl-3-(3-bromophenyl)-4-(1-(2-(*tert*-butoxycarbonyl)hydrazineylidene)ethyl)-1-phenylimidazolidine-4-carboxylate (3Fa): compound **3Fa** was obtained by column chromatography (cycloehexane/ethyl acetate 8:2) in **33%** yield (52.5 mg), 12 h; white solid; mp: 133–135 °C.

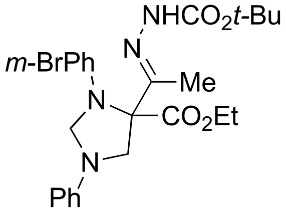

^1H NMR (400 MHz, DMSO-*d*6) δ 9.86 (s, 1H), 7.27 (t, *J* = 8.3, 2H), 7.10 (t, *J* = 8.3 Hz, 1H), 7.05 – 7.01 (m, 1H), 6.93 – 6.78 (m, 5H), 4.89 (d, *J* = 4.3 Hz, 1H), 4.66 (d, *J* = 4.3 Hz, 1H), 4.35 (d, *J* = 9.9 Hz, 1H), 4.33 – 4.00 (m, 2H), 3.46 (d, *J* = 9.9 Hz, 1H), 1.71 (s, 3H), 1.47 (s, 9H), 1.20 (t, *J* = 7.0 Hz, 3H).

$^{13}C\{^1H\}$ NMR (101 MHz, DMSO-*d*6) δ 170.5, 153.1, 148.7, 146.5, 146.0, 130.5, 129.5, 122.3, 120.4, 119.2, 116.5, 114.5, 113.1, 80.0, 76.0, 67.7, 61.6, 55.3, 28.5, 14.4, 13.5.

HRMS (ESI-Orbitrap, *m/z*): $[M+Na]^+$ Calcd for $C_{25}H_{31}BrN_4NaO_4$ 553.1426; Found 553.1423.

7. Access to hydrolyzed imidazolidine 4a.

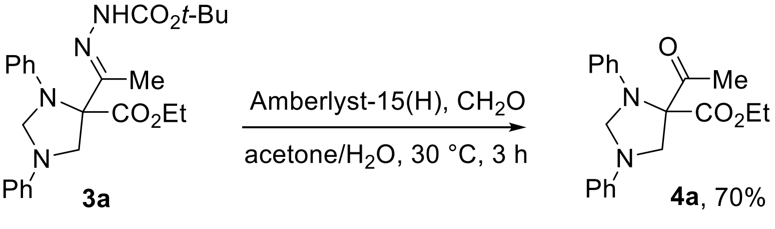

A 10 mL round bottom flask was charged with imidazolidine **3a** (136 mg, 0.3 mmol), paraformaldehyde (18 mg, 0.6 mmol, 2 equiv) and Amberlyst 15H dry (150 mg). Then a solution of acetone/H_2O (9:1, 2

mL) was added and the reaction was stirred at 30°C (oil bath) until starting material **3a** was completely consumed (detected by TLC). The reaction was filtered and washed with DCM and dried with anhydrous Na_2SO_4. The solvent was removed under reduced pressure and the crude was purified through flash column chromatography on silica gel (eluted with Cycl/EtOAc, 9:1) to give the corresponding hydrolyzed product **4a** (74.4 mg, 70% yield).

Ethyl 4-acetyl-1,3-diphenylimidazolidine-4-carboxylate (4a): compound **4a** was obtained by column chromatography (cycloehexane/ethyl acetate 6:4) in **70%** yield (125 mg), 18 h; colorless oil.

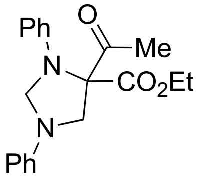

^{1}H NMR (400 MHz, DMSO-*d*$_6$) δ 7.28 (t, *J* = 8.7 Hz, 2H), 7.21 (t, *J* = 8.7 Hz, 2H), 6.91 – 6.74 (m, 4H), 6.67 – 6.58 (m, 2H), 4.93 (d, *J* = 4.1 Hz, 1H), 4.83 (d, *J* = 4.1 Hz, 1H), 4.24 – 4.07 (m, 2H), 4.11 (d, *J* = 10.1 Hz, 1H), 3.99 (d, *J* = 10.1 Hz, 1H), 2.23 (s, 3H), 1.11 (t, *J* = 7.1 Hz, 3H).

^{13}C{^{1}H} NMR (101 MHz, DMSO-*d*$_6$) δ 204.1, 169.5, 146.2, 144.0, 129.6, 129.2, 119.1, 118.5, 114.2, 113.8, 77.5, 67.9, 62.0, 55.6, 26.7, 14.3.

HRMS (ESI-Orbitrap, *m/z*): $[M+H]^+$ Calcd for $C_{20}H_{23}N_2O_3$ 339.1709; Found 339.1701.

8. Mechanistic investigation.

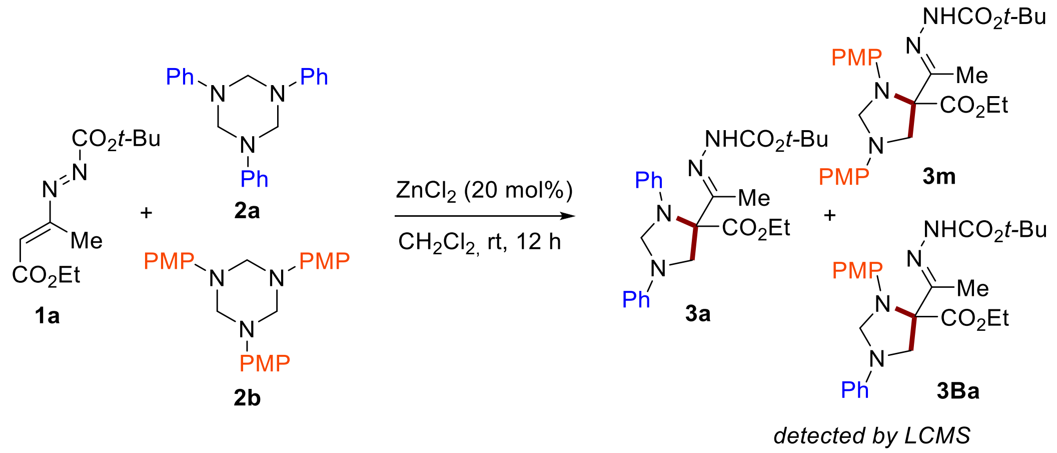

Cross experiment: To a solution of 1,2-diaza-1,3-diene **1a** (0.6 mmol) in CH_2Cl_2 (3 mL), 1,3,5-triphenyl-1,3,5-triazinane **2a** (0.15 mmol) 1,3,5-tri-*p*-methoxy-1,3,5-triazinane (0.15 mmol) and $ZnCl_2$ (0.06 mmol, 8.2 mg) were added. After stirring for 12 h (TLC monitoring) at rt, the reaction mixture was filtered through a short plug of silica gel and concentrated. Then such a crude product was subjected to ^{1}H NMR and LCMS analysis. The result showed the formation of **3a**, **3m** and **3Ba**. See the copy of ^{1}H NMR spectrum of crude below.

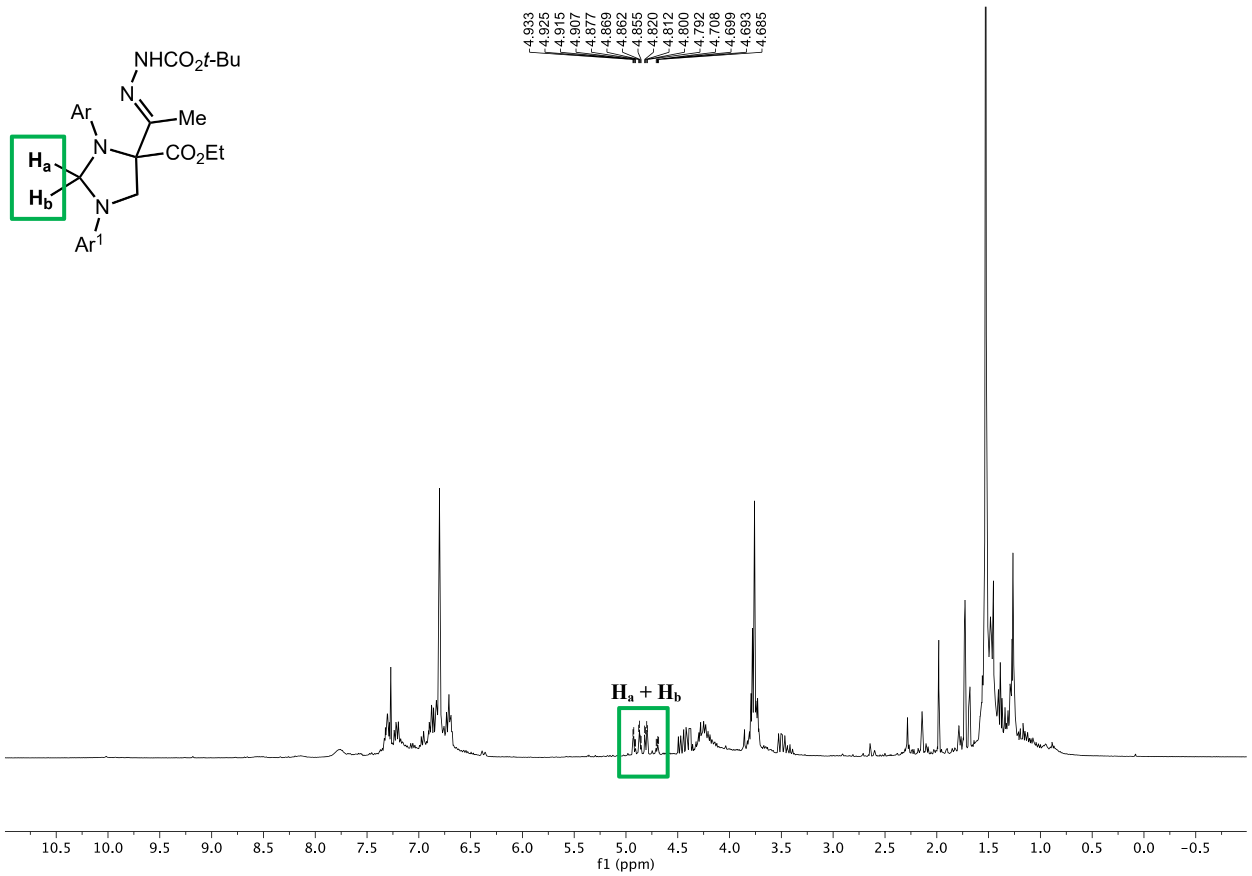

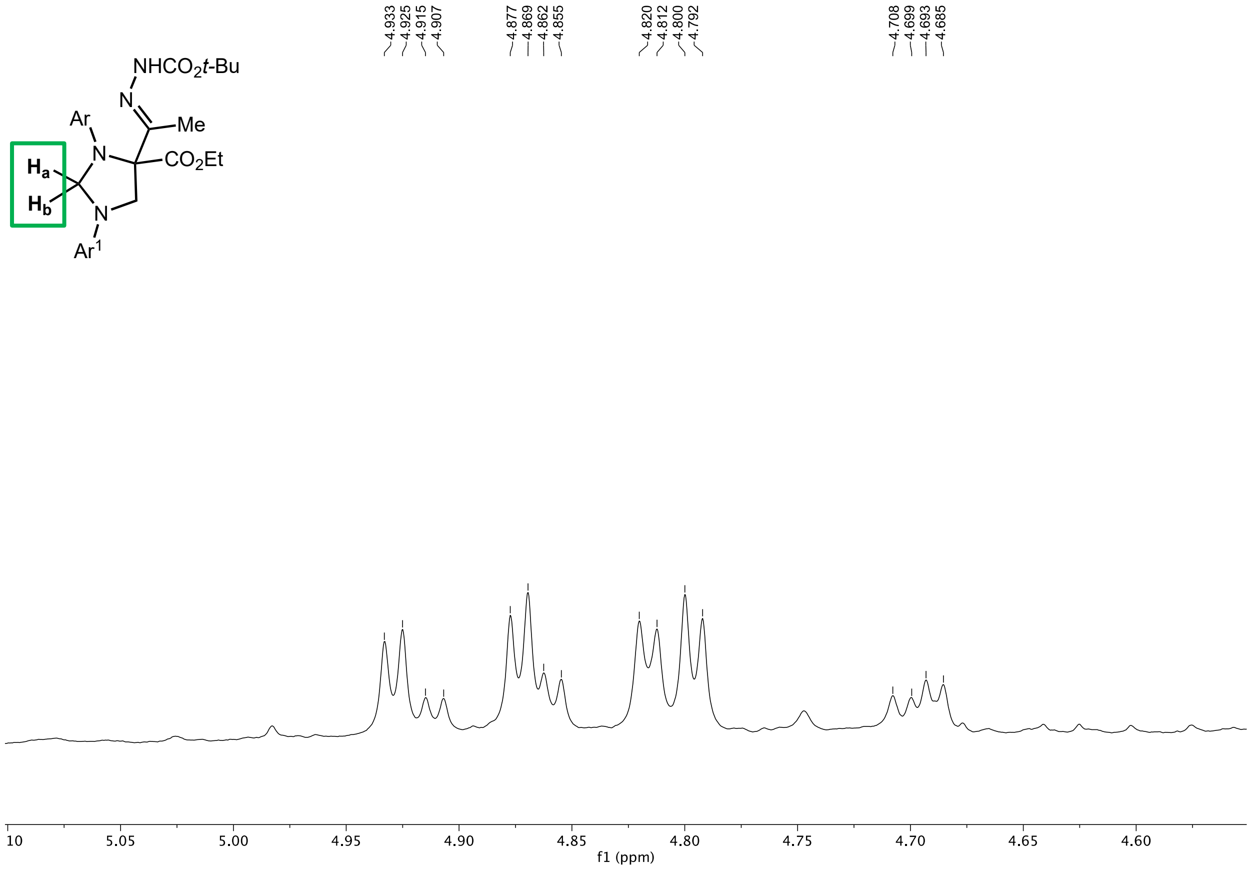

9. References

1 a) Attanasi, O. A.; Filippone, P.; Mei, A; Santeusanio, S. *Synthesis* **1984**, 671−672; b) Attanasi, O. A.; Filippone, P.; Mei, A.; Santeusanio, S. *Synthesis* **1984**, 873−874; c) Preti, L.; Attanasi, O. A.; Caselli, E.; Favi, G.; Ori, C.; Davoli, P.; Felluga, F.; Prati, F. *Eur. J. Org. Chem.* **2010**, 4312−4320.

2 a) Jiang, Q.; Li, A.; Liu, X.; Yu, Y.; Zhu, B.; Cao, H. *J. Org. Chem.* **2022**, *87*, 7056−7063; b) Guranova, N.; Dar'in, D.; Krasavin, M. *Synthesis* **2018**, *50*, 2001–2008.

10. ^{1}H and ^{13}C {^{1}H} NMR

Ethyl-4-(1-(2-(*tert*-butoxycarbonyl)hydrazineylidene)ethyl)-1,3-diphenylimidazolidine-4-carboxylate (3a):

^{1}H NMR (400 MHz, DMSO-d_6) of **3a**:

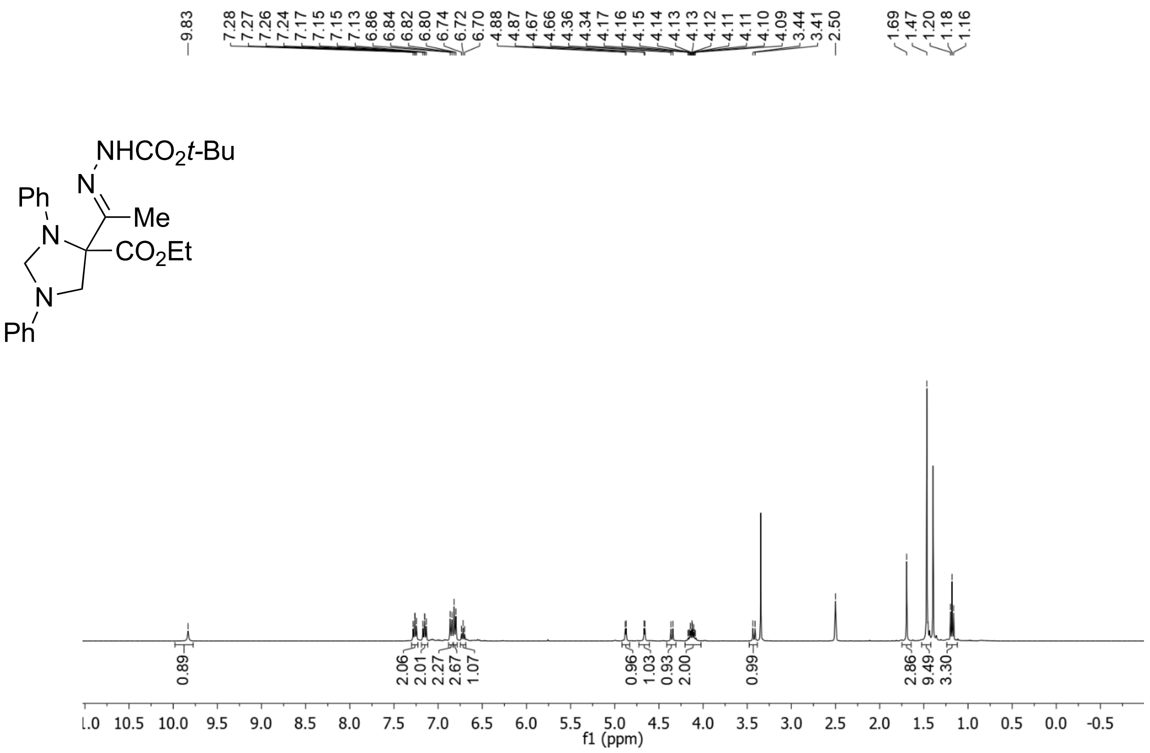

^{13}C {^{1}H} NMR (101 MHz, DMSO-d_6) of **3a**:

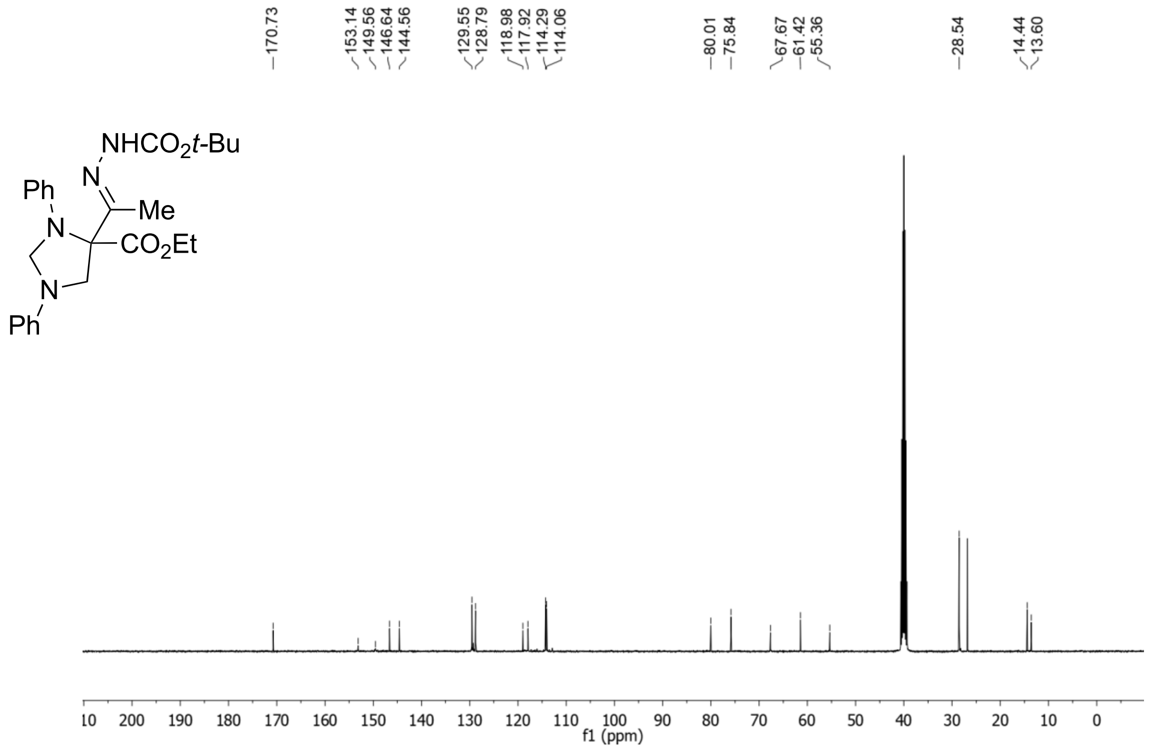

HMQC NMR of 1 (400 MHz, DMSO-d_6) of **3a**:

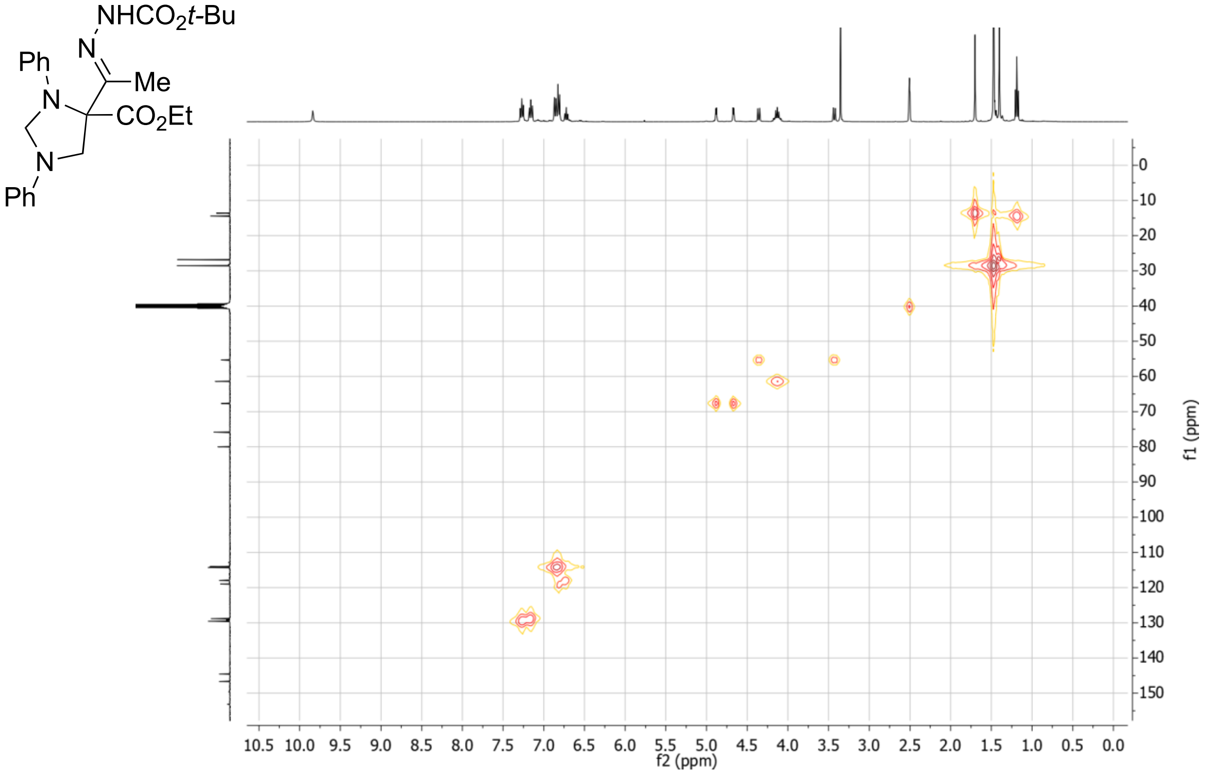

HMBC NMR of 1 (400 MHz, DMSO-d_6) of **3a**:

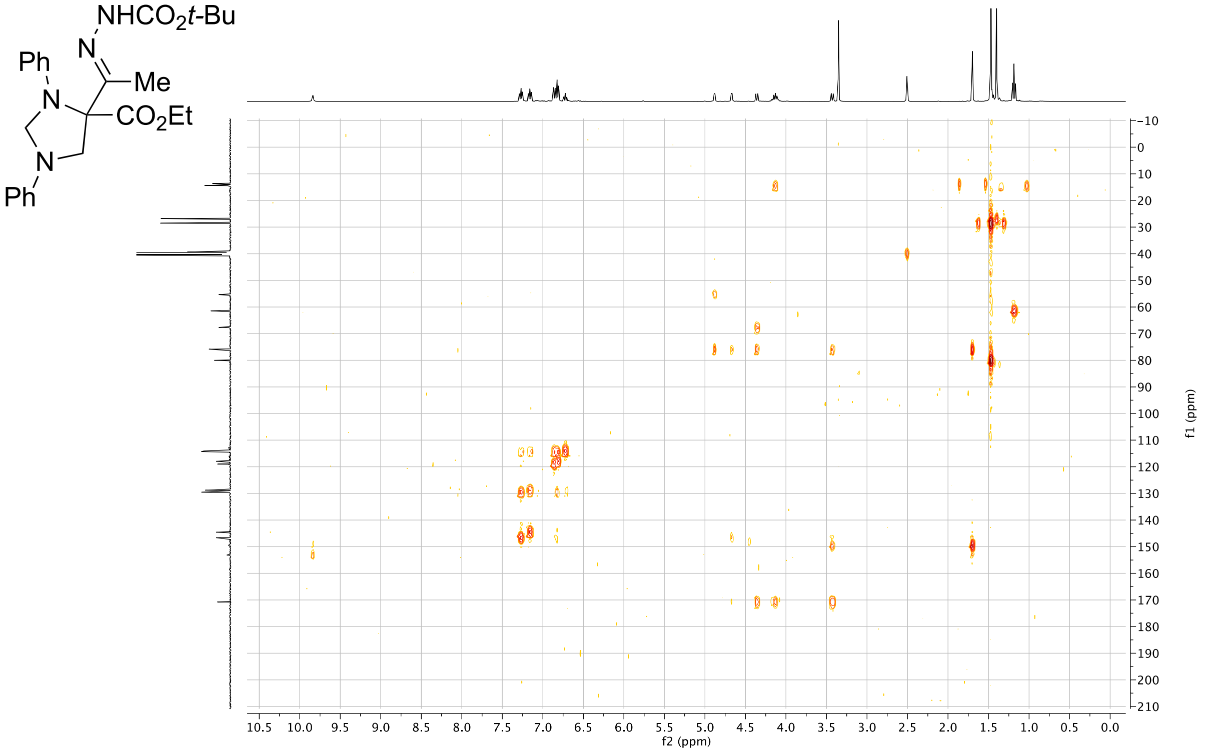

Methyl-4-(1-(2-(*tert*-butoxycarbonyl)hydrazineylidene)ethyl)-1,3-diphenylimidazolidine-4-carboxylate (3b):

^{1}H NMR (400 MHz, $CDCl_3$) of **3b**:

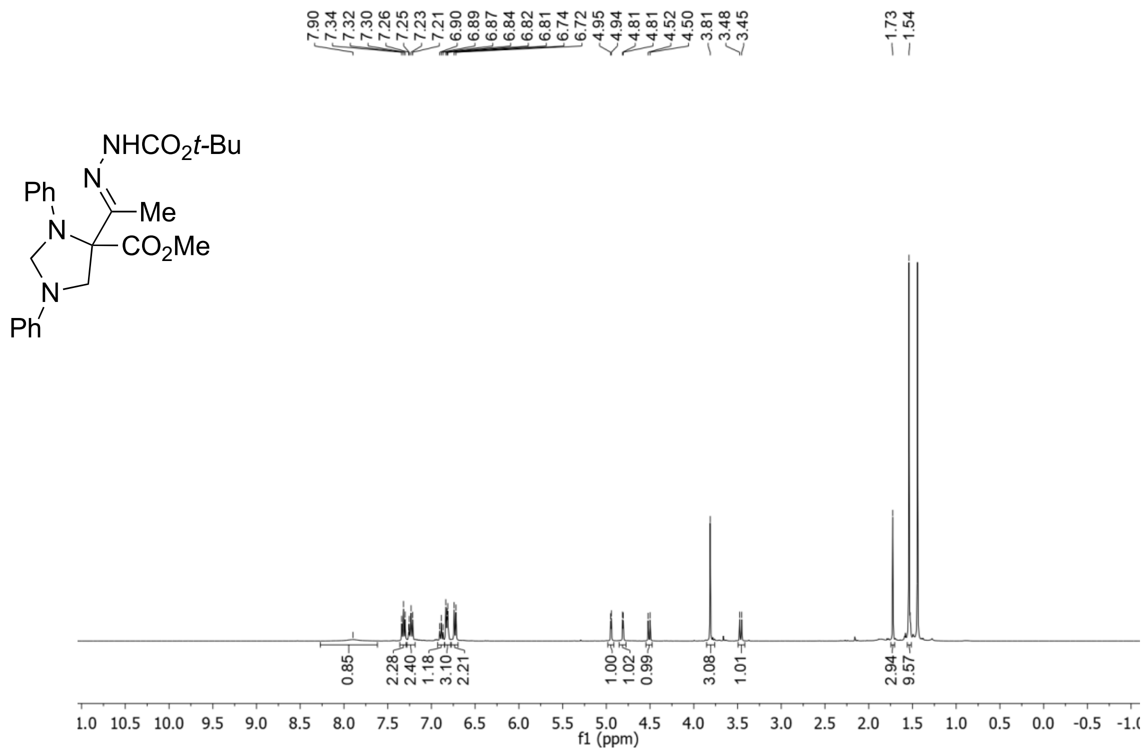

^{13}C {^{1}H} NMR (101 MHz, $CDCl_3$) of **3b**:

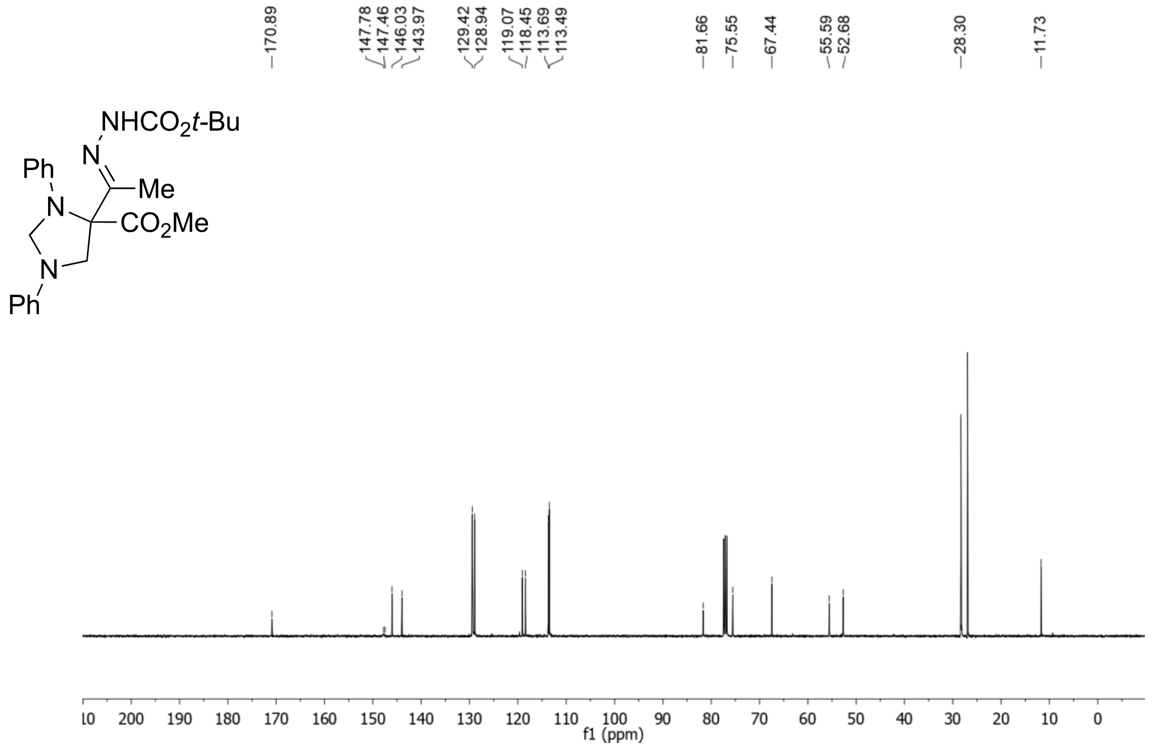

***tert*-Butyl-4-(1-(2-(*tert*-butoxycarbonyl)hydrazineylidene)ethyl)-1,3-diphenylimidazolidine-4-carboxylate (3c):**

^{1}H NMR (400 MHz, DMSO-d_6) of **3c**:

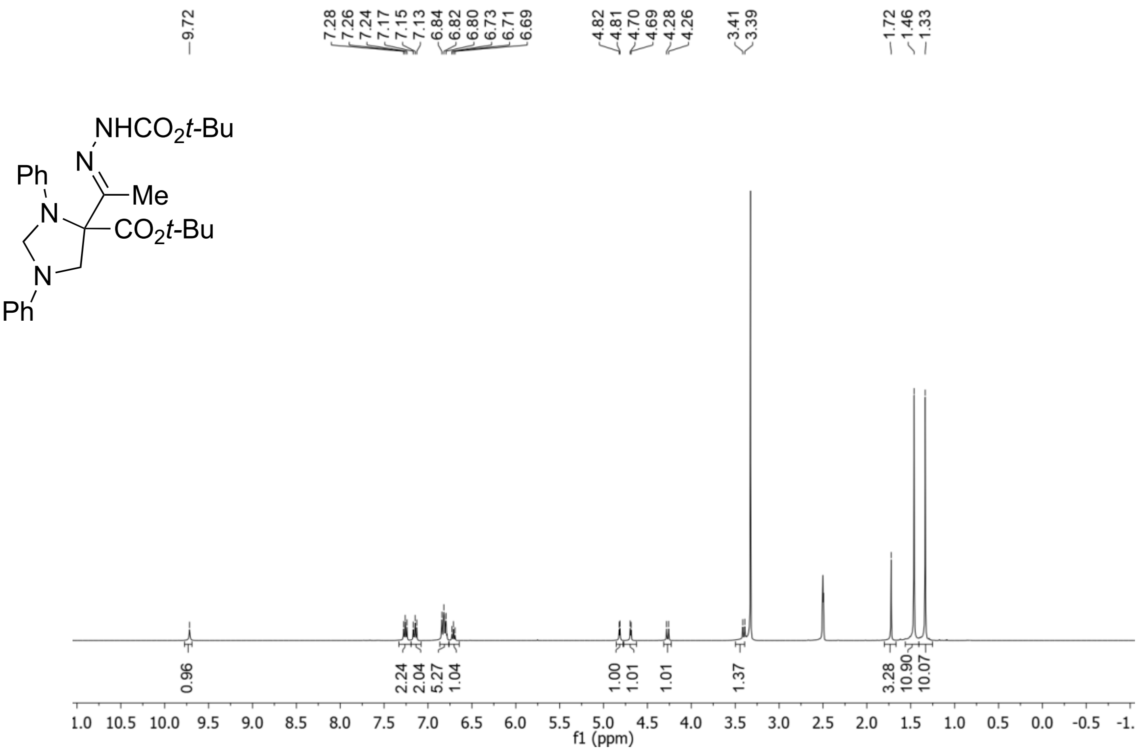

^{13}C {^{1}H} NMR (101 MHz, DMSO-d_6) of **3c**:

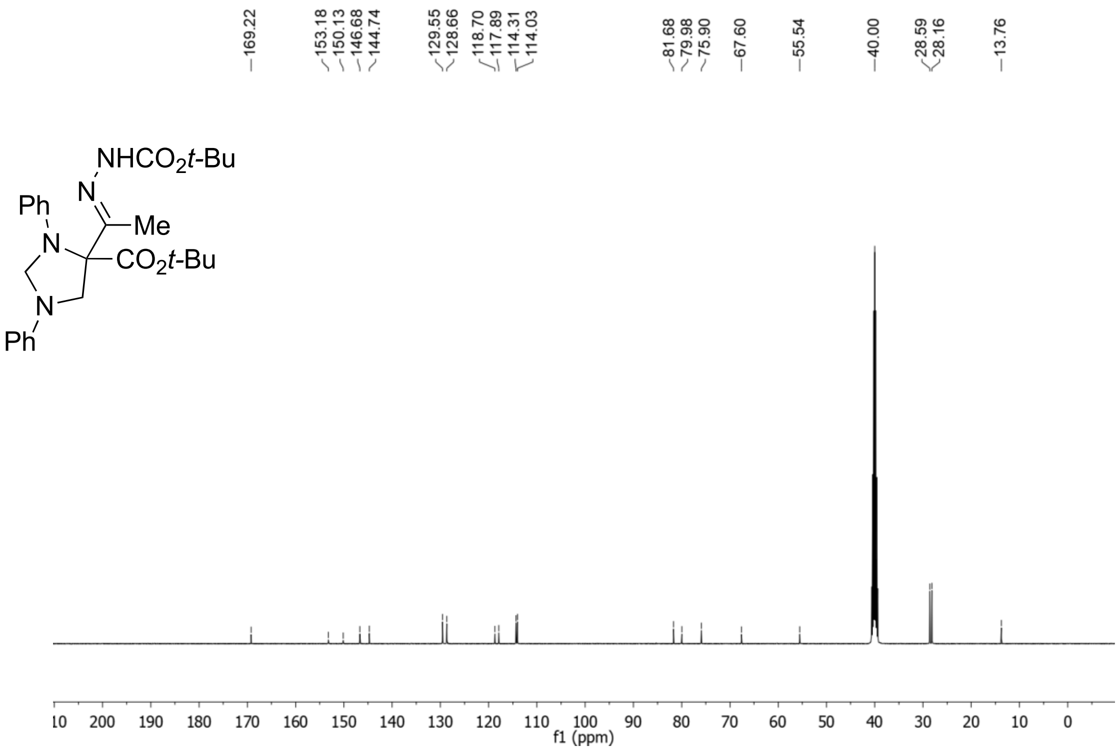

Ethyl-4-(1-(2-(ethoxycarbonyl)hydrazineylidene)ethyl)-1,3-diphenylimidazolidine-4-carboxylate (3d)

^{1}H NMR (400 MHz, $CDCl_3$) of **3d**:

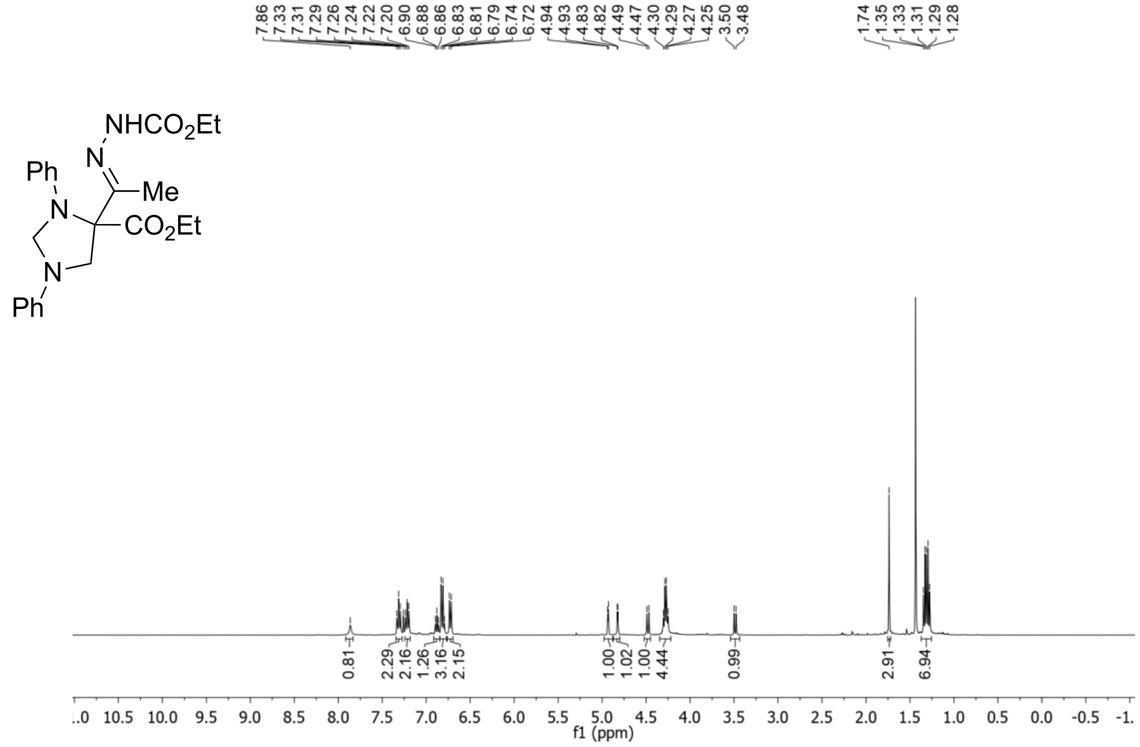

^{13}C {^{1}H} NMR (101 MHz, $CDCl_3$) of **3d**:

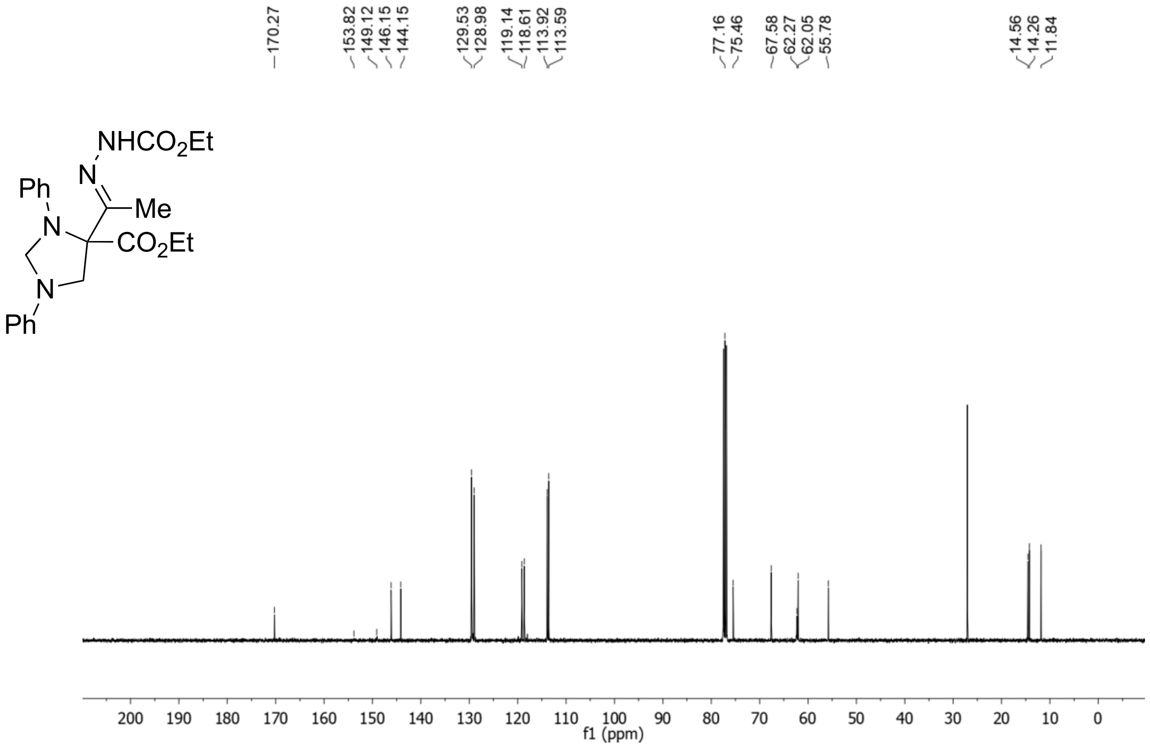

Ethyl-4-(1-(2-(methoxycarbonyl)hydrazineylidene)ethyl)-1,3-diphenylimidazolidine-4-carboxylate (3e)

^{1}H NMR (400 MHz, $CDCl_3$) of **3e**:

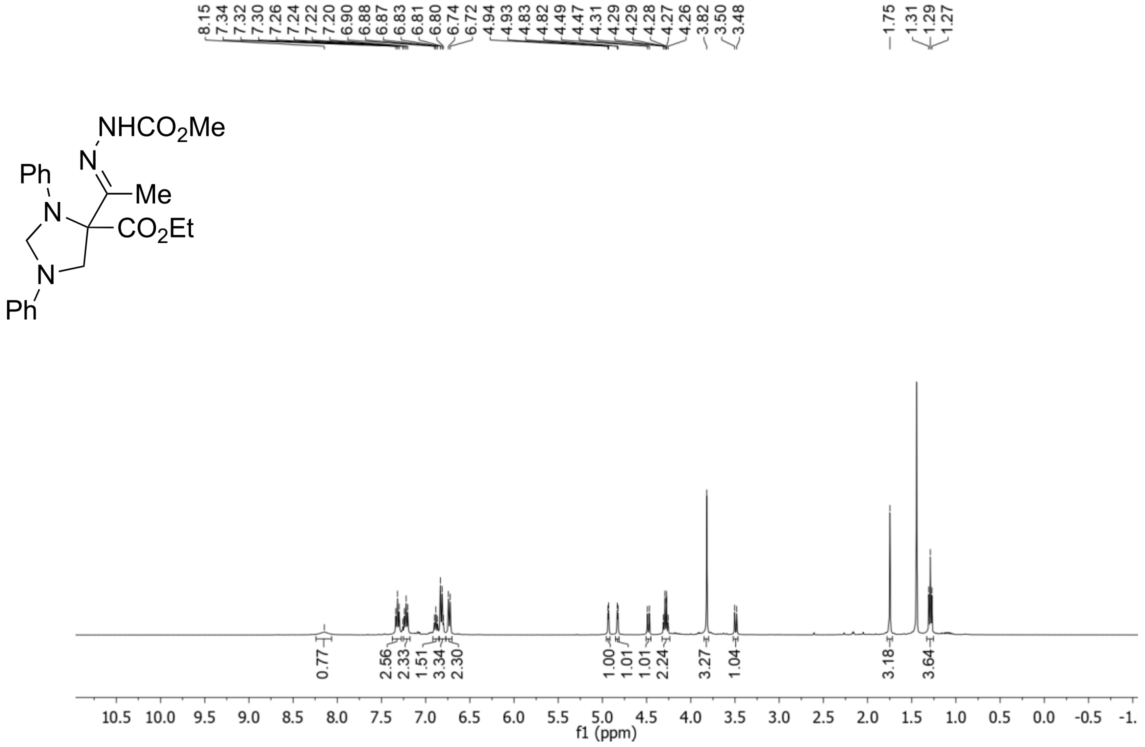

$^{13}C\ \{^{1}H\}$ NMR (101 MHz, $CDCl_3$) of **3e**:

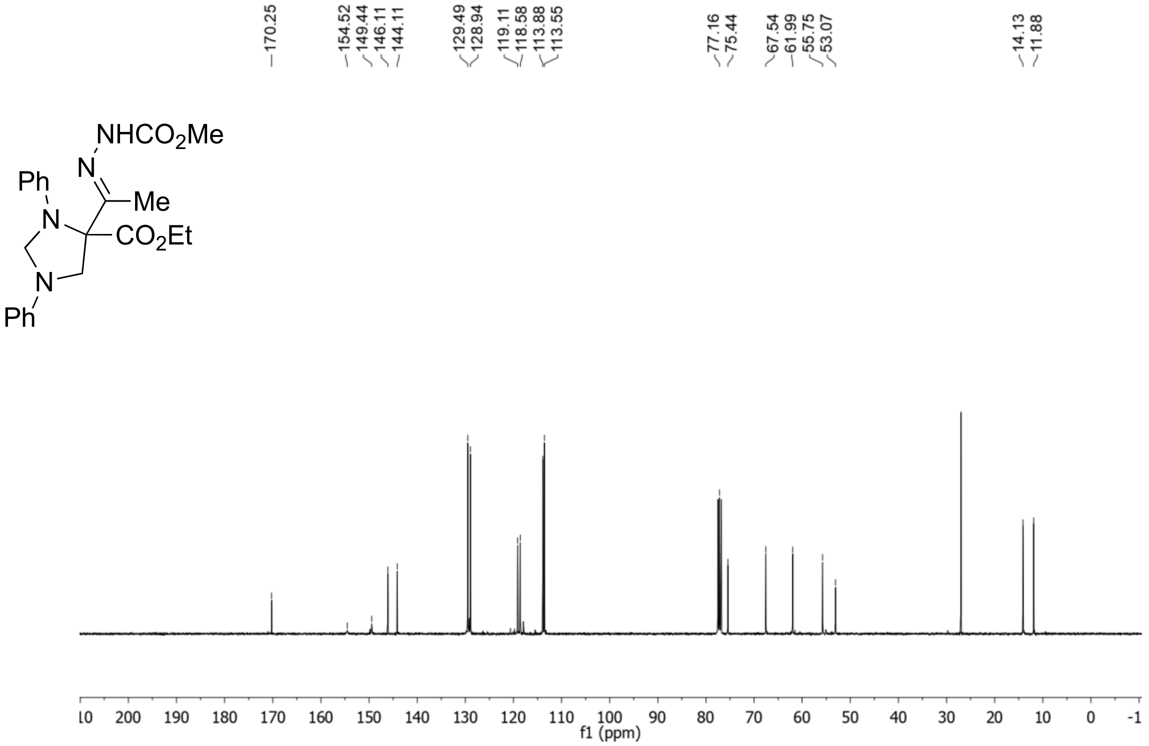

Methyl-4-(1-(2-(methoxycarbonyl)hydrazineylidene)ethyl)-1,3-diphenylimidazolidine-4-carboxylate (3f)

^{1}H NMR (400 MHz, $CDCl_3$) of **3f**:

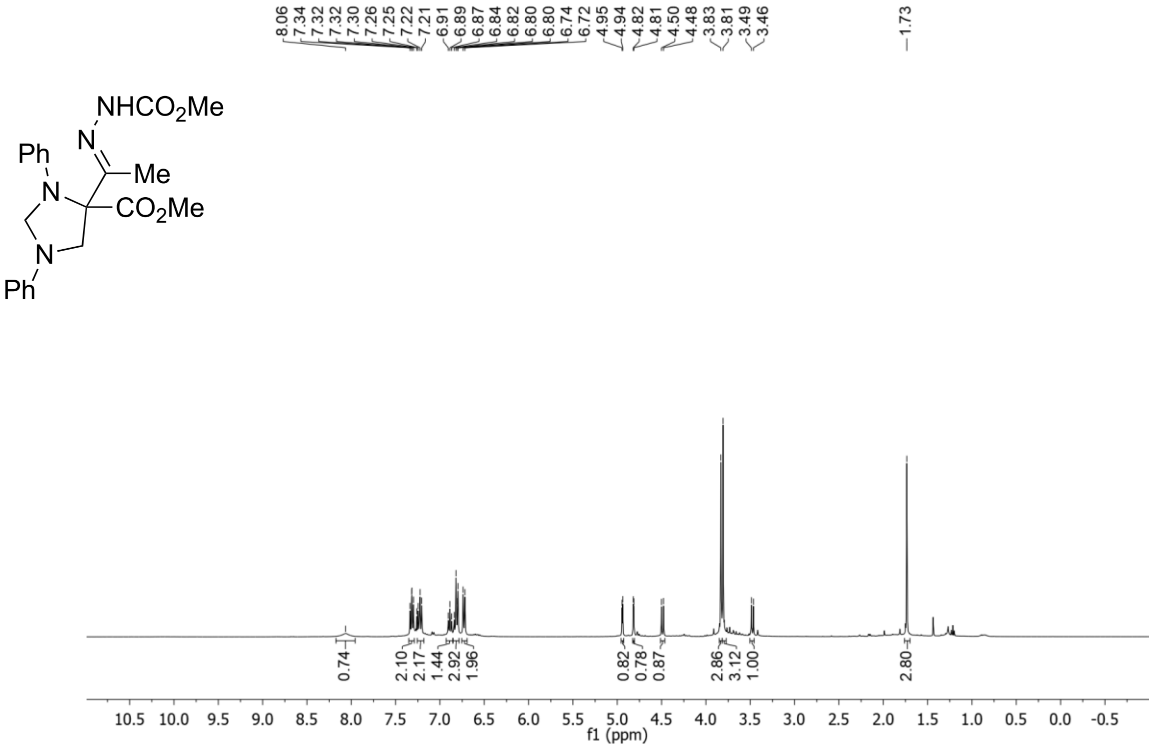

^{13}C {^{1}H} NMR (101 MHz, $CDCl_3$) of **3f**:

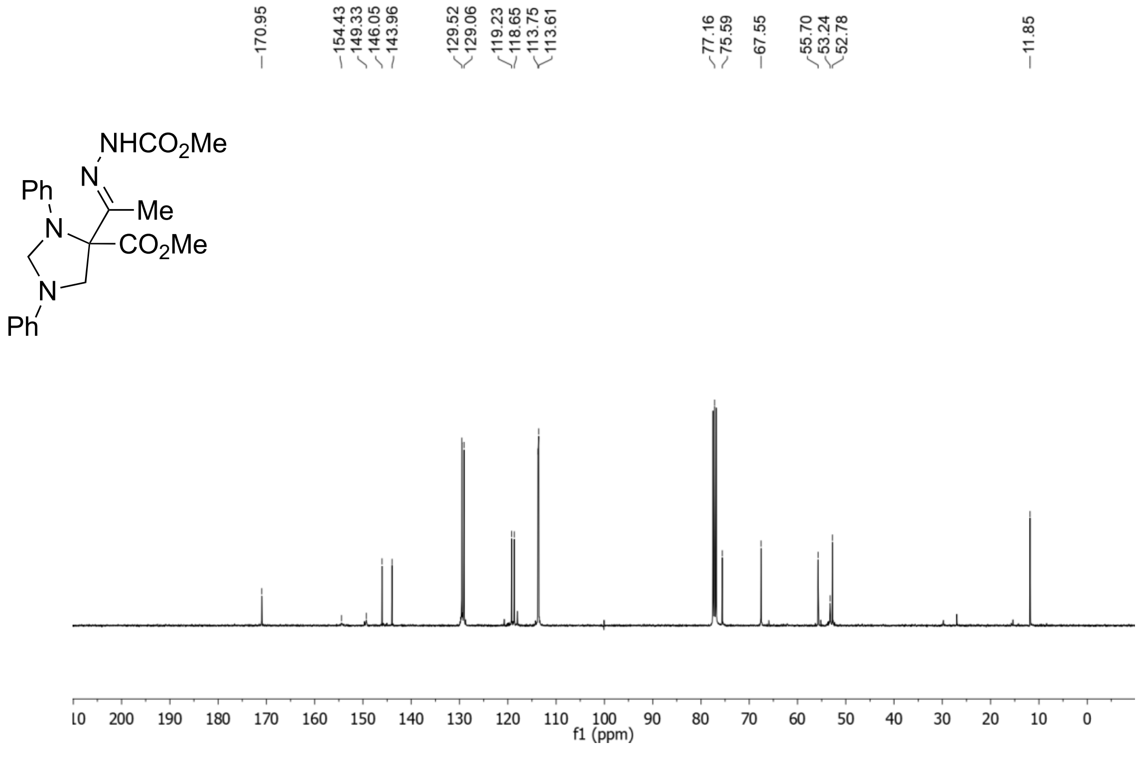

Ethyl-4-(1-(2-(phenoxycarbonyl)hydrazineylidene)ethyl)-1,3-diphenylimidazolidine-4-carboxylate (3g)

^{1}H NMR (400 MHz, $CDCl_3$) of **3g**:

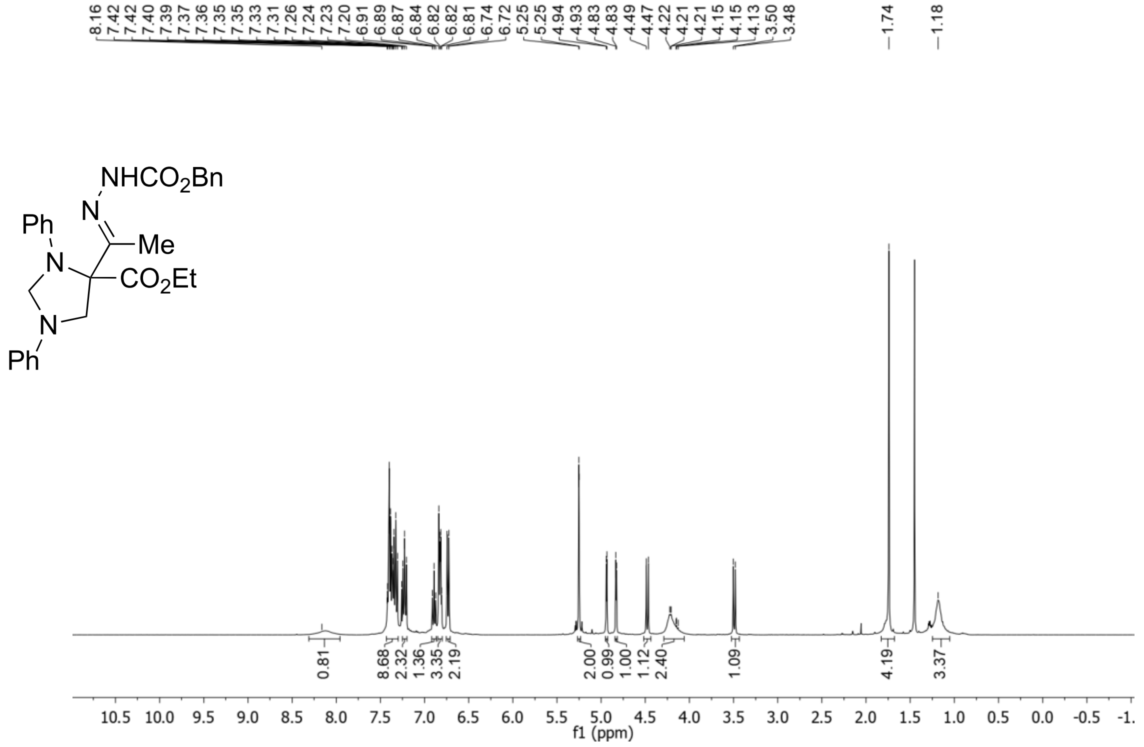

^{13}C {^{1}H} NMR (101 MHz, $CDCl_3$) of **3g**:

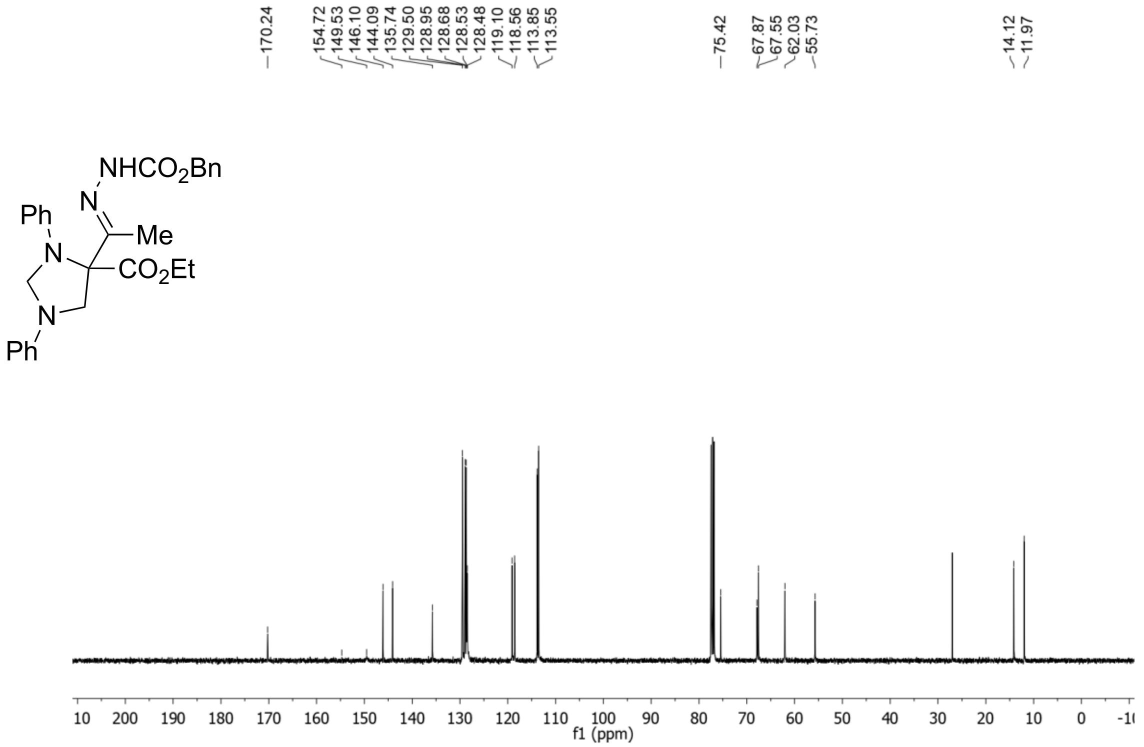

Benzyl-4-(1-(2-(methoxycarbonyl)hydrazineylidene)ethyl)-1,3-diphenylimidazolidine-4-carboxylate (3h)

^{1}H NMR (400 MHz, $CDCl_3$) of **3h**:

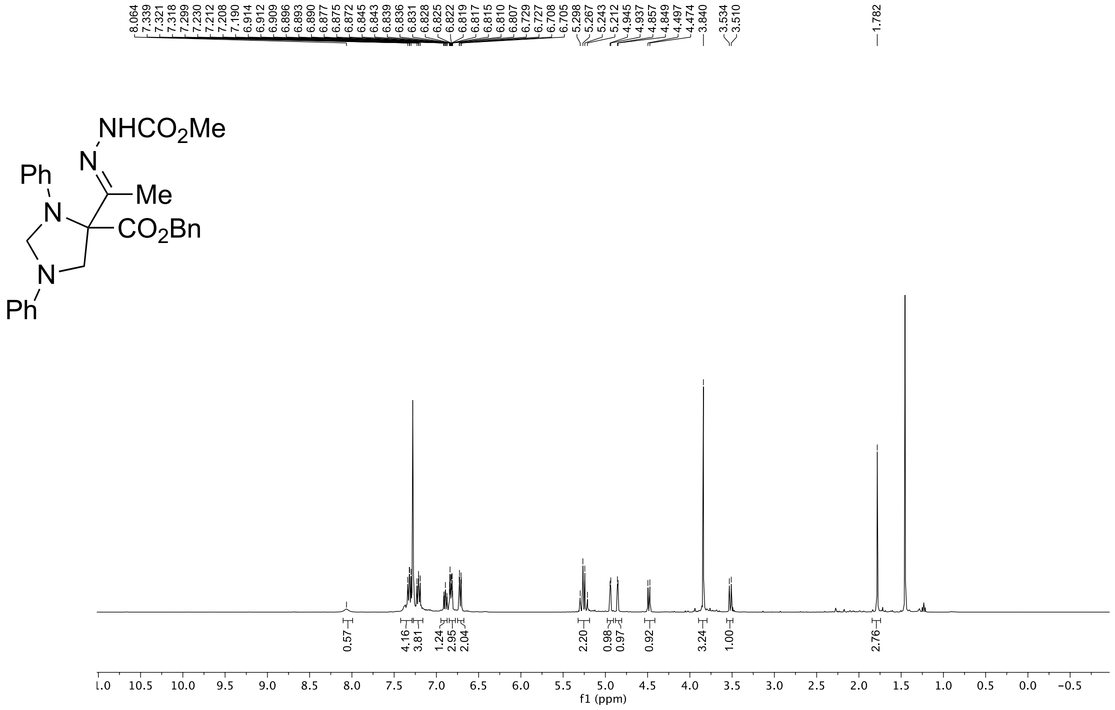

^{13}C {^{1}H} NMR (101 MHz, $CDCl_3$) of **3h**:

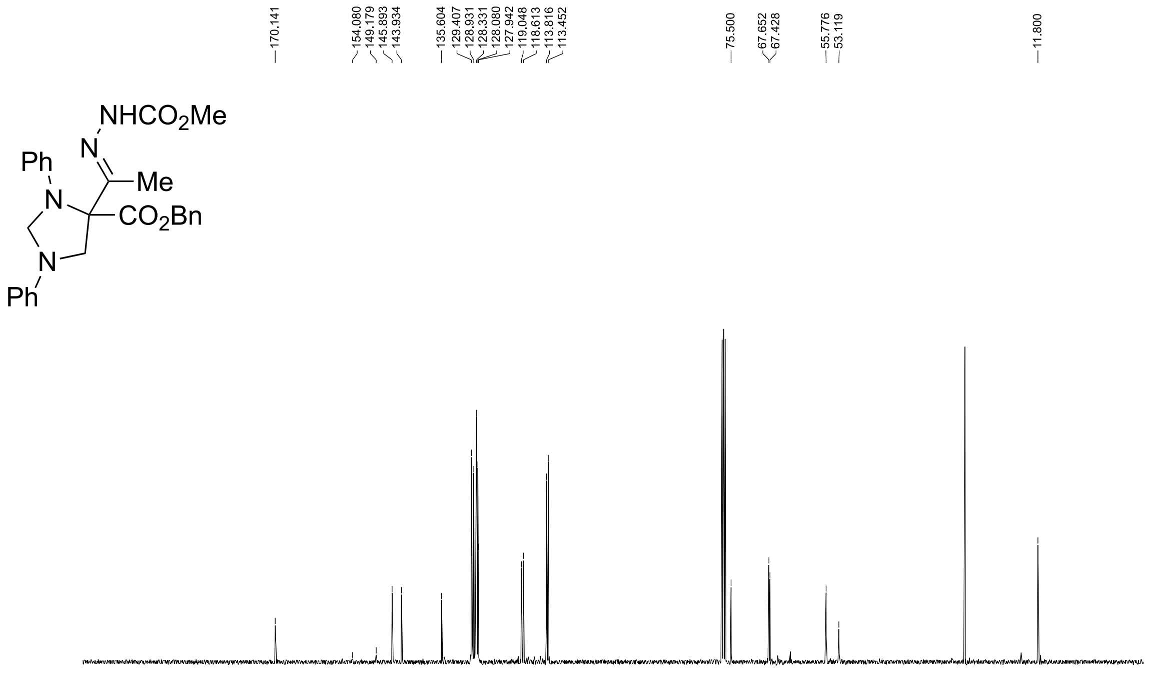

Methyl-4-(1-(2-(*tert*-butoxycarbonyl)hydrazineylidene)butyl)-1,3-diphenylimidazolidine-4-carboxylate (3i)

^{1}H NMR (400 MHz, $CDCl_3$) of **3i**:

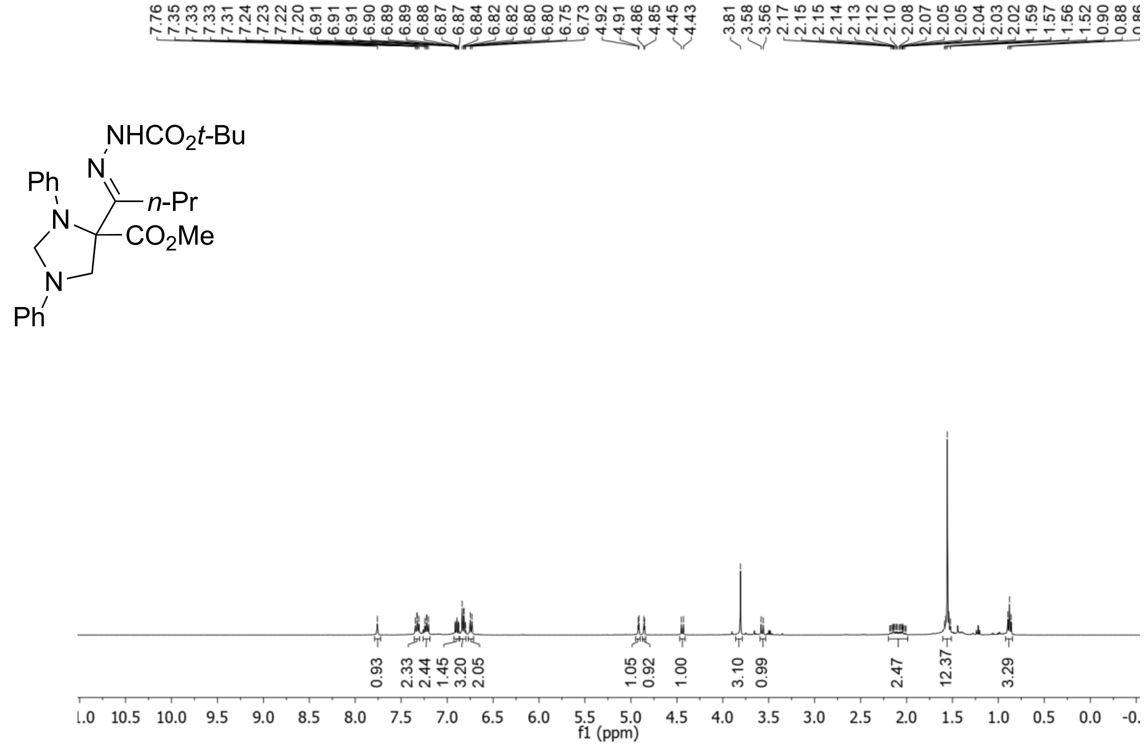

^{13}C {^{1}H} NMR (101 MHz, $CDCl_3$) of **3i**:

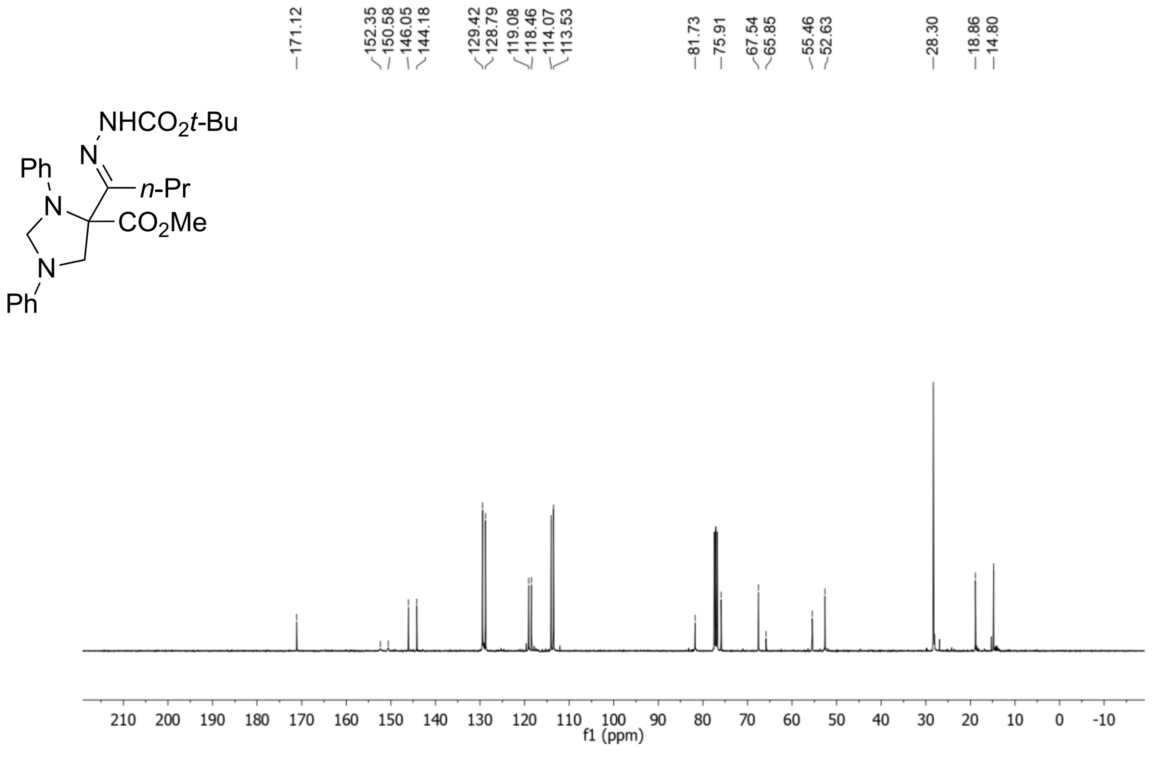

2-Methoxyethyl-4-(1-(2-(ethoxycarbonyl)hydrazineylidene)ethyl)-1,3-diphenylimidazolidine-4-carboxylate (3j)

^{1}H NMR (400 MHz, DMSO-d_6) of **3j**:

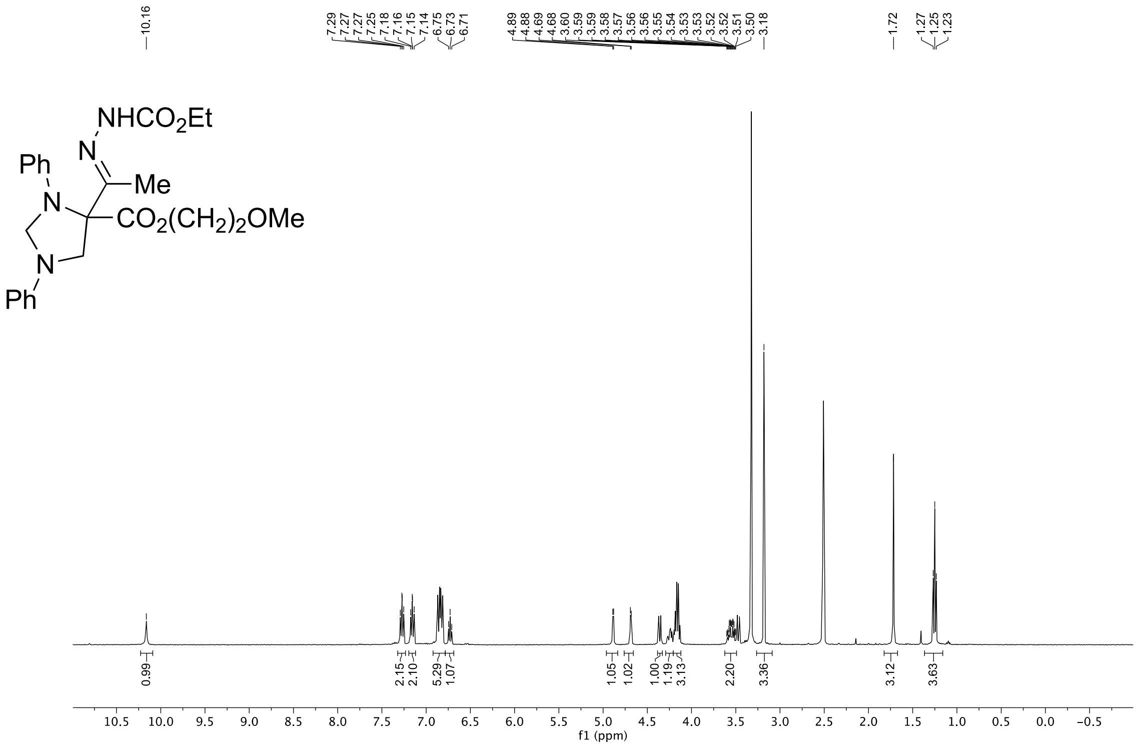

^{13}C {^{1}H} NMR (101 MHz, DMSO-d_6) of **3j**:

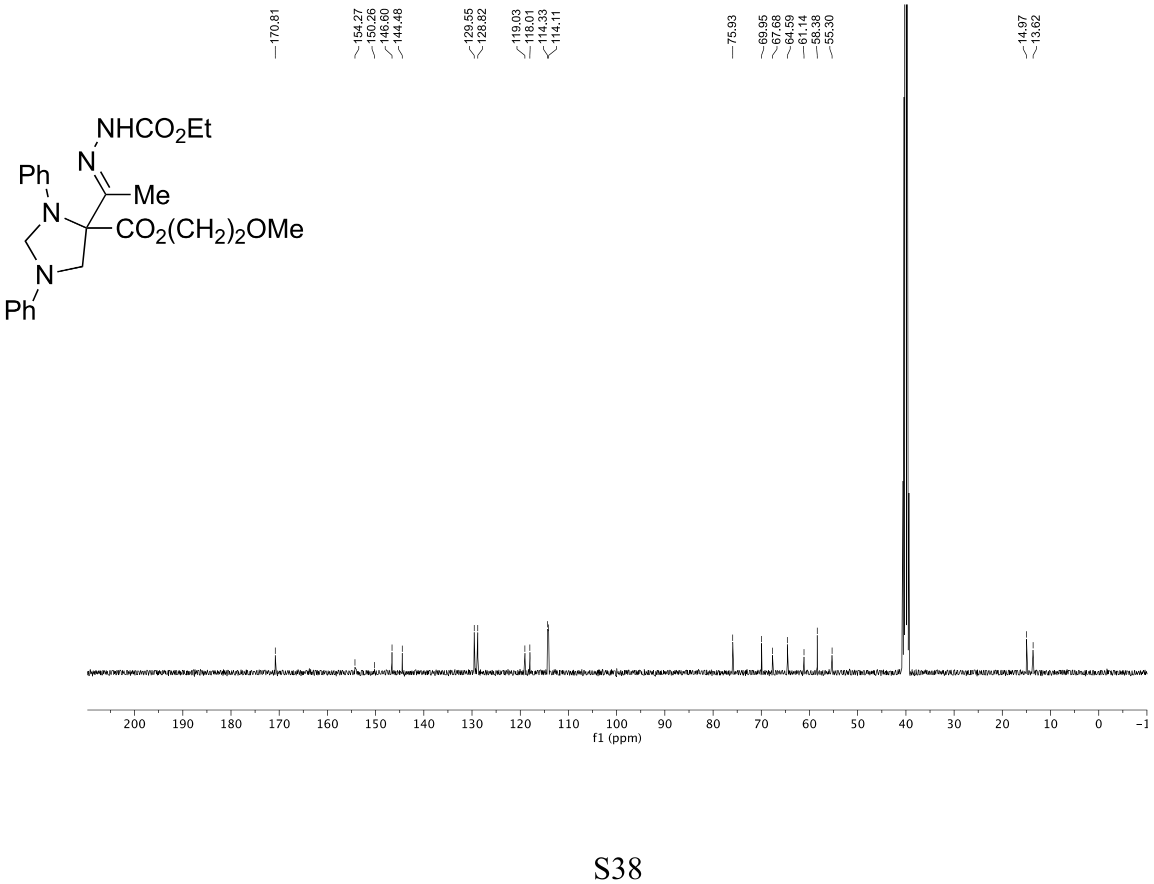

Ethyl-4-(1-(2-carbamoylhydrazineylidene)ethyl)-1,3-diphenylimidazolidine-4-carboxylate (3k)

^{1}H NMR (400 MHz, DMSO-d_6) of **3k**:

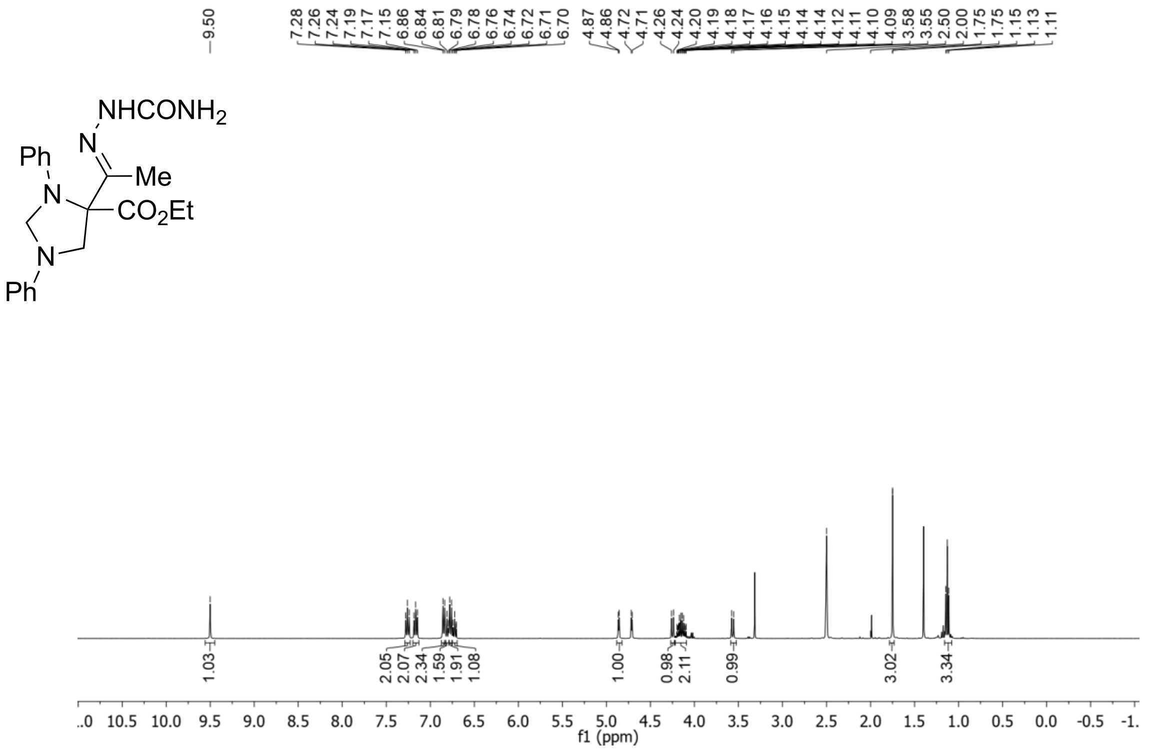

^{13}C {^{1}H} NMR (101 MHz, DMSO-d_6) of **3k**:

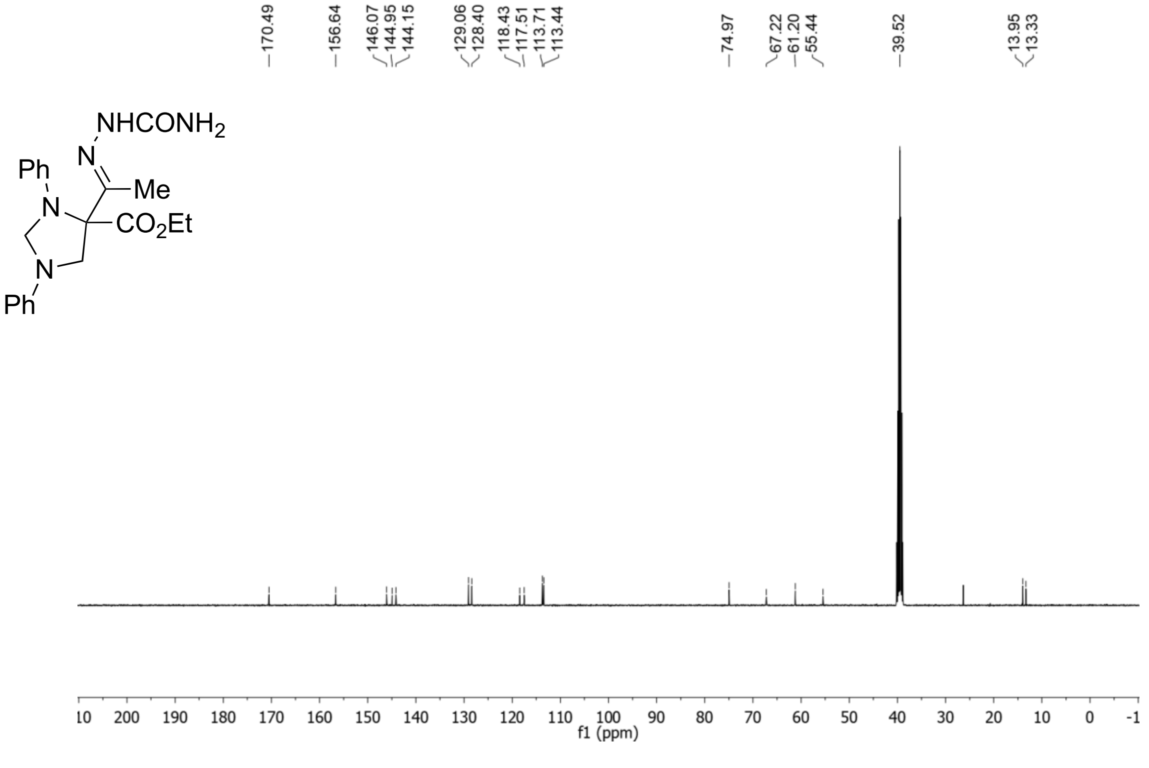

Ethyl-1,3-diphenyl-4-(1-(2-(phenylcarbamoyl)hydrazineylidene)ethyl)imidazolidine-4-carboxylate (3l)

^{1}H NMR (400 MHz, DMSO-d_6) of **3l**:

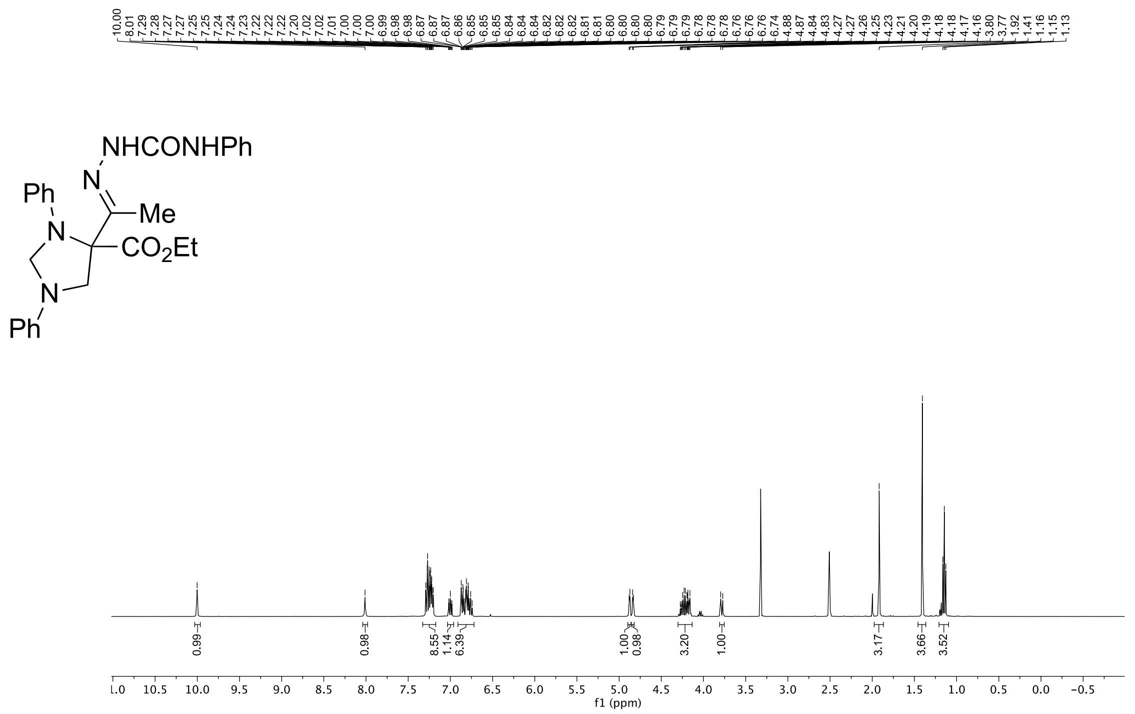

^{13}C {^{1}H} NMR (101 MHz, DMSO-d_6) of **3l**:

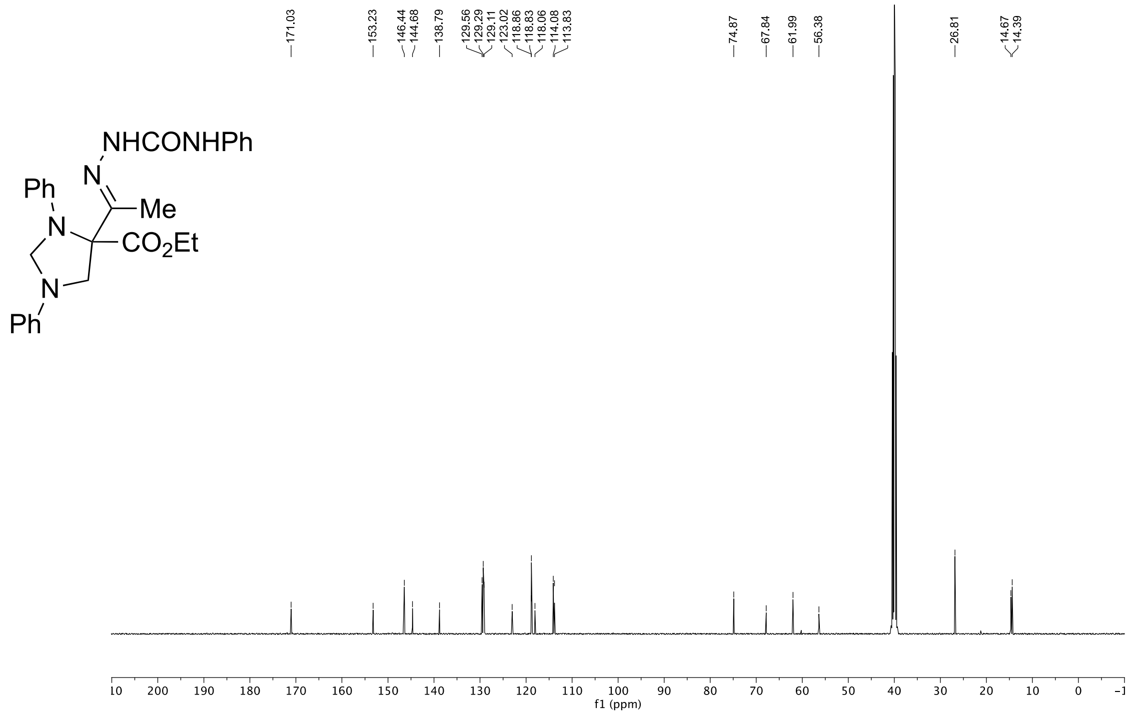

Ethyl-4-(1-(2-(*tert*-butoxycarbonyl)hydrazineylidene)ethyl)-1,3-bis(4-methoxyphenyl) imidazolidine-4-carboxylate (3m)

^{1}H NMR (400 MHz, $CDCl_3$) of **3m**:

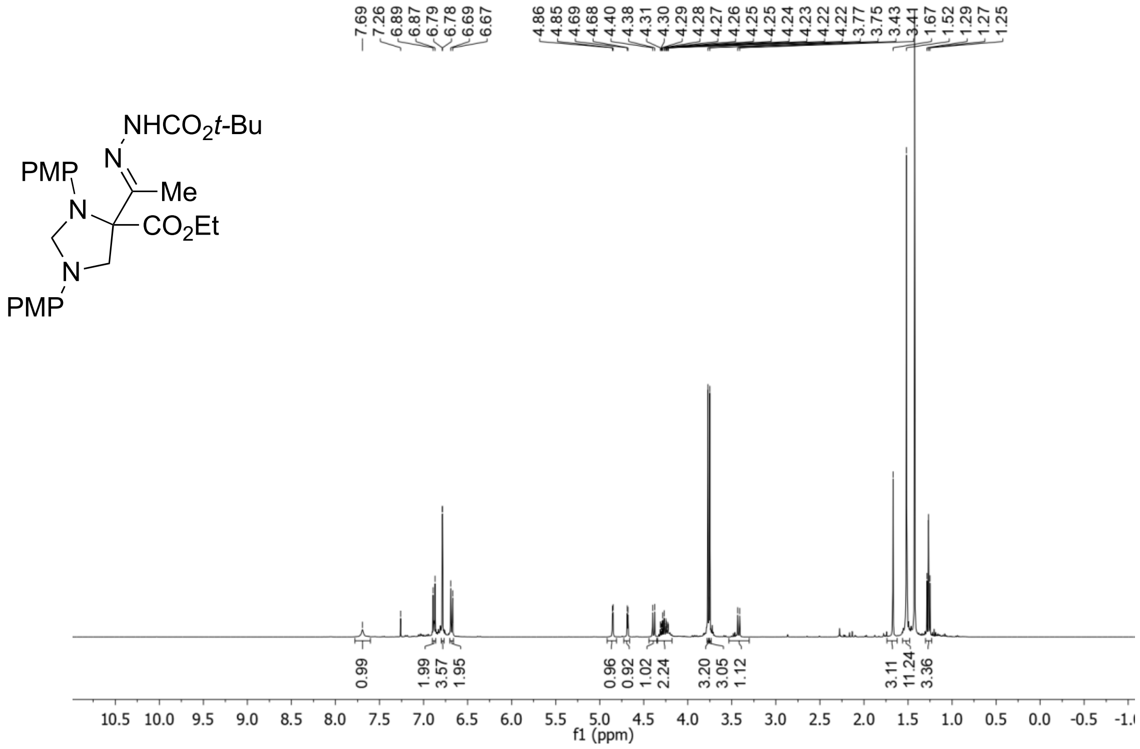

^{13}C {^{1}H} NMR (101 MHz, $CDCl_3$) of **3m**:

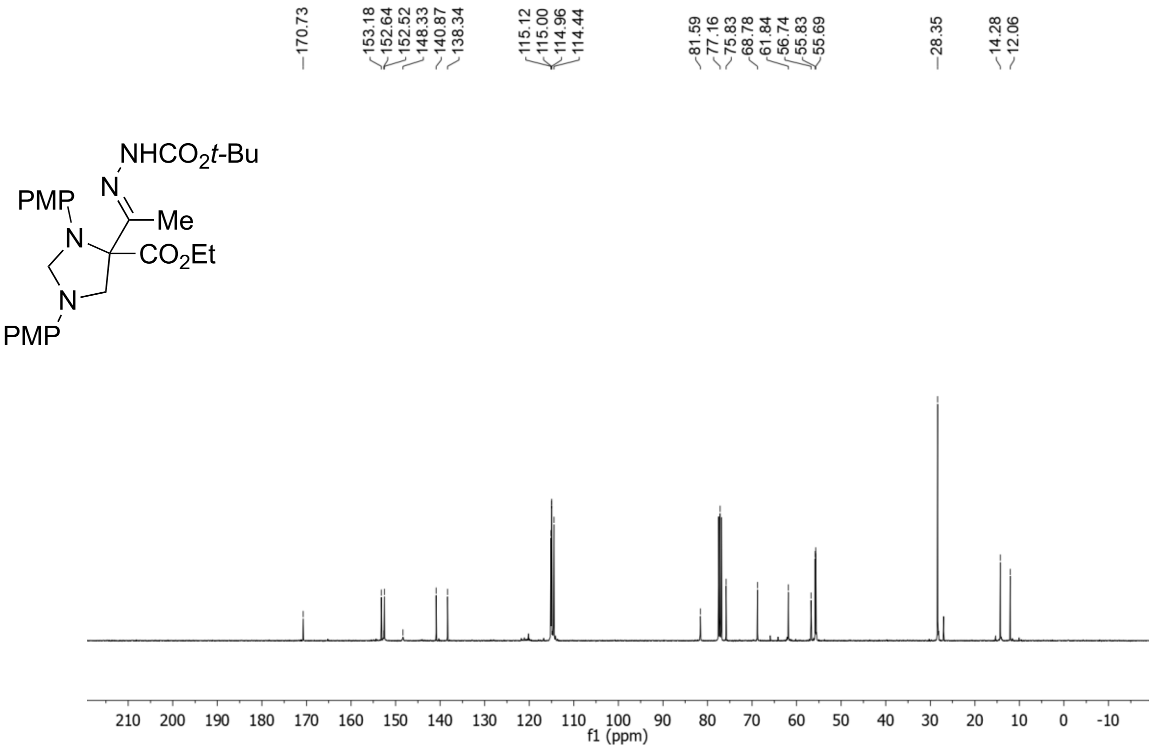

Methyl-4-(1-(2-(*tert*-butoxycarbonyl)hydrazineylidene)ethyl)-1,3-bis(4-methoxyphenyl) imidazolidine-4-carboxylate (3n):

^{1}H NMR (400 MHz, DMSO-d_6) of **3n**:

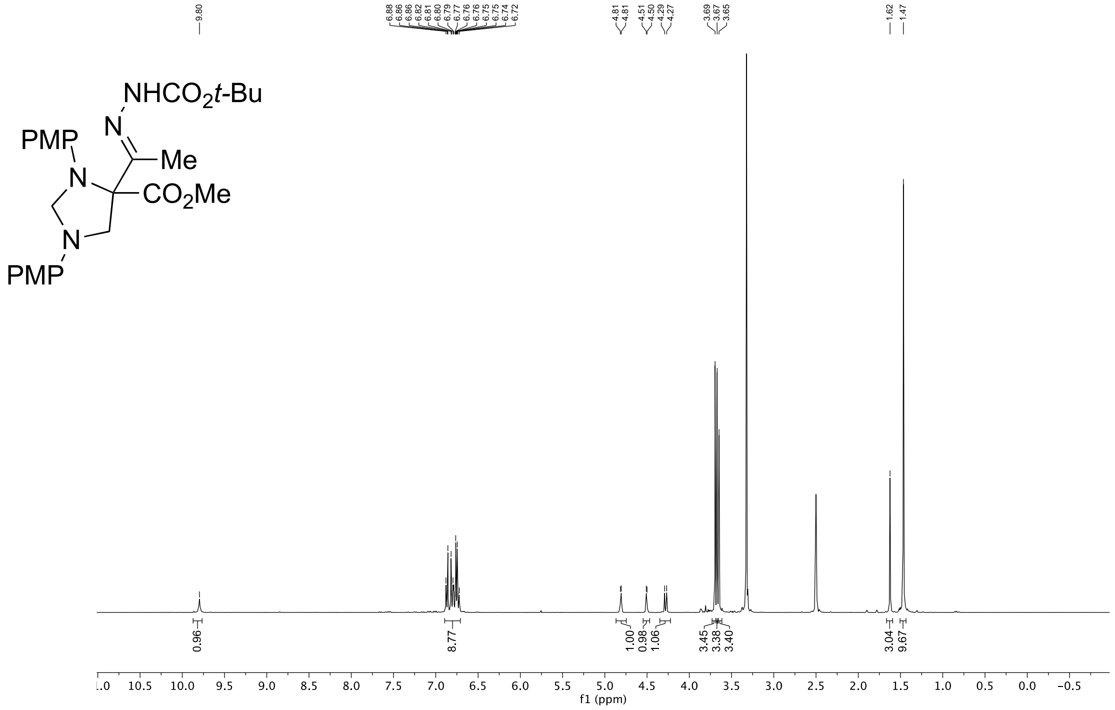

^{13}C {^{1}H} NMR (101 MHz, DMSO-d_6) of **3n**:

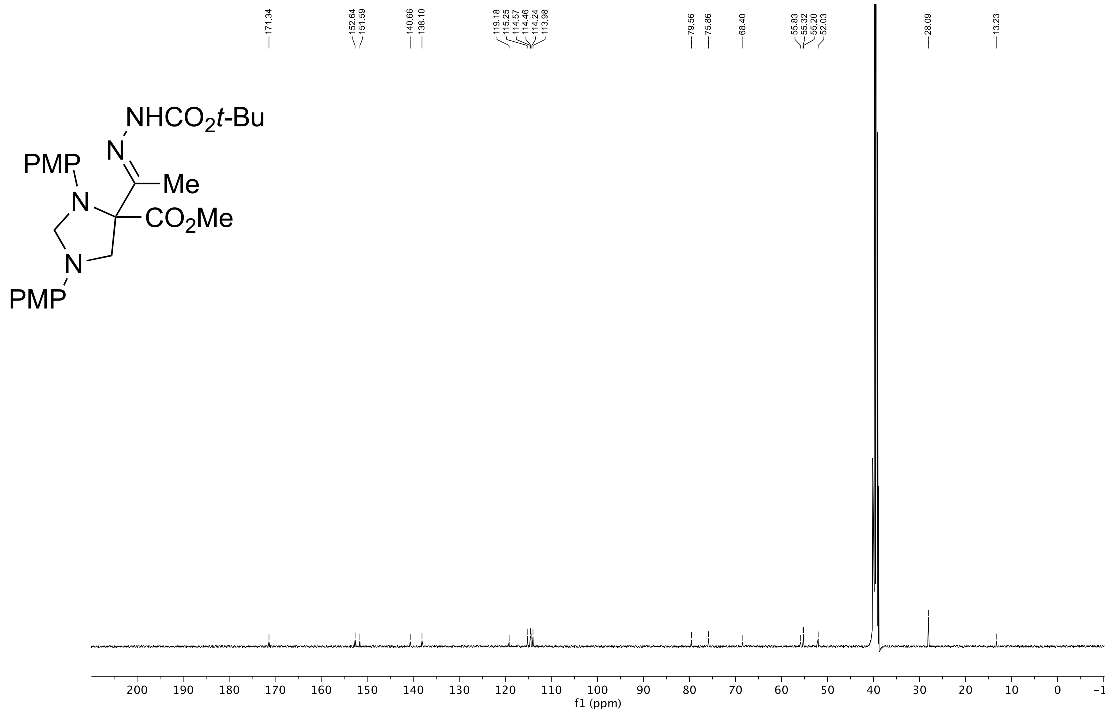

Ethyl-4-(1-(2-(ethoxycarbonyl)hydrazineylidene)ethyl)-1,3-bis(4-methoxyphenyl)imidazolidine-4-carboxylate (3o):

^{1}H NMR (400 MHz, DMSO-d_6) of **3o**:

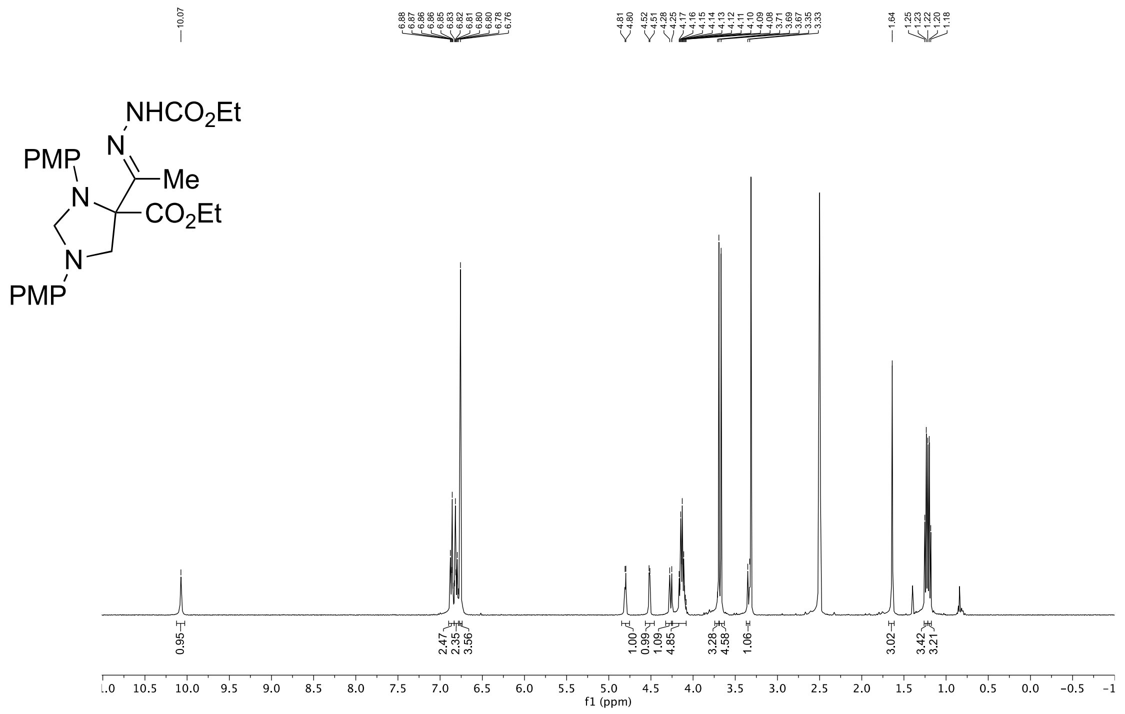

^{13}C {^{1}H} NMR (101 MHz, DMSO-d_6) of **3o**:

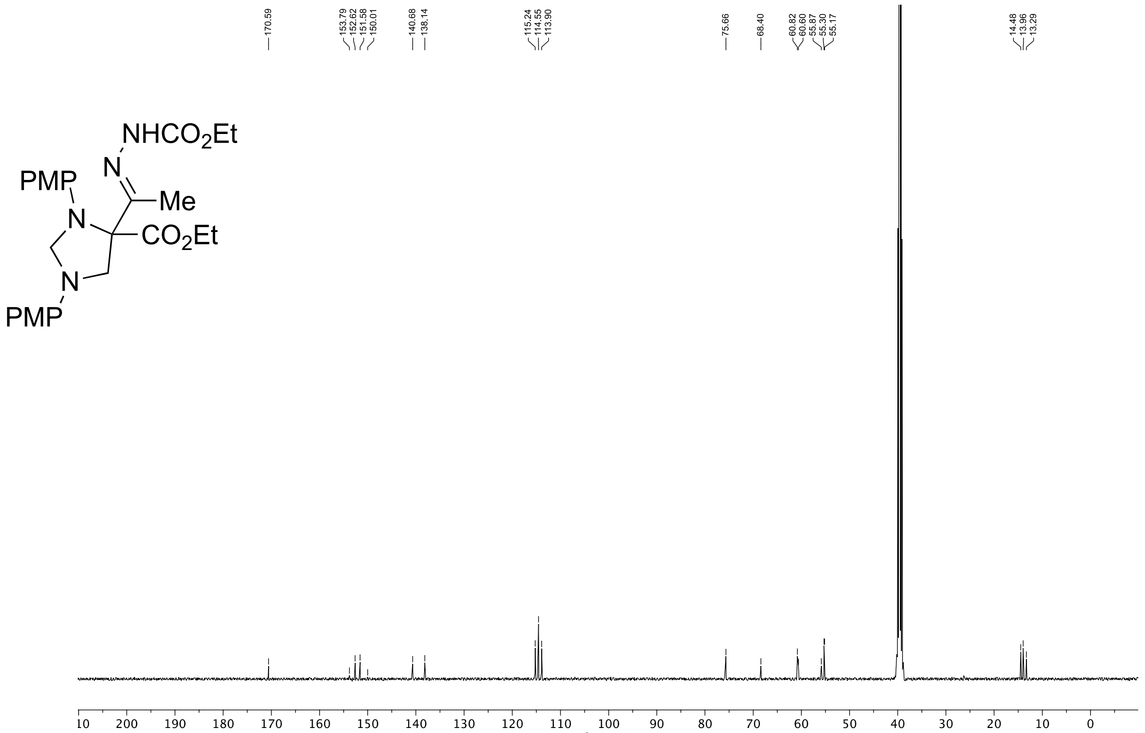

Ethyl-4-(1-(2-(*tert*-butoxycarbonyl)hydrazineylidene)ethyl)-1,3-di-*p*-tolylimidazolidine-4-carboxylate (3p)

^{1}H NMR (400 MHz, $CDCl_3$) of **3p**:

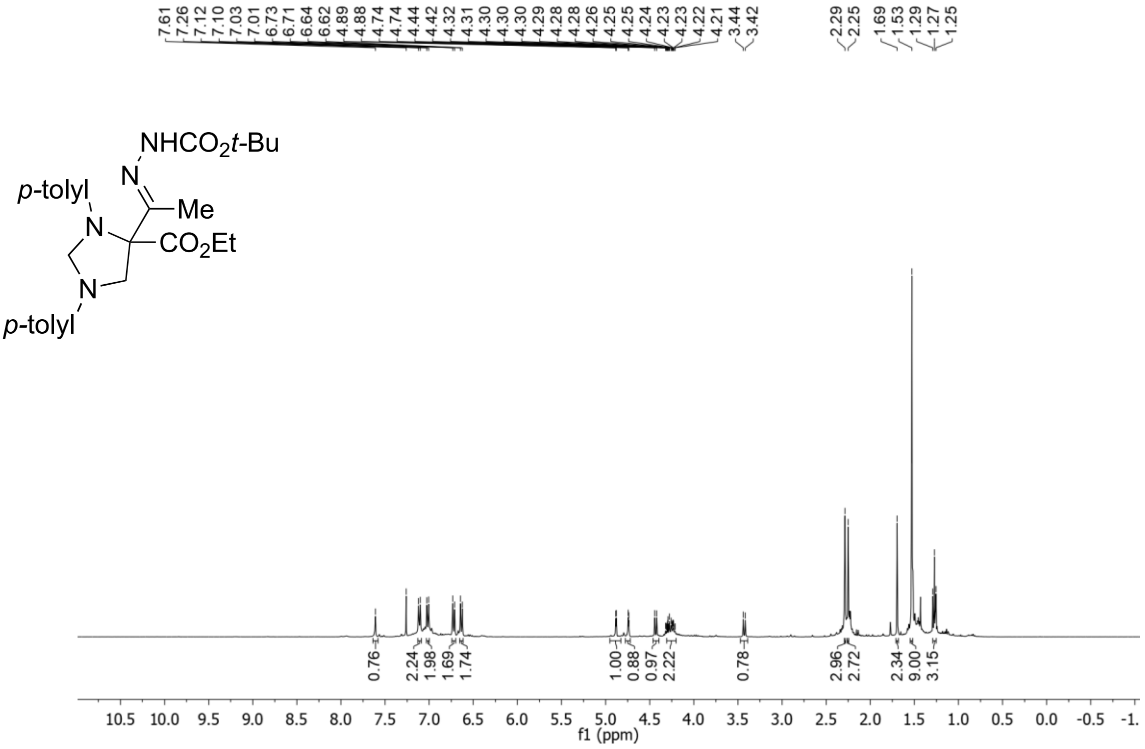

^{13}C {^{1}H} NMR (101 MHz, $CDCl_3$) of **3p**:

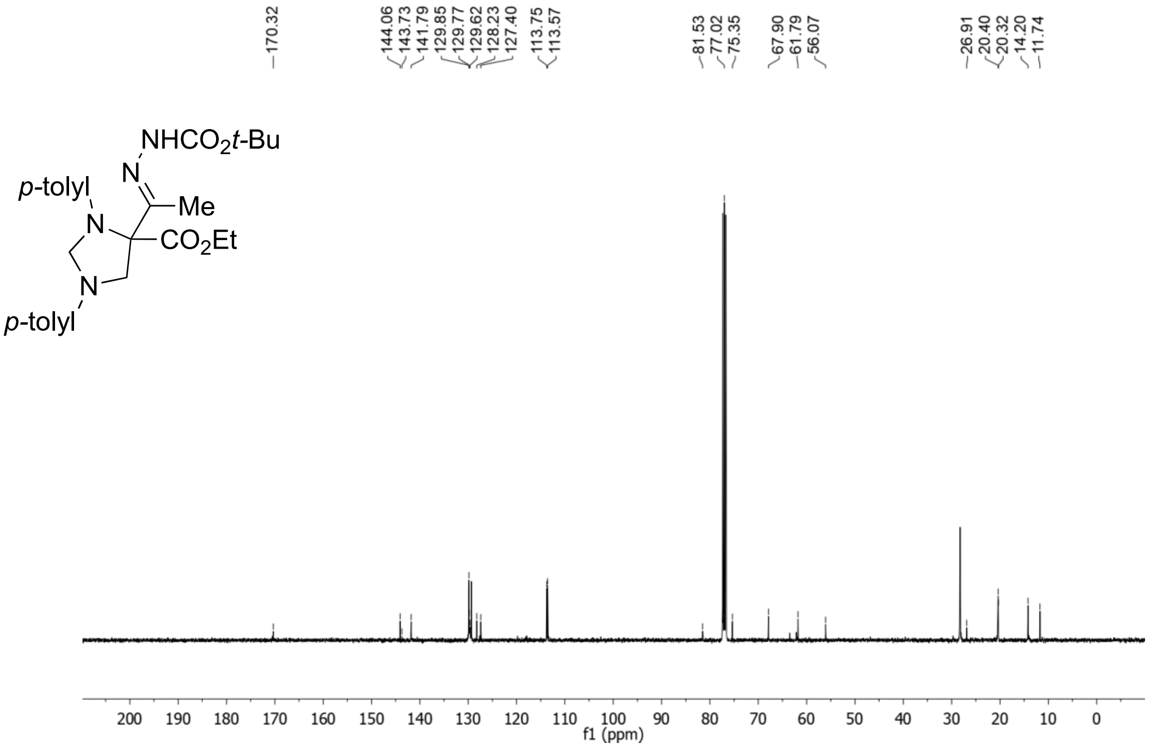

Ethyl-4-(1-(2-(*tert*-butoxycarbonyl)hydrazineylidene)ethyl)-1,3-bis(4 chlorophenyl) imidazolidine-4-carboxylate (3q)

^{1}H NMR (400 MHz, $CDCl_3$) of **3q**:

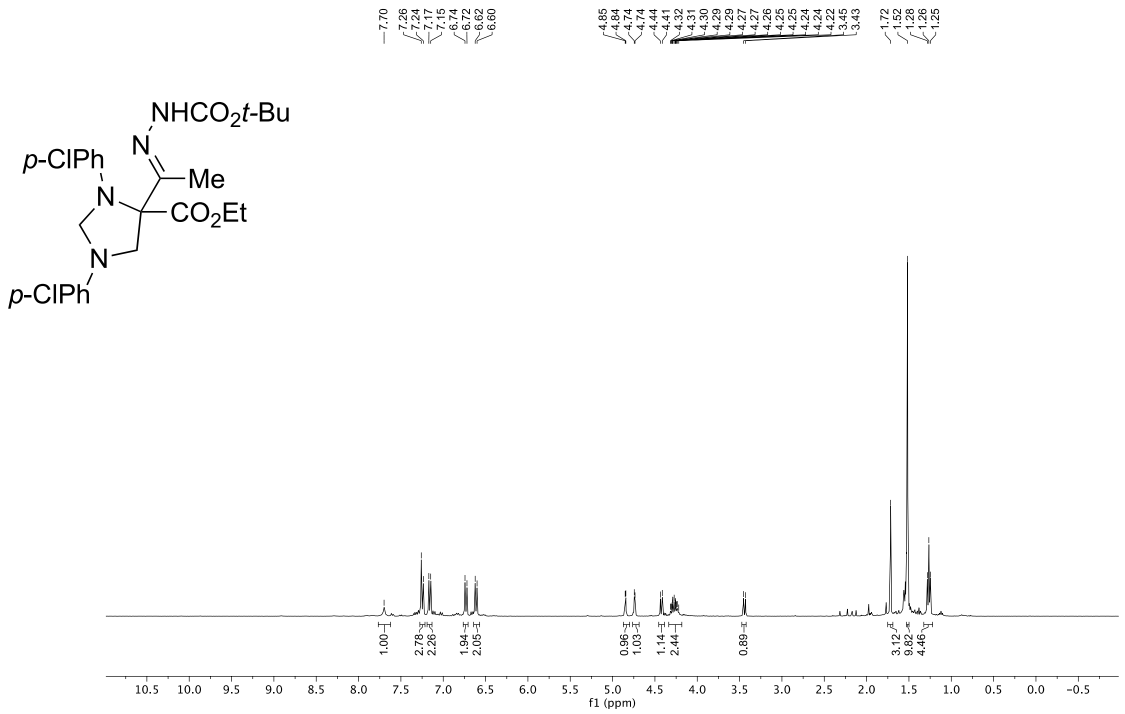

^{13}C {^{1}H} NMR (101 MHz, $CDCl_3$) of **3q**:

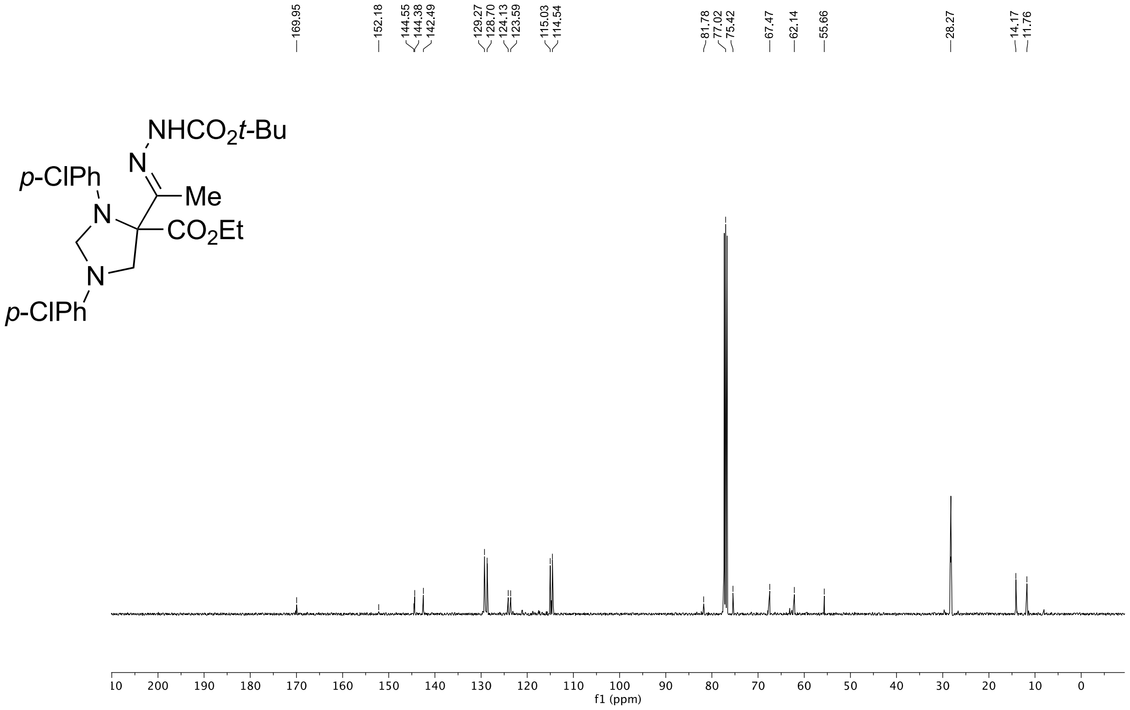

Ethyl-4-(1-(2-(tert-butoxycarbonyl)hydrazineylidene)ethyl)-1,3-bis(4-fluorophenyl)imidazolidine-4-carboxylate (3r)

^{1}H NMR (400 MHz, DMSO-d_6) of **3r**:

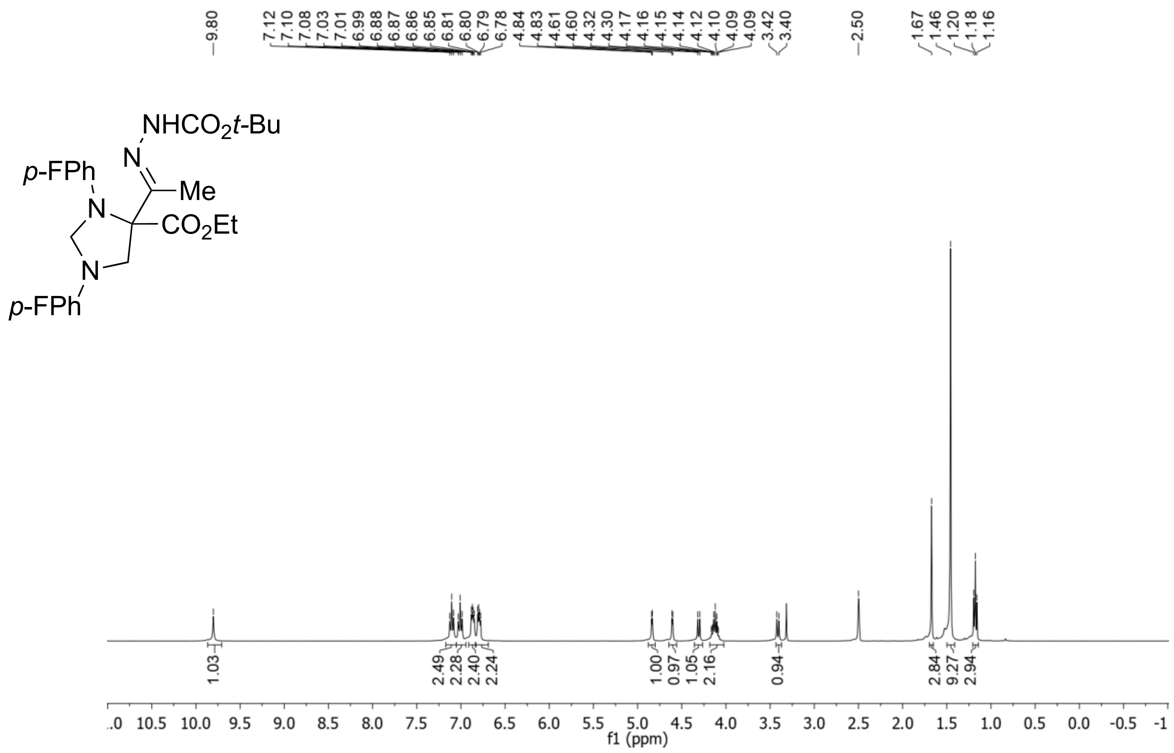

^{13}C {^{1}H} NMR (101 MHz, DMSO-d_6) of **3r**:

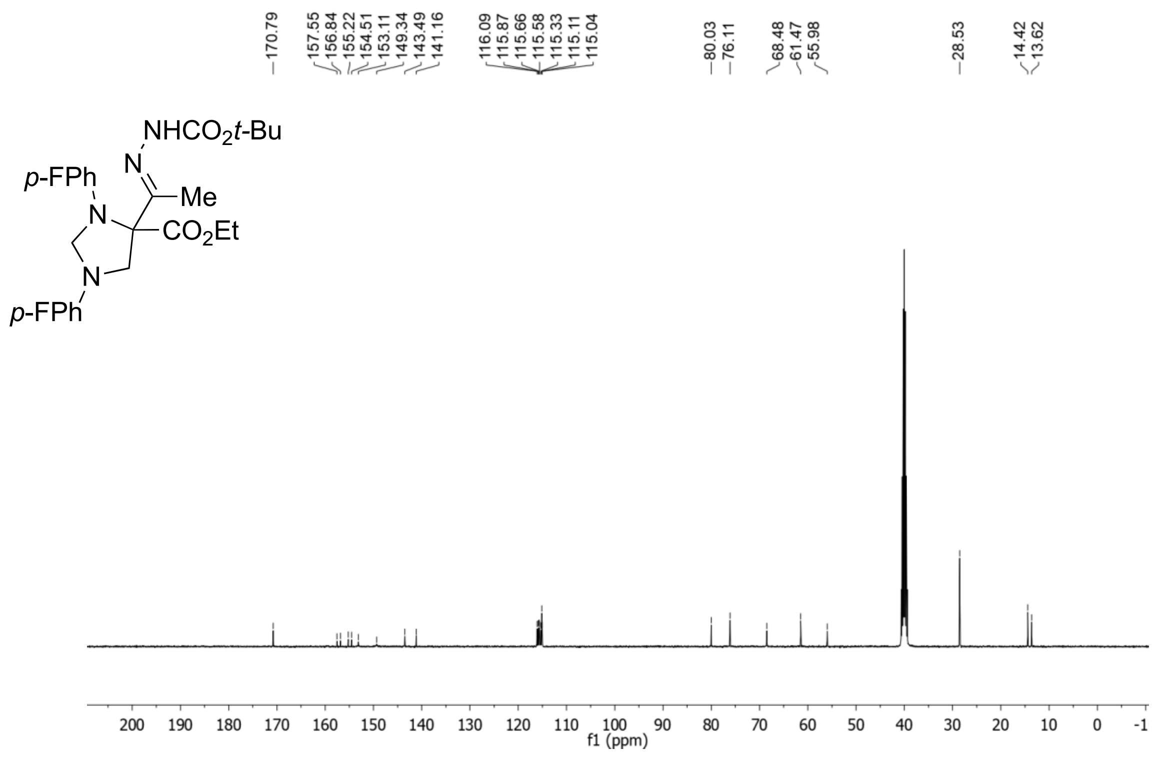

$^{19}F\{^1H\}$ NMR (376 MHz, DMSO-d_6) of **3r**:

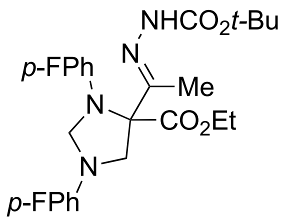

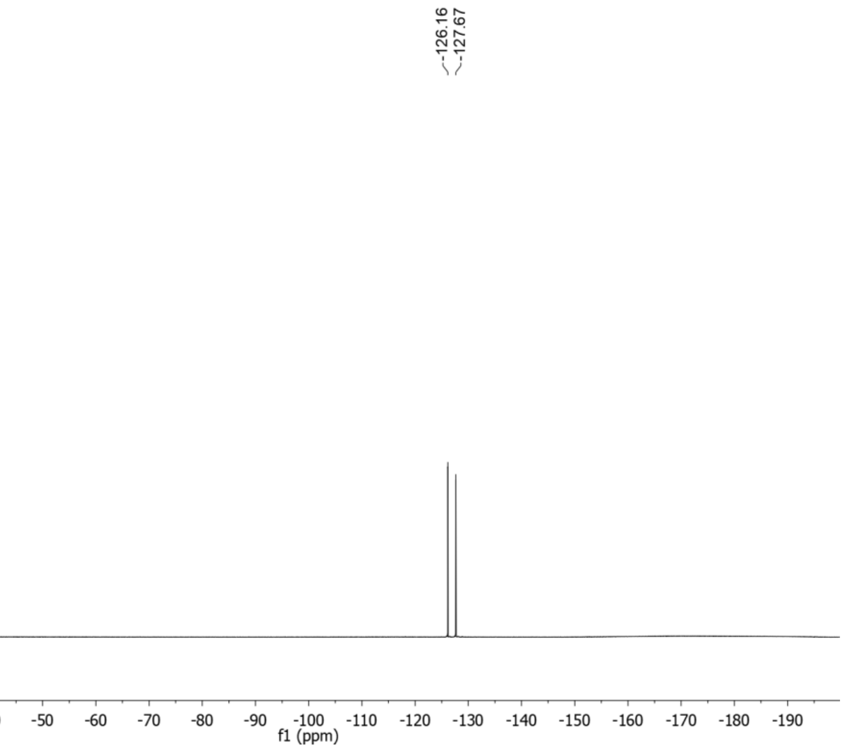

Ethyl-4-(1-(2-(*tert*-butoxycarbonyl)hydrazineylidene)ethyl)-1-(4-methoxyphenyl)-3-phenylimidazolidine-4-carboxylate (3Ab)

^{1}H NMR (400 MHz, DMSO-d_6) of **3Ab**:

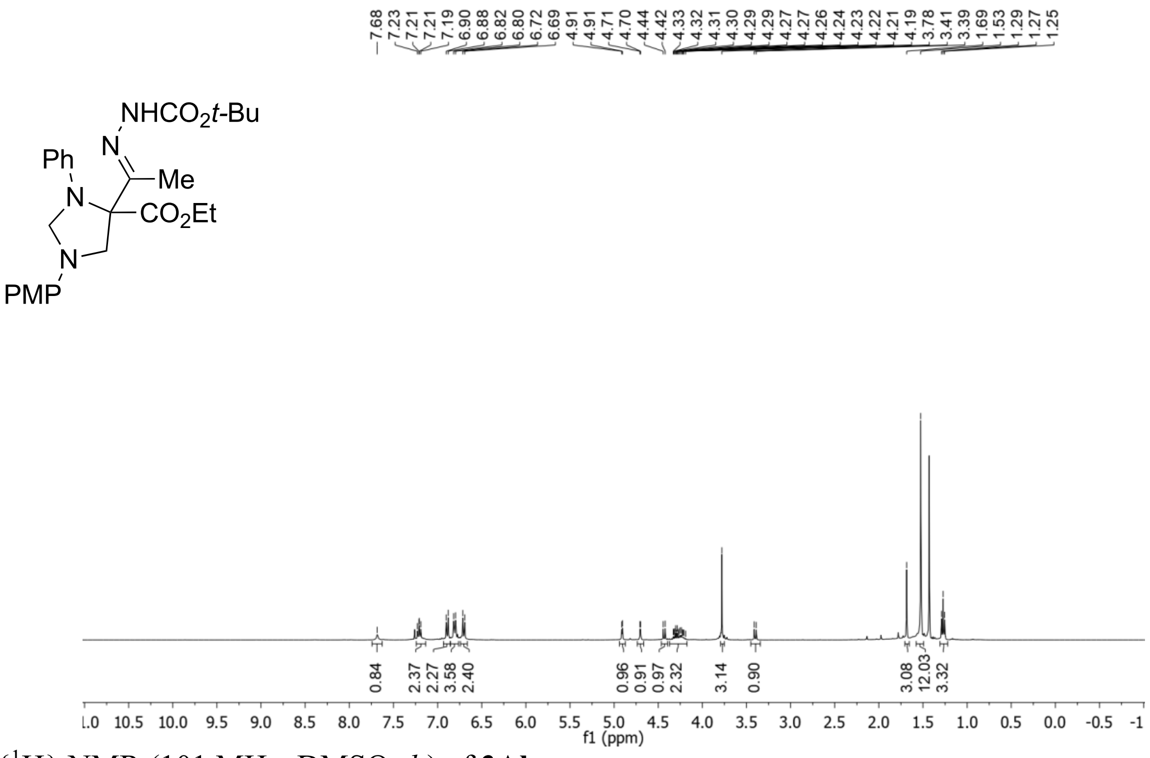

^{13}C {^{1}H} NMR (101 MHz, DMSO-d_6) of **3Ab:**

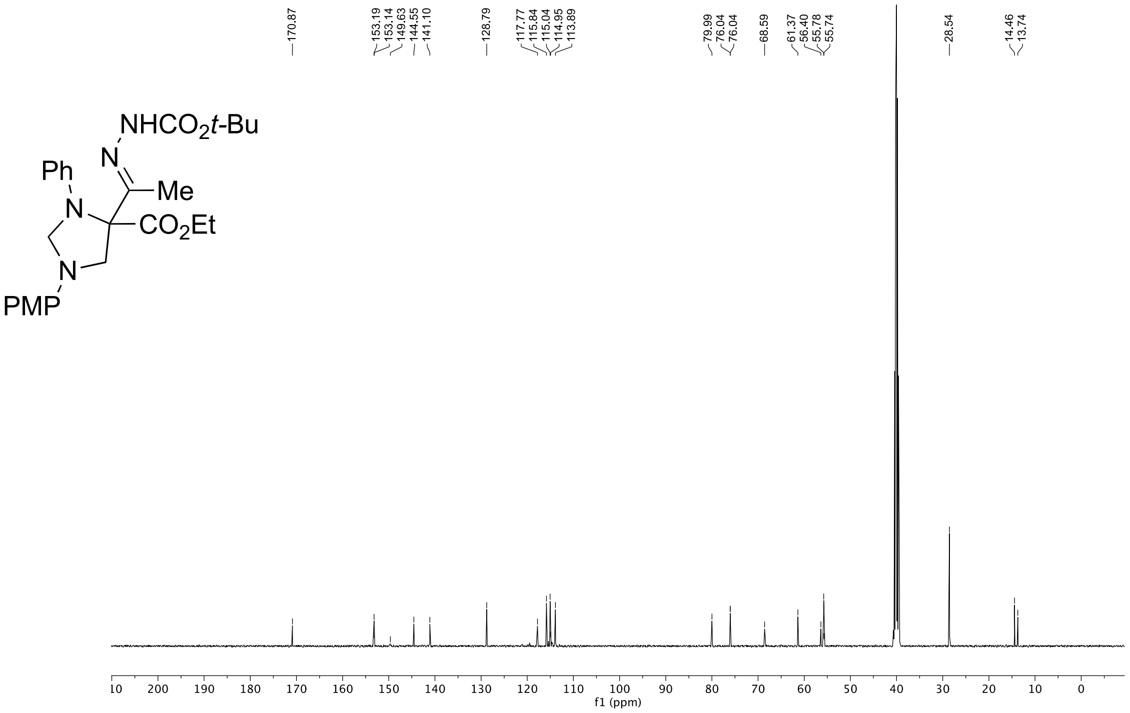

Ethyl-4-(1-(2-(*tert*-butoxycarbonyl)hydrazineylidene)ethyl)-3-phenyl-1-(*p*-tolyl)imidazolidine-4-carboxylate (3Ac)

^{1}H NMR (400 MHz, DMSO-d_6) of **3Ac**:

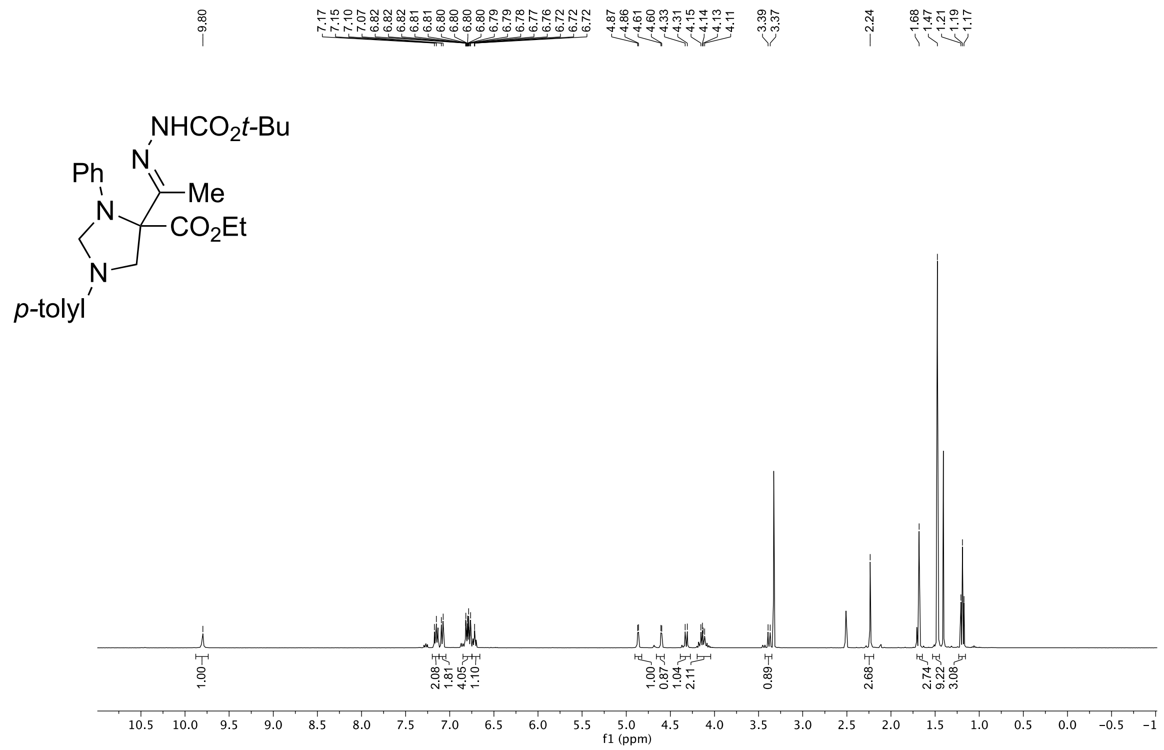

^{13}C {^{1}H} NMR (101 MHz, DMSO-d_6) of **3Ac:**

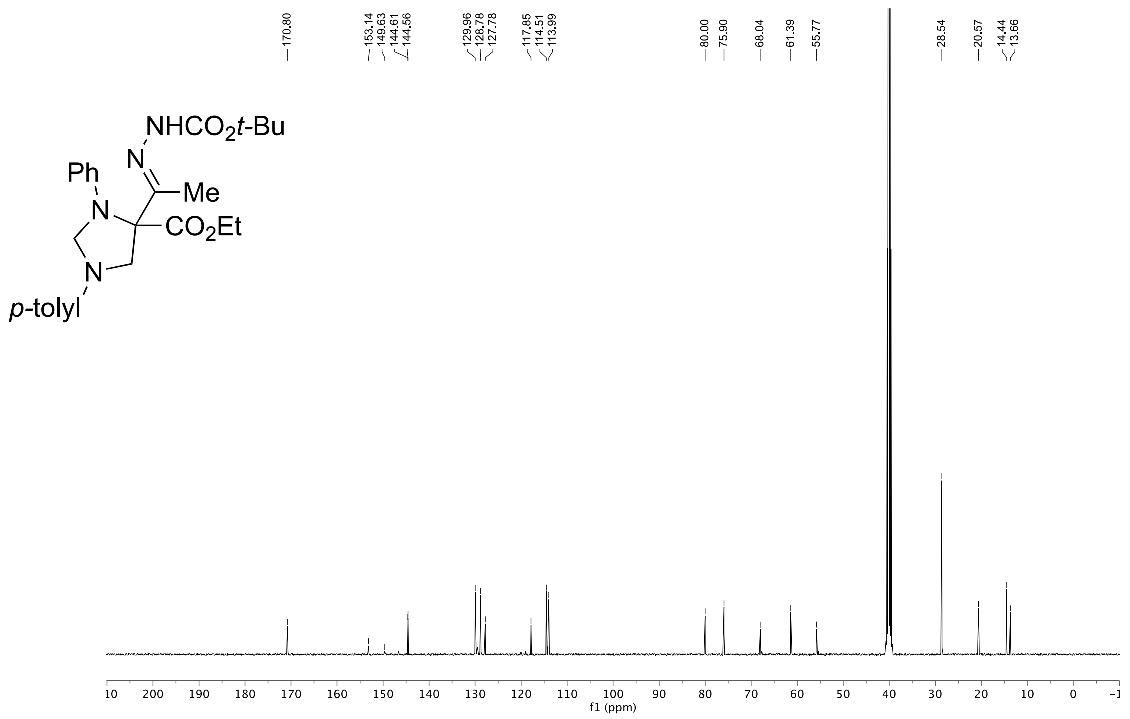

Ethyl (*E/Z*)-4-(1-(2-(*tert*-butoxycarbonyl)hydrazineylidene)ethyl)-1-(4-chlorophenyl)-3-phenylimidazolidine-4-carboxylate (3Ad)

^{1}H NMR (400 MHz, DMSO-d_6) of **3Ad**:

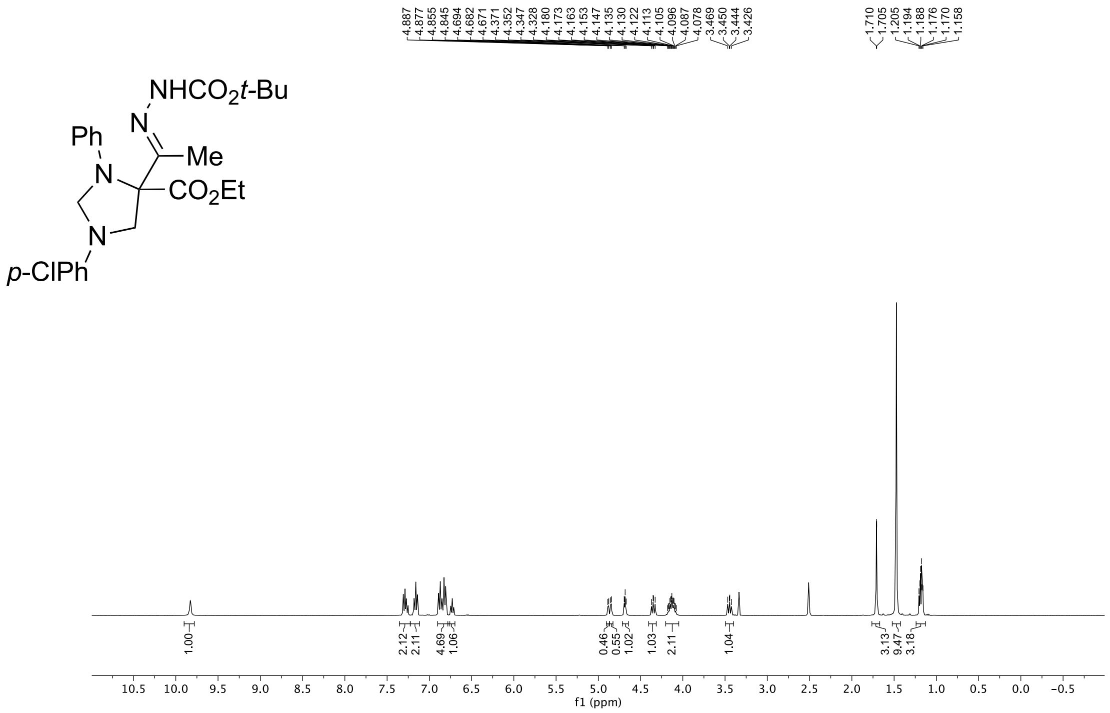

^{13}C {^{1}H} NMR (101 MHz, DMSO-d_6) of **3Ad:**

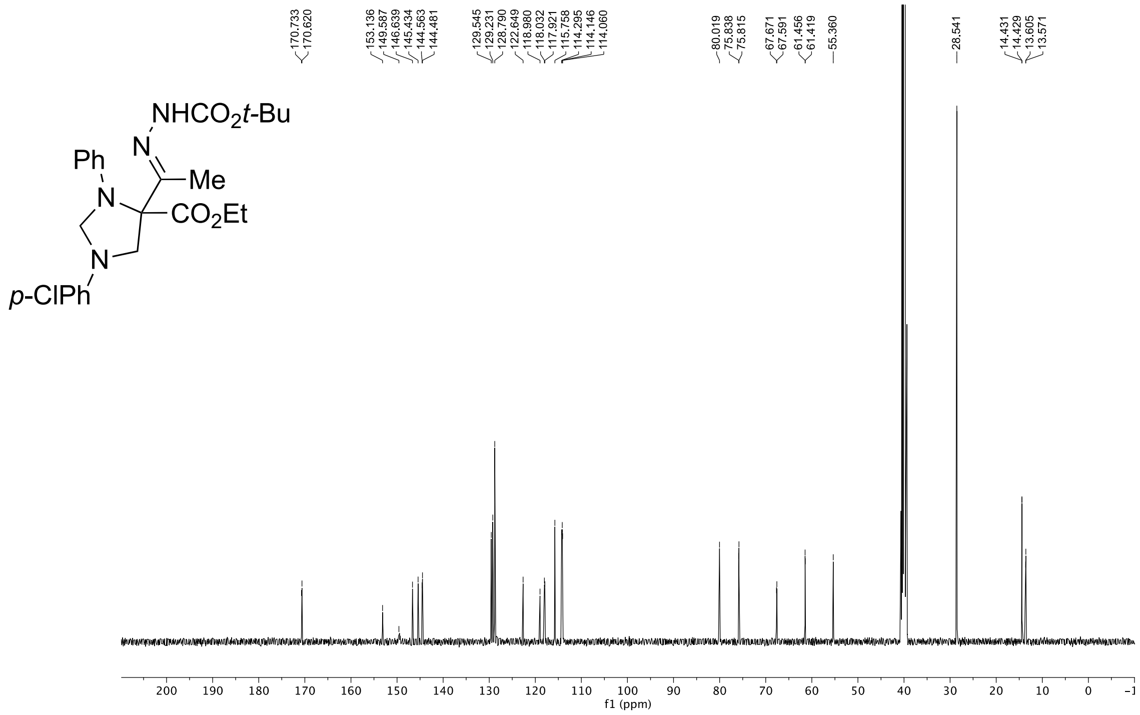

Ethyl-4-(1-(2-(*tert*-butoxycarbonyl)hydrazineylidene)ethyl)-1-(4-fluorophenyl)-3-phenylimidazolidine-4-carboxylate (3Ae)

^{1}H NMR (400 MHz, DMSO-d_6) of **3Ae**:

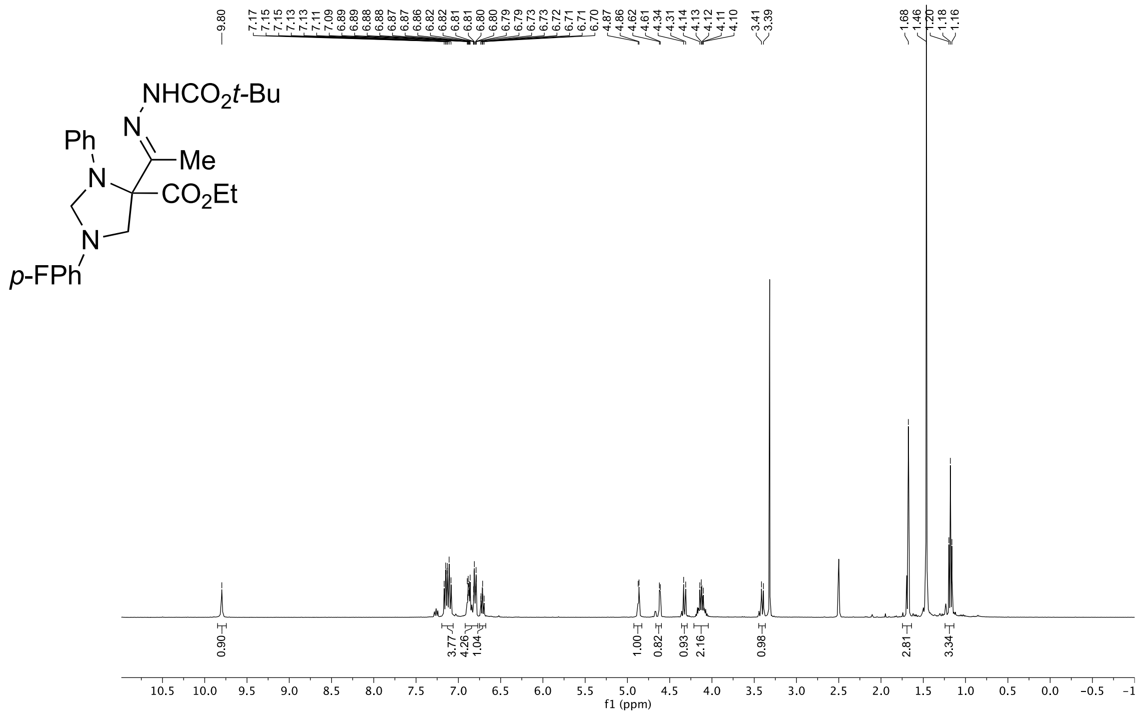

^{13}C {^{1}H} NMR (101 MHz, DMSO-d_6) of **3Ae:**

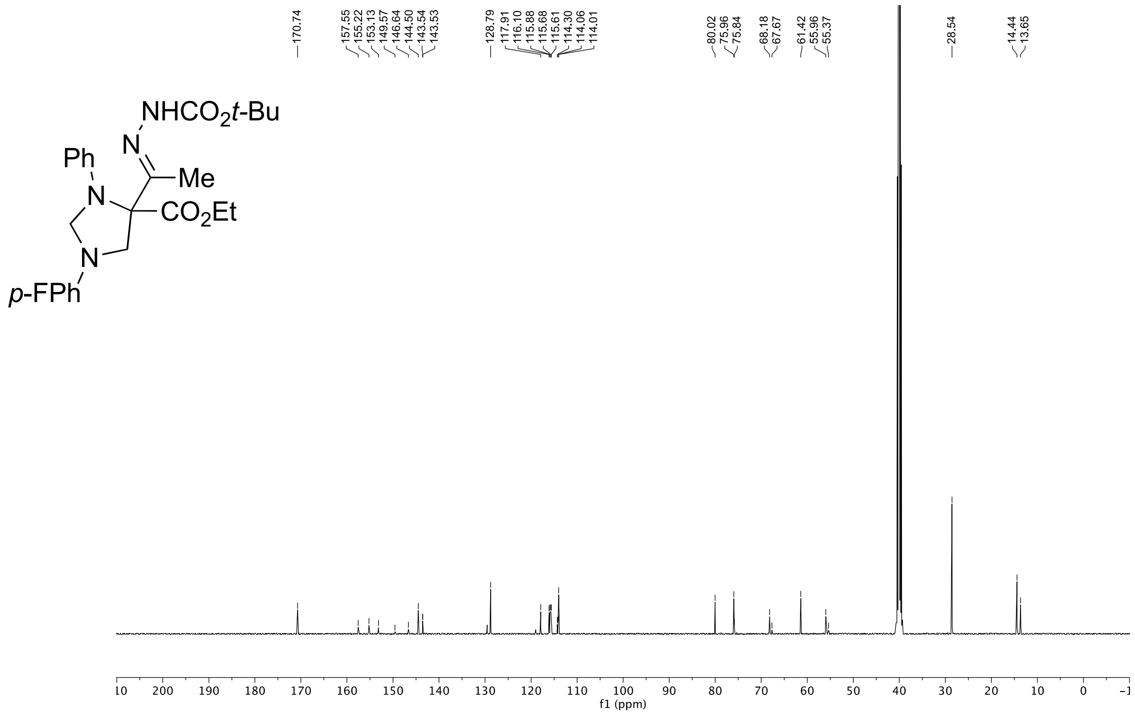

$^{19}F\{^{1}H\}$ NMR (376 MHz, DMSO-d_6) of **3Ae**:

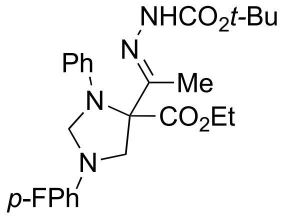

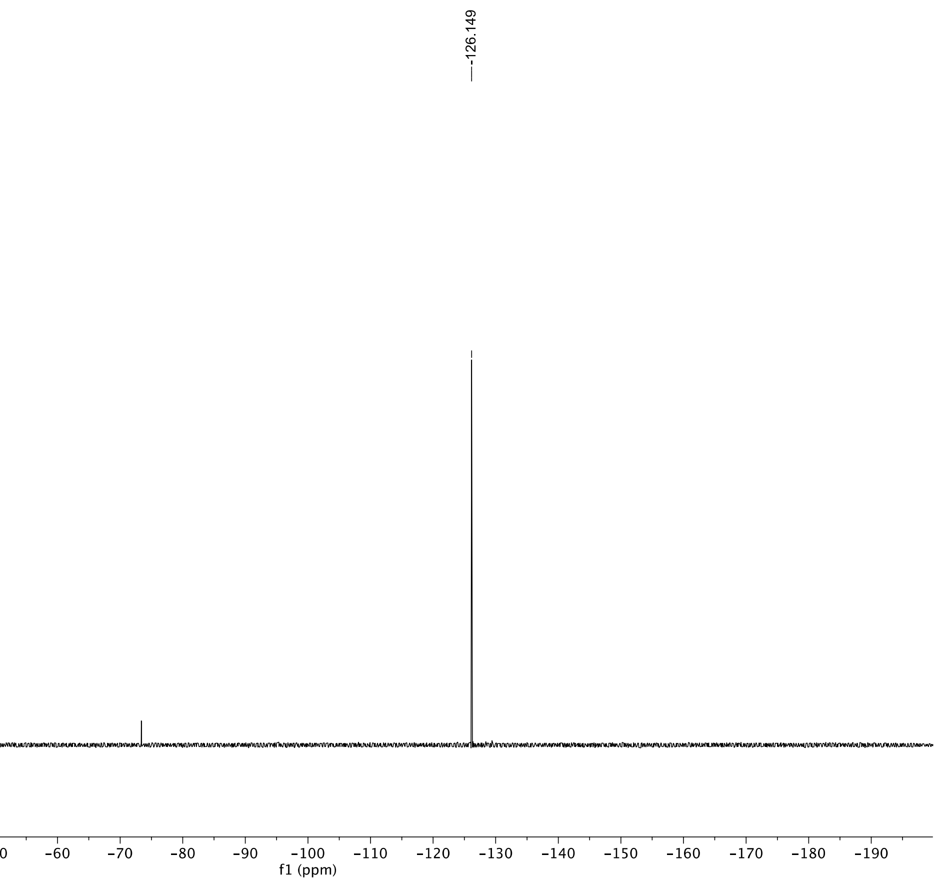

Ethyl-4-(1-(2-(*tert*-butoxycarbonyl)hydrazineylidene)ethyl)-3-(4-methoxyphenyl)-1-phenylimidazolidine-4-carboxylate (3Ba)

^{1}H NMR (400 MHz, DMSO-d_6) of **3Ba**:

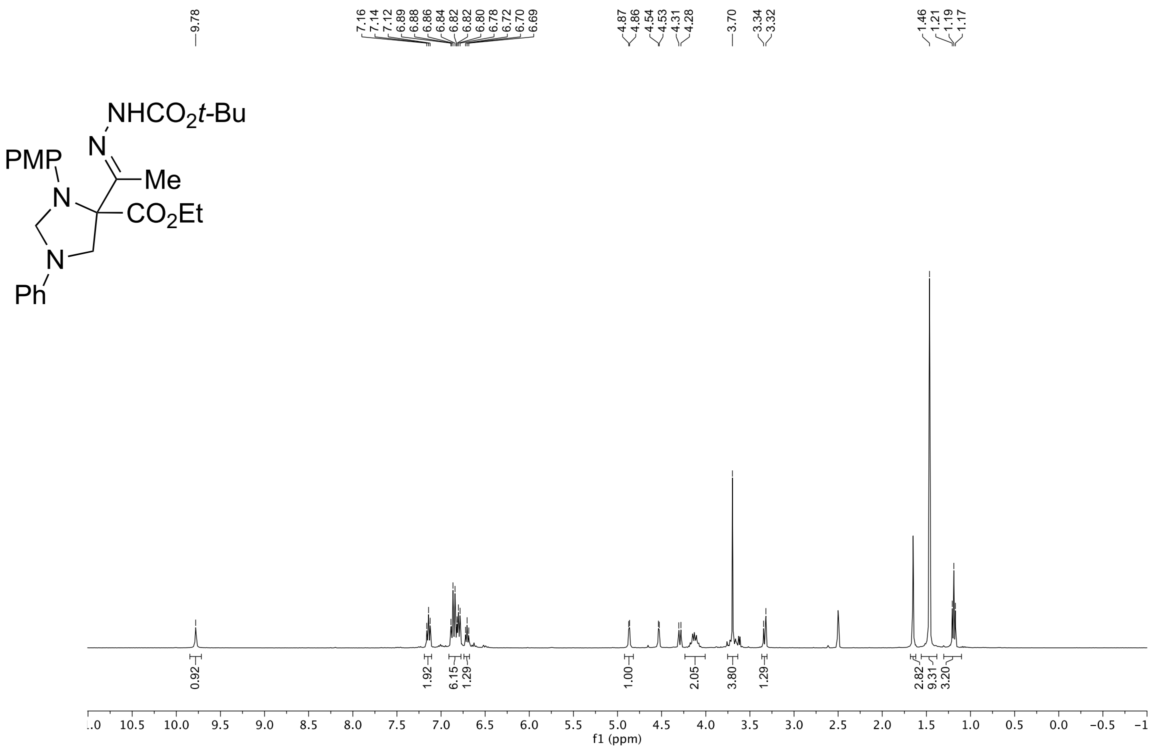

^{13}C {^{1}H} NMR (101 MHz, DMSO-d_6) of **3Ba:**

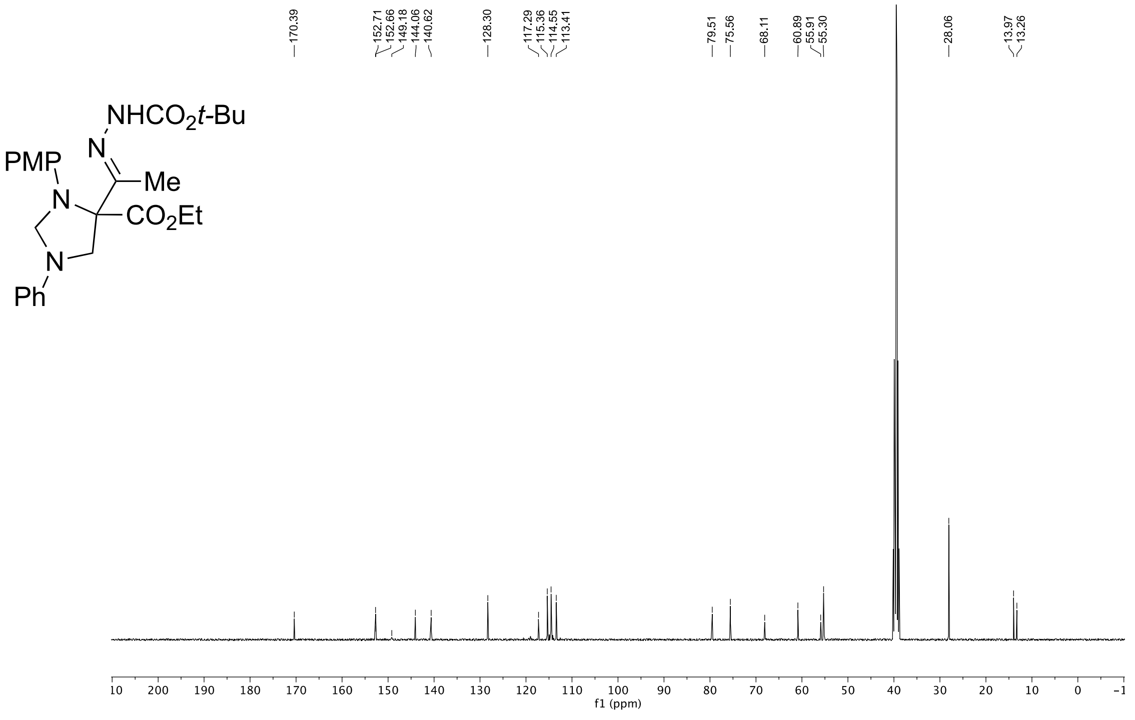

Ethyl-4-(1-(2-(*tert*-butoxycarbonyl)hydrazineylidene)ethyl)-3-(4-methoxyphenyl)-1-(p-tolyl)imidazolidine-4-carboxylate (3Bc):

^{1}H NMR (400 MHz, DMSO-d_6) of **3Bc**:

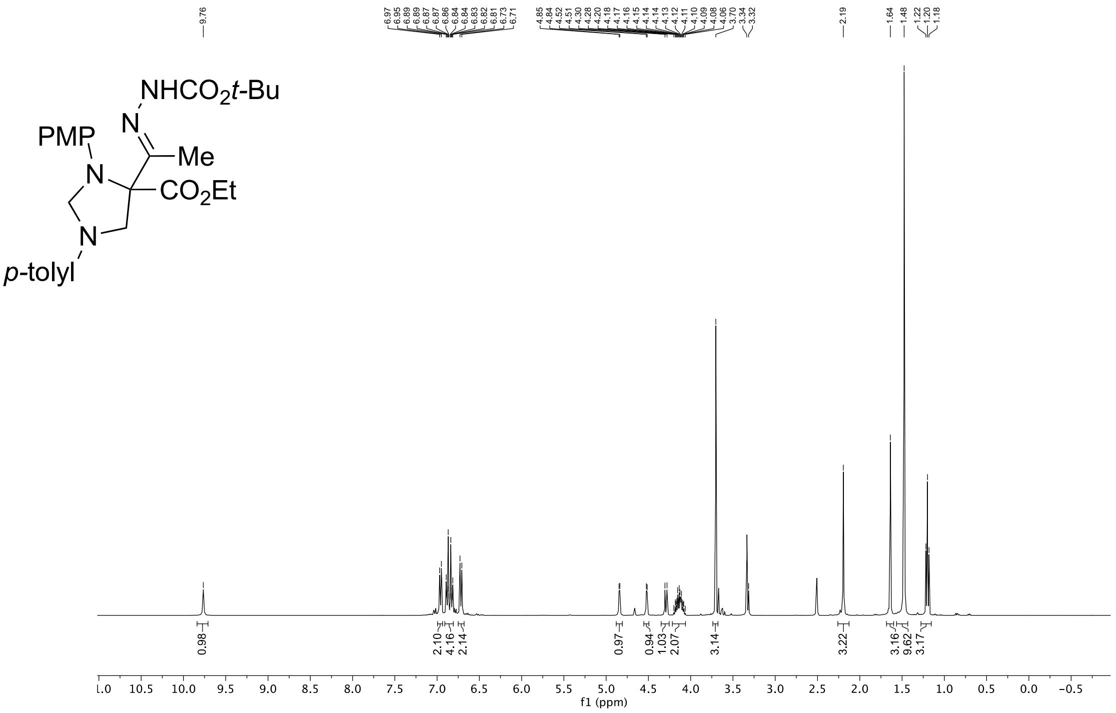

^{13}C {^{1}H} NMR (101 MHz, DMSO-d_6) of **3Bc:**

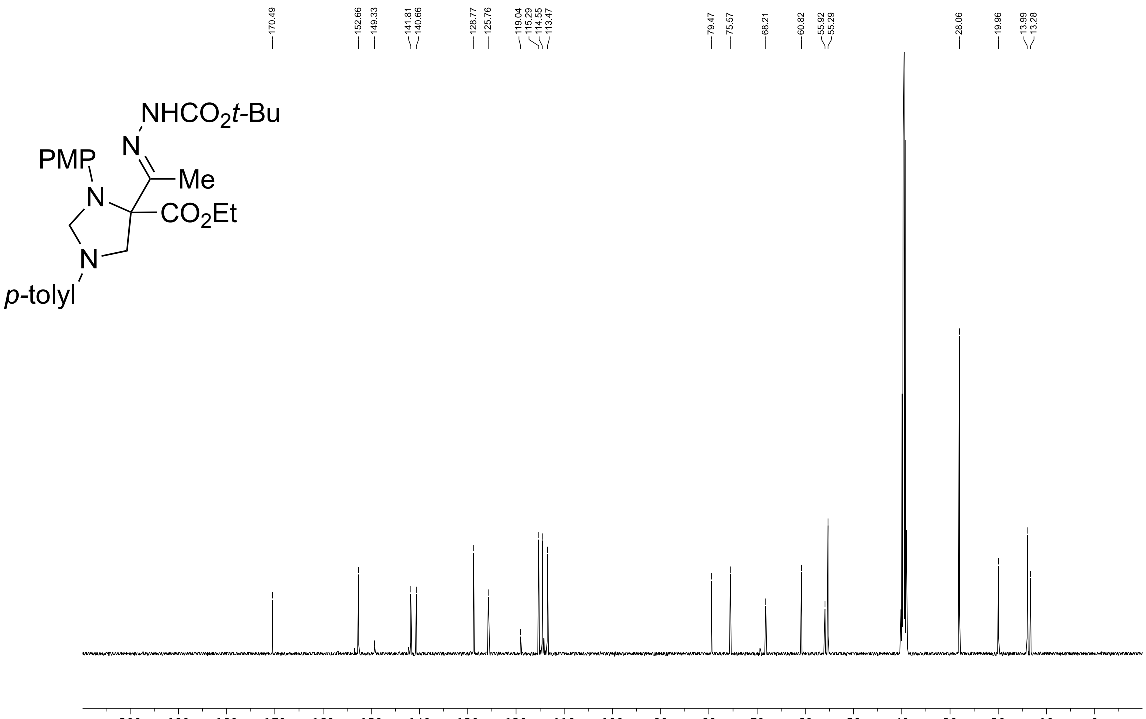

Ethyl-4-(1-(2-(*tert*-butoxycarbonyl)hydrazineylidene)ethyl)-1-(4-chlorophenyl)-3-(4-methoxyphenyl)imidazolidine-4-carboxylate (3Bd):

^{1}H NMR (400 MHz, DMSO-d_6) of **3Bd**:

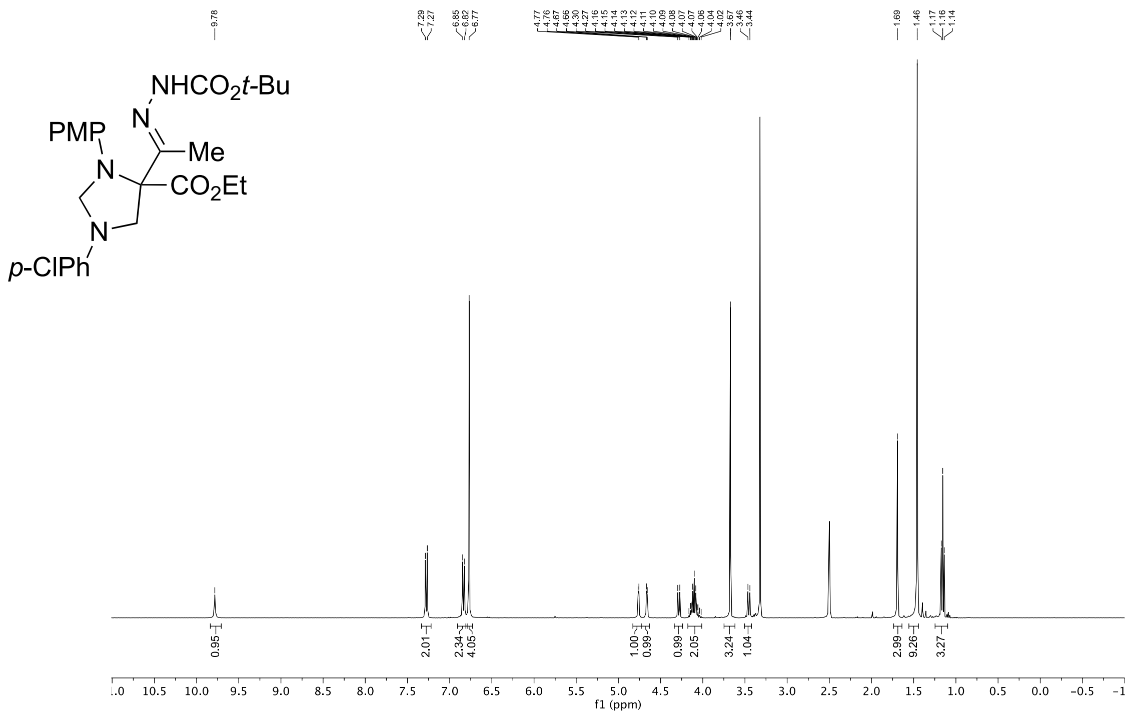

^{13}C {^{1}H} NMR (101 MHz, DMSO-d_6) of **3Bd:**

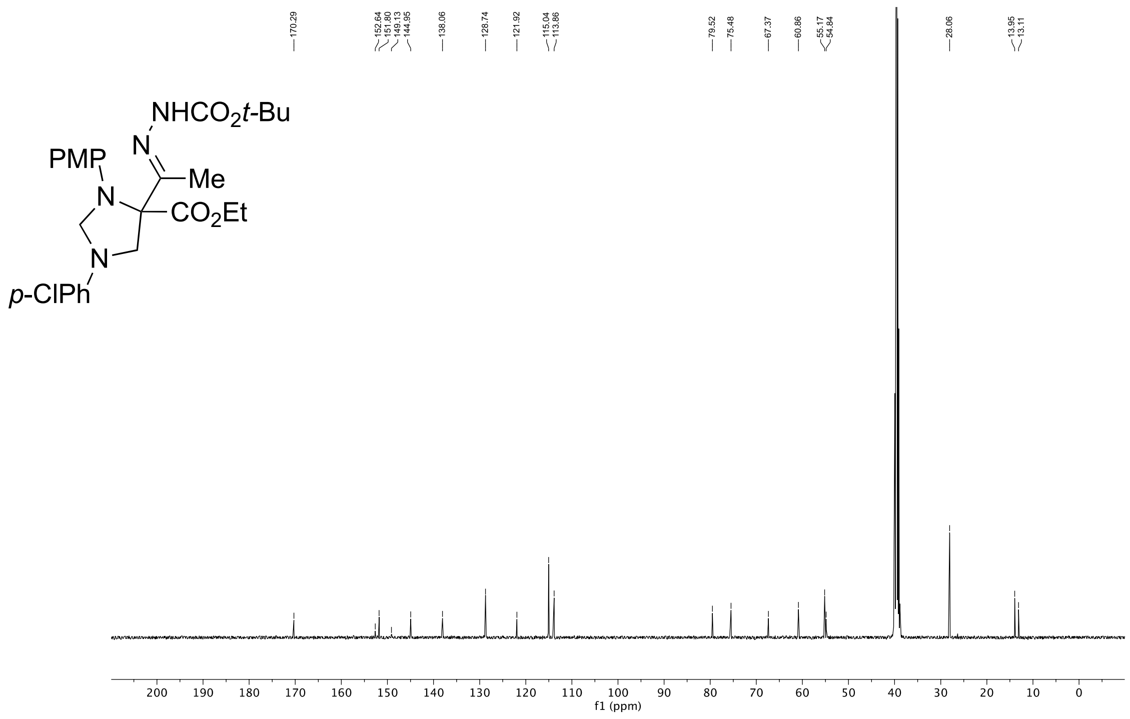

Ethyl-4-(1-(2-(*tert*-butoxycarbonyl)hydrazineylidene)ethyl)-1-(4-fluorophenyl)-3-(4-methoxyphenyl)imidazolidine-4-carboxylate (3Be):

^{1}H NMR (400 MHz, DMSO-d_6) of **3Be**:

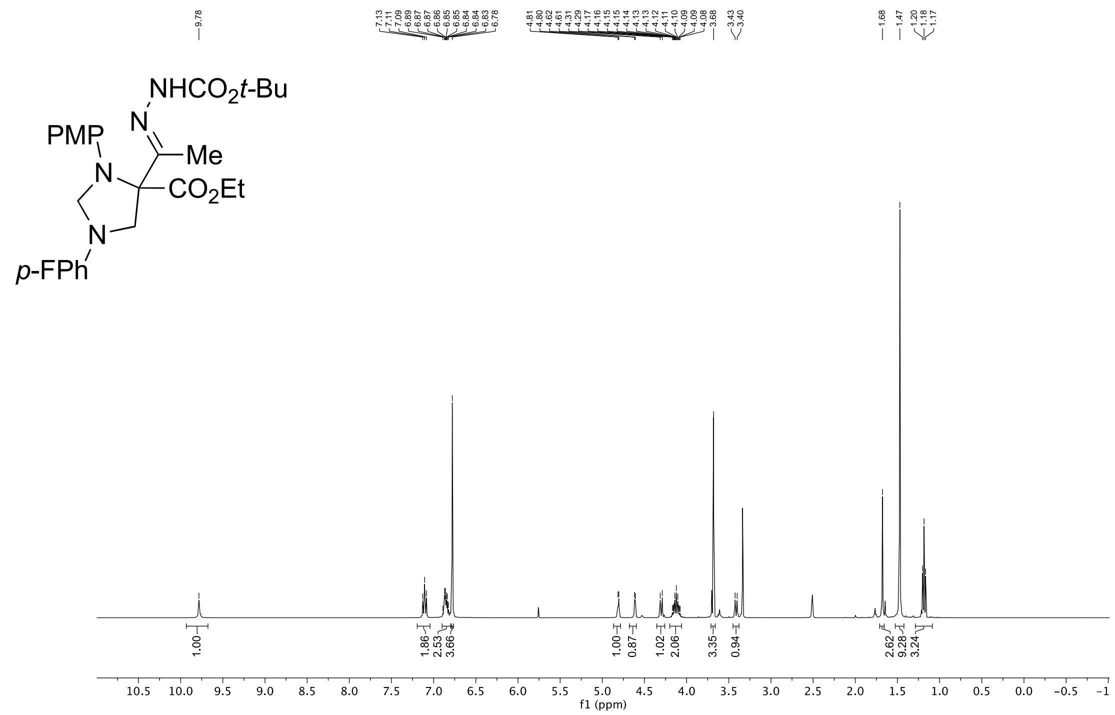

^{13}C {^{1}H} NMR (101 MHz, DMSO-d_6) of **3Be:**

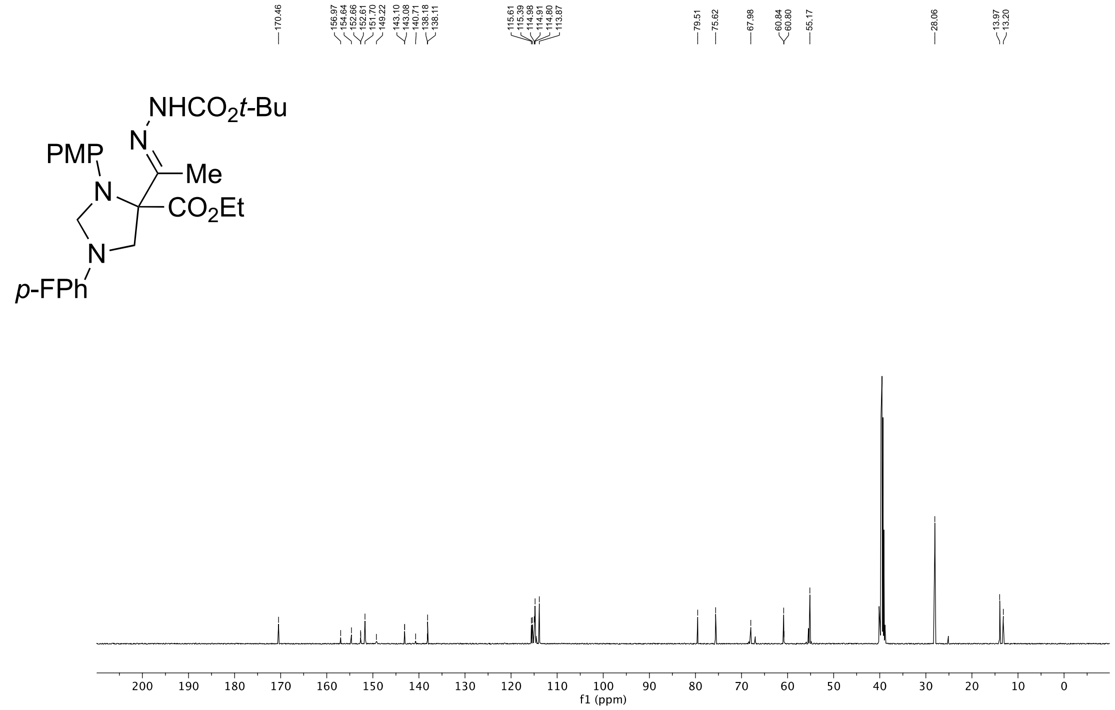

$^{19}F\{^1H\}$ NMR (376 MHz, DMSO-d_6) of **3Be**:

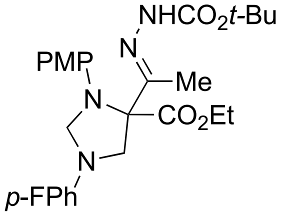

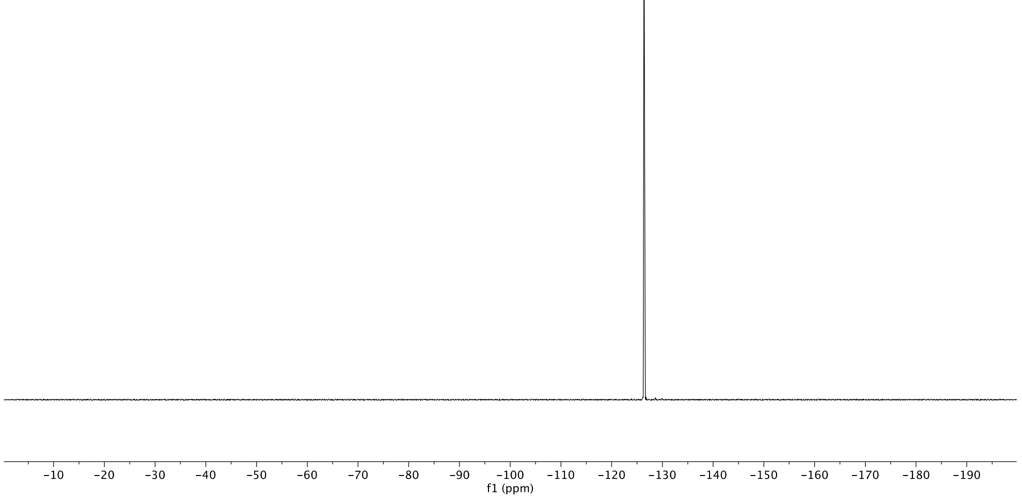

Ethyl (*E/Z*)-4-(1-(2-(*tert*-butoxycarbonyl)hydrazineylidene)ethyl)-1-phenyl-3-(*p*-tolyl)imidazolidine-4-carboxylate (3Ca)

^{1}H NMR (400 MHz, DMSO-d_6) of **3Ca**:

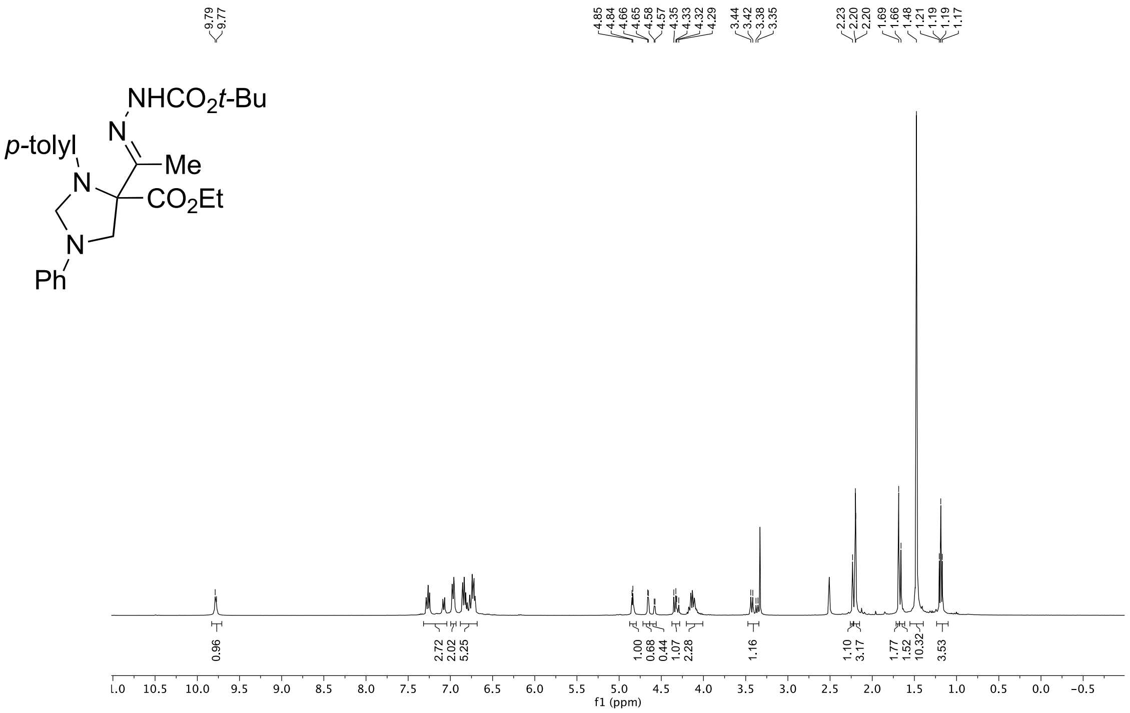

^{13}C {^{1}H} NMR (101 MHz, DMSO-d_6) of **3Ca:**

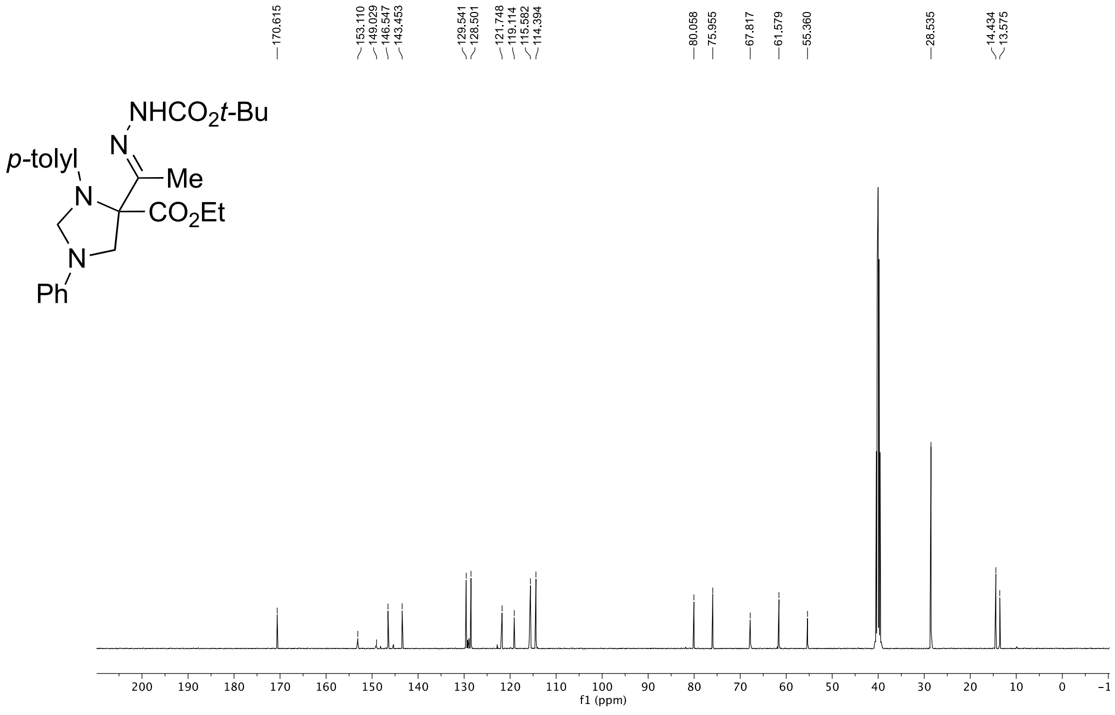

Ethyl-4-(1-(2-(*tert*-butoxycarbonyl)hydrazineylidene)ethyl)-1-(4-methoxyphenyl)-3-(p-tolyl)imidazolidine-4-carboxylate (3Cb):

^{1}H NMR (400 MHz, DMSO-d_6) of **3Cb**:

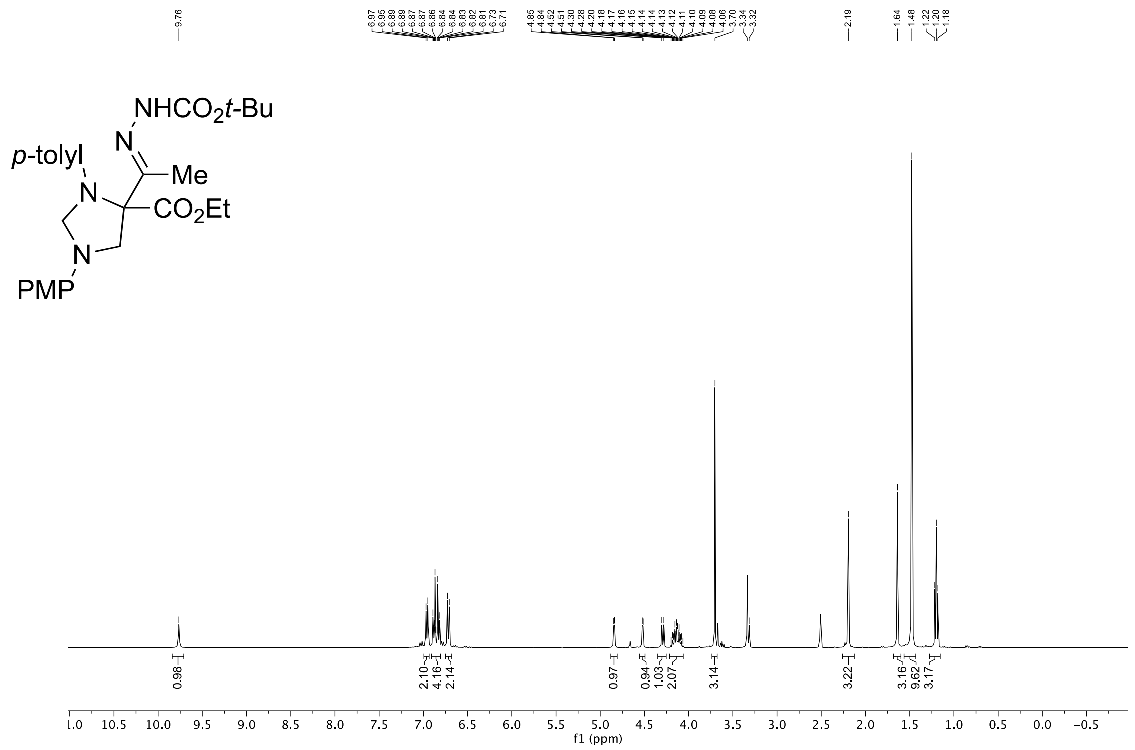

^{13}C {^{1}H} NMR (101 MHz, DMSO-d_6) of **3Cb:**

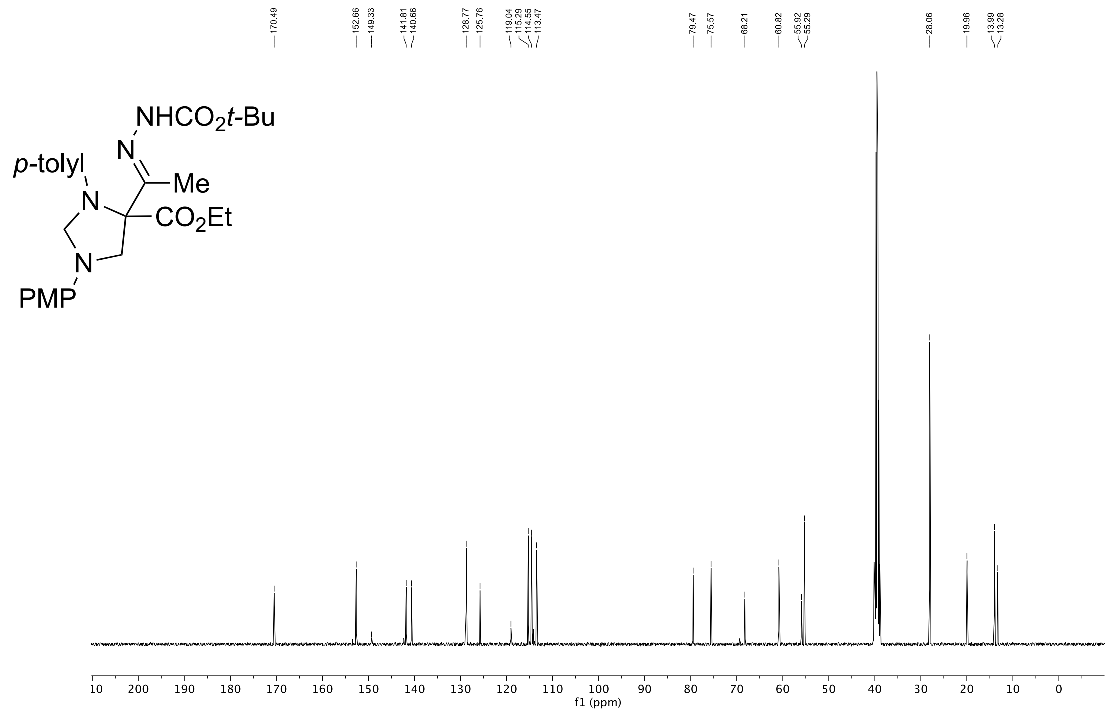

Ethyl-4-(1-(2-(*tert*-butoxycarbonyl)hydrazineylidene)ethyl)-1-(4-chlorophenyl)-3-(*p*-tolyl)imidazolidine-4-carboxylate (3Cd):

^{1}H NMR (400 MHz, DMSO-d_6) of **3Cd**:

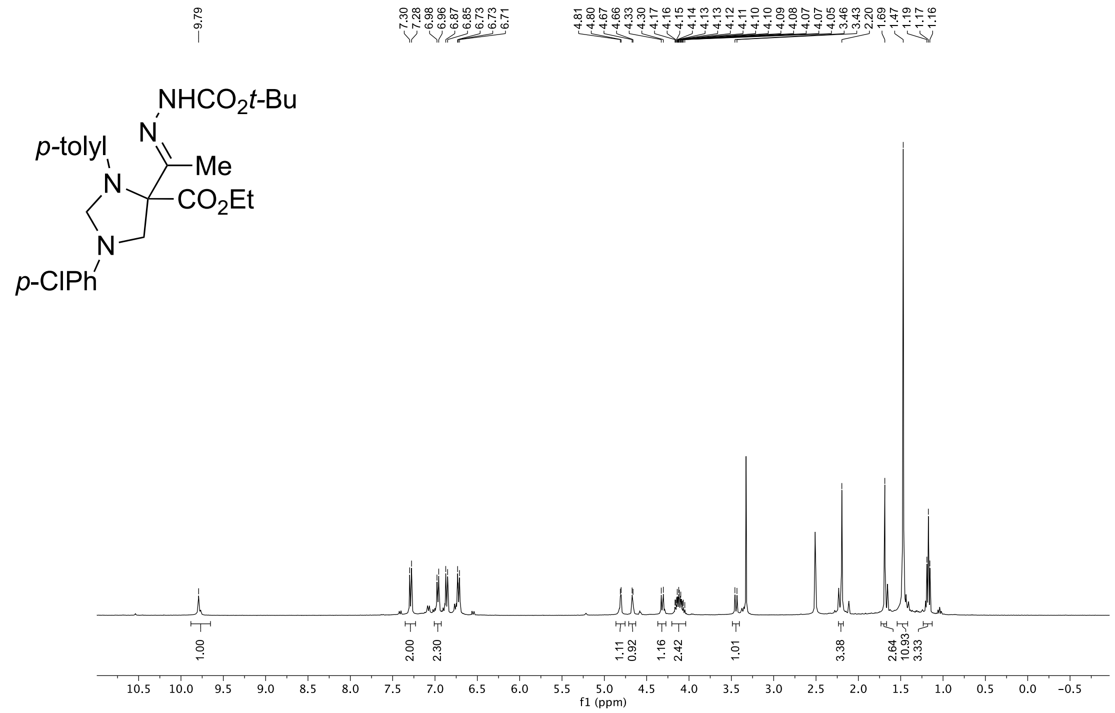

^{13}C {^{1}H} NMR (101 MHz, DMSO-d_6) of **3Cd:**

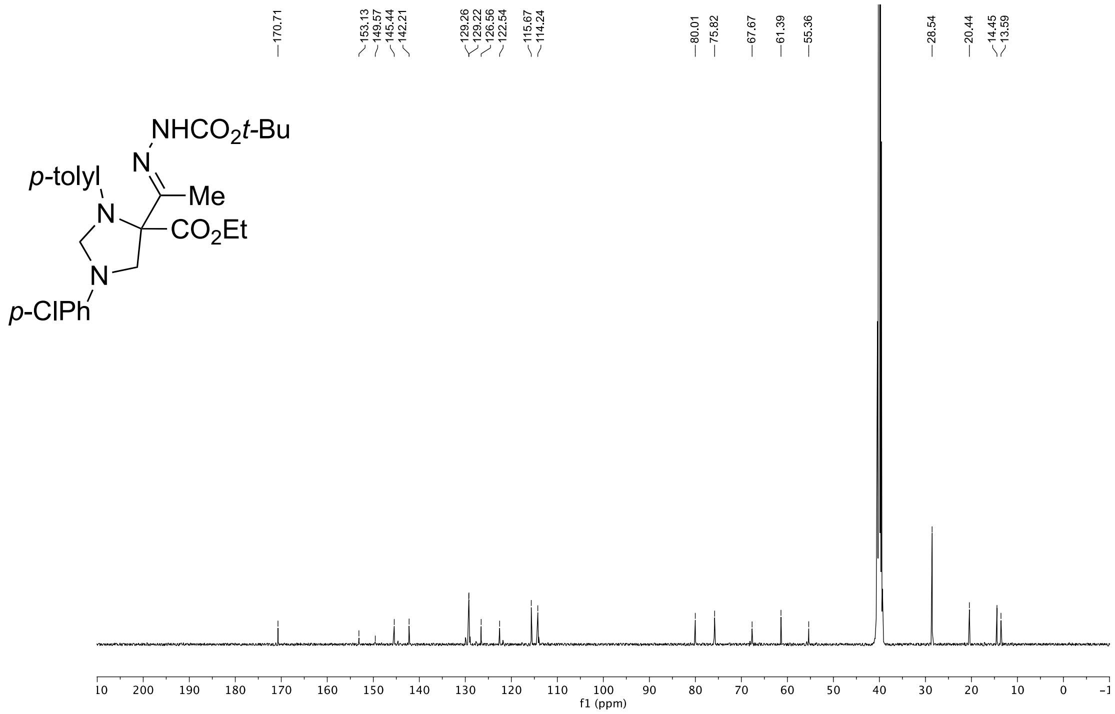

Ethyl-4-(1-(2-(*tert*-butoxycarbonyl)hydrazineylidene)ethyl)-1-(4-fluorophenyl)-3-(p-tolyl)imidazolidine-4-carboxylate (3Ce):

^{1}H NMR (400 MHz, DMSO-d_6) of **3Ce**:

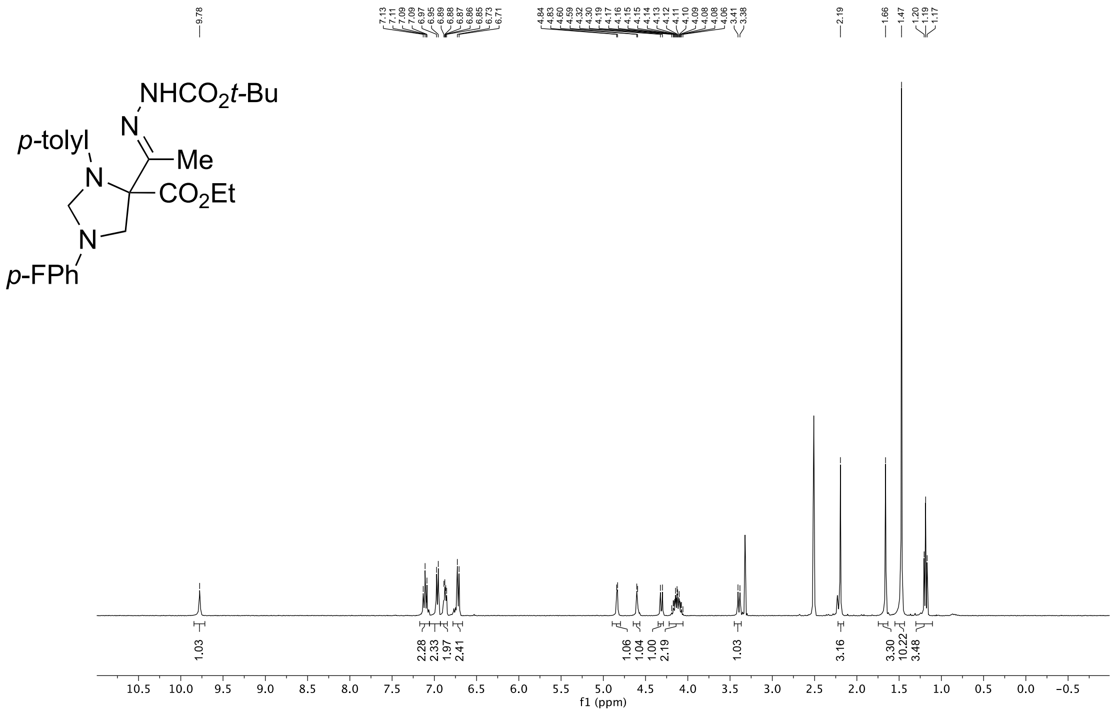

^{13}C {^{1}H} NMR (101 MHz, DMSO-d_6) of **3Ce:**

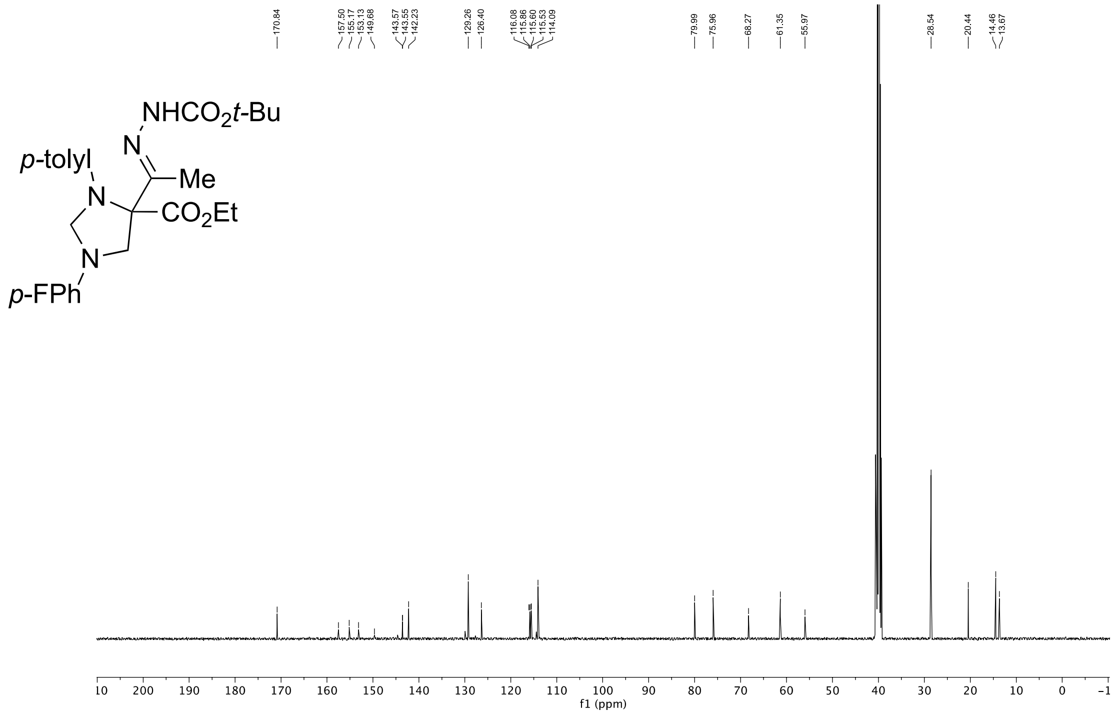

Ethyl-4-(1-(2-(*tert*-butoxycarbonyl)hydrazineylidene)ethyl)-3-(4-chlorophenyl)-1-phenylimidazolidine-4-carboxylate (3Da)

^{1}H NMR (400 MHz, DMSO-d_6) of **3Da:**

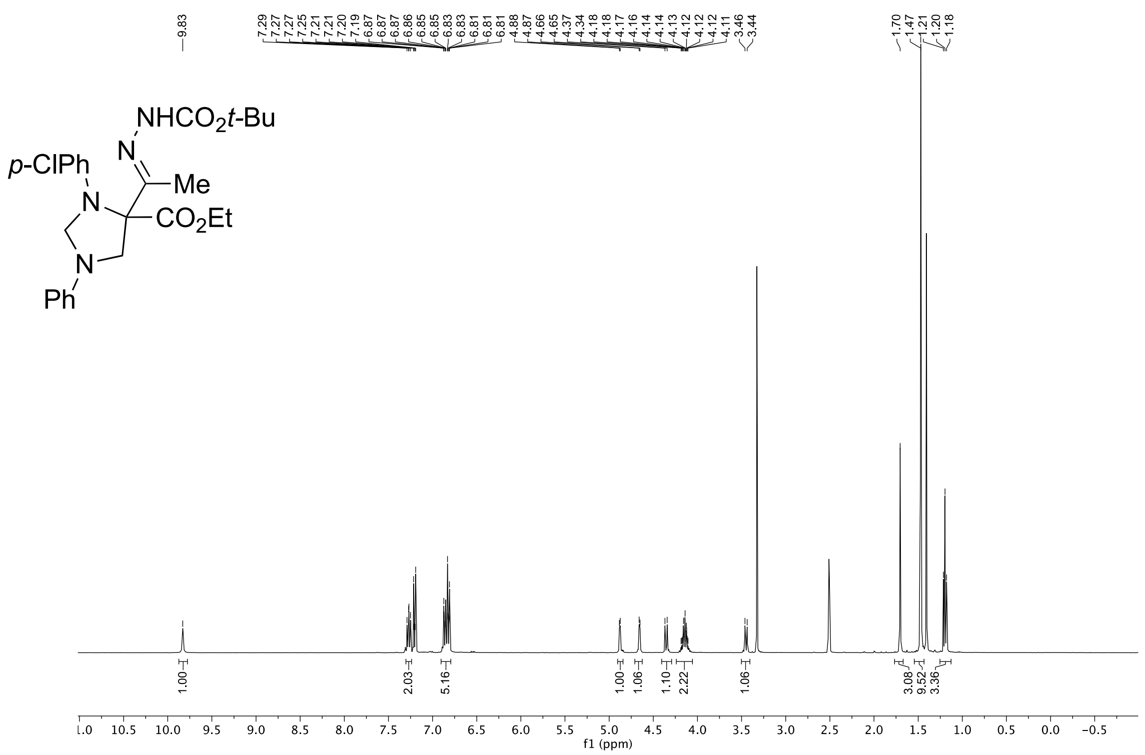

^{13}C {^{1}H} NMR (101 MHz, DMSO-d_6) of **3Da:**

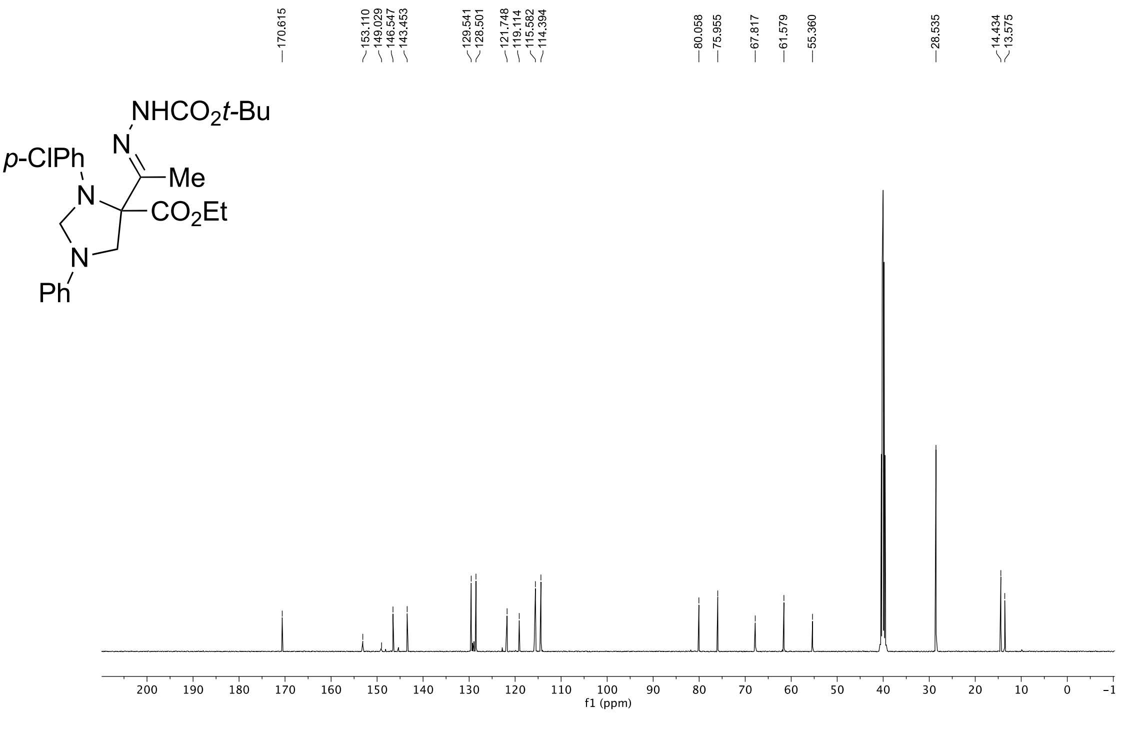

Ethyl-4-(1-(2-(*tert*-butoxycarbonyl)hydrazineylidene)ethyl)-3-(4-chlorophenyl)-1-(4-methoxyphenyl)imidazolidine-4-carboxylate (3Db):

^{1}H NMR (400 MHz, DMSO-d_6) of **3Db:**

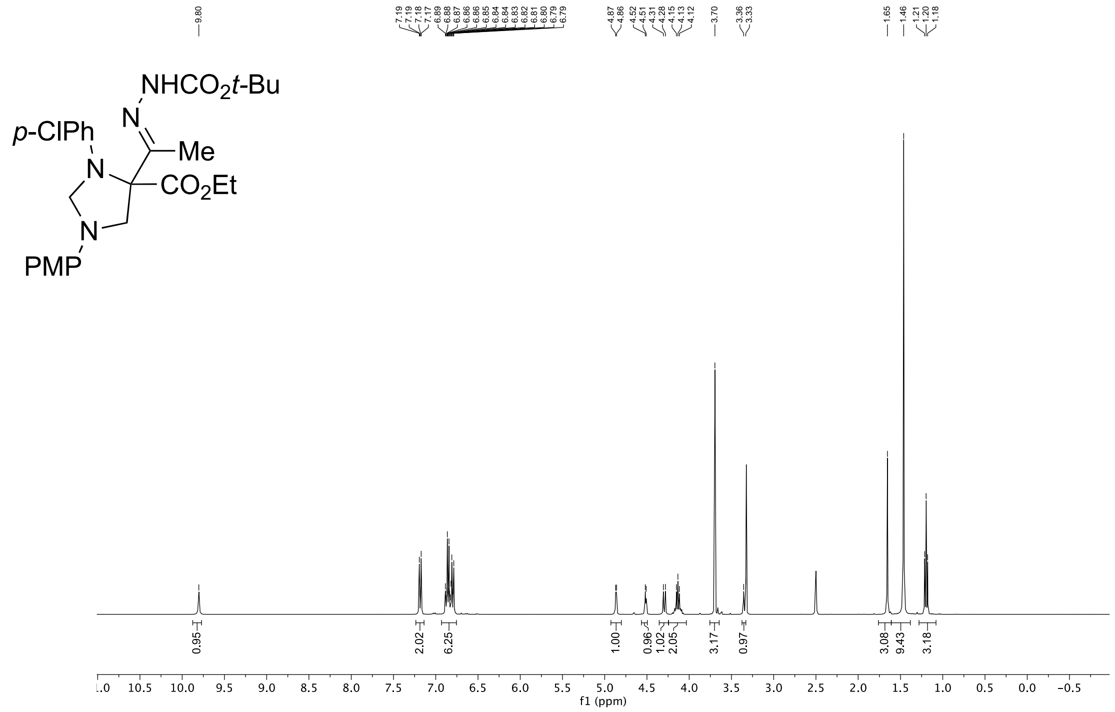

^{13}C {^{1}H} NMR (101 MHz, DMSO-d_6) of **3Db:**

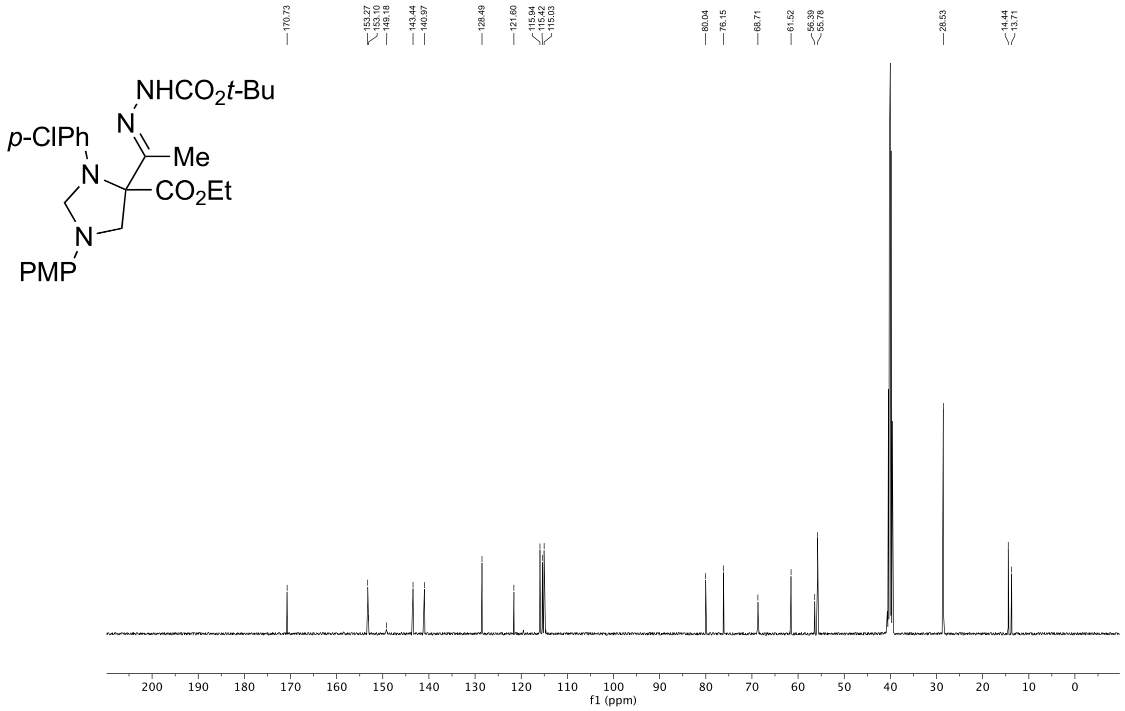

Ethyl (*E/Z*)-4-(1-(2-(*tert*-butoxycarbonyl)hydrazineylidene)ethyl)-3-(4-fluorophenyl)-1-phenylimidazolidine-4-carboxylate (3Ea)

^{1}H NMR (400 MHz, DMSO-d_6) of **3Ea:**

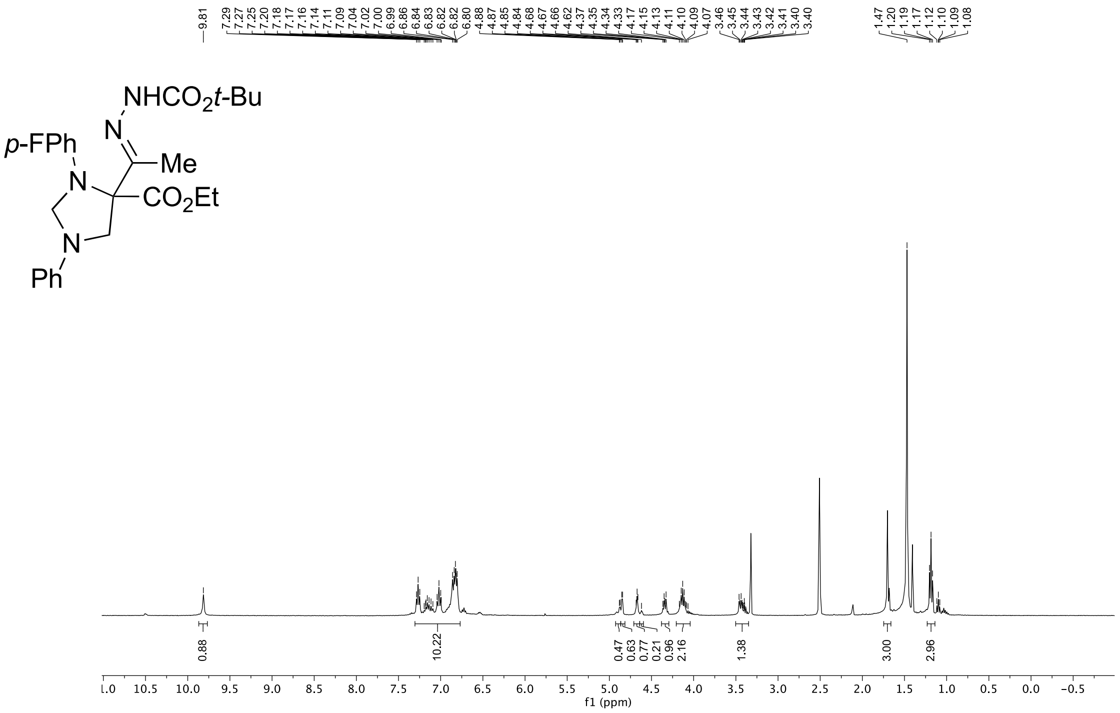

^{13}C {^{1}H} NMR (101 MHz, DMSO-d_6) of **3Ea:**

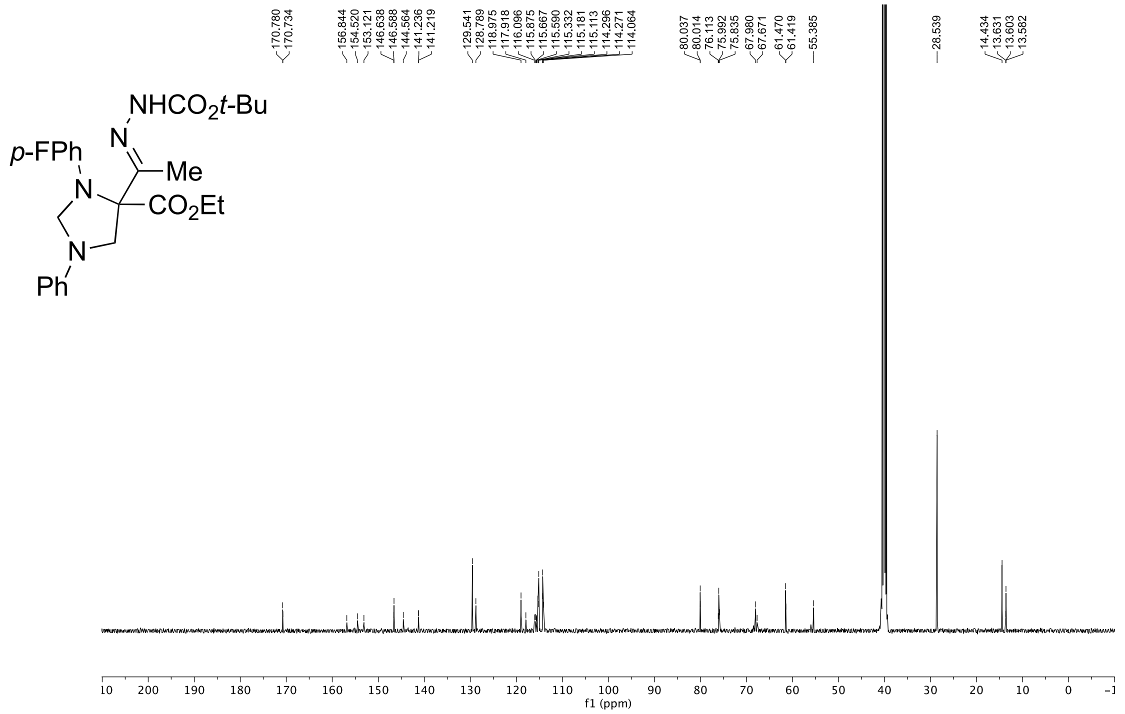

$^{19}F\{^{1}H\}$ NMR (376 MHz, DMSO-d_6) of **3Ea**:

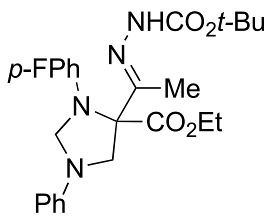

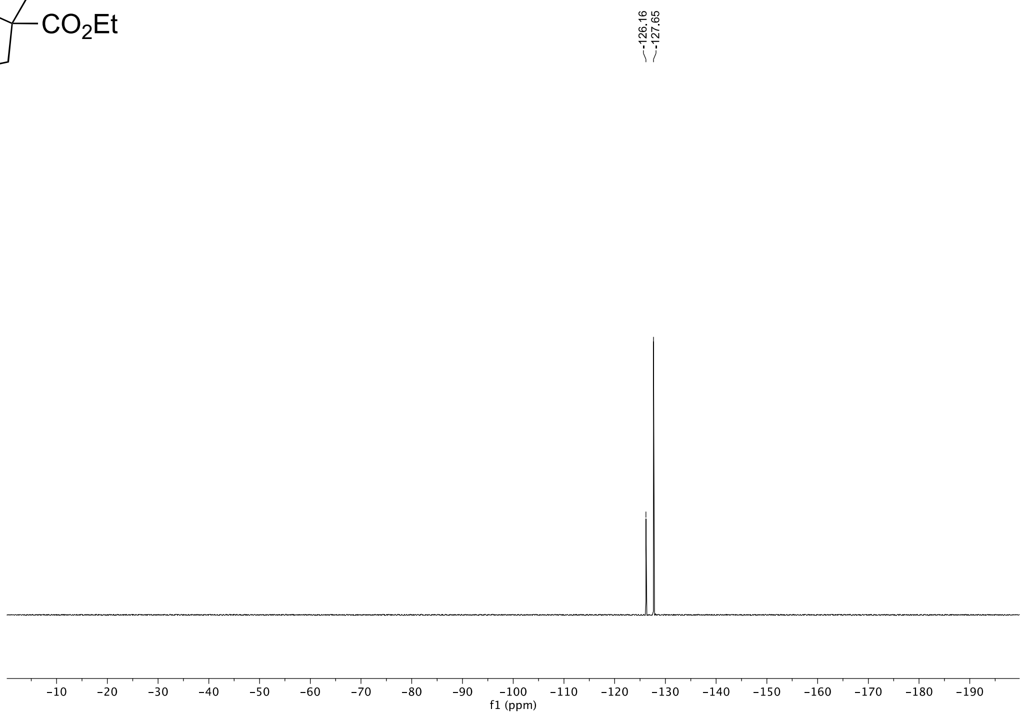

Ethyl-3-(3-bromophenyl)-4-(1-(2-(*tert*-butoxycarbonyl)hydrazineylidene)ethyl)-1-phenylimidazolidine-4-carboxylate (3Fa)

^{1}H NMR (400 MHz, DMSO-d_6) of **3Fa:**

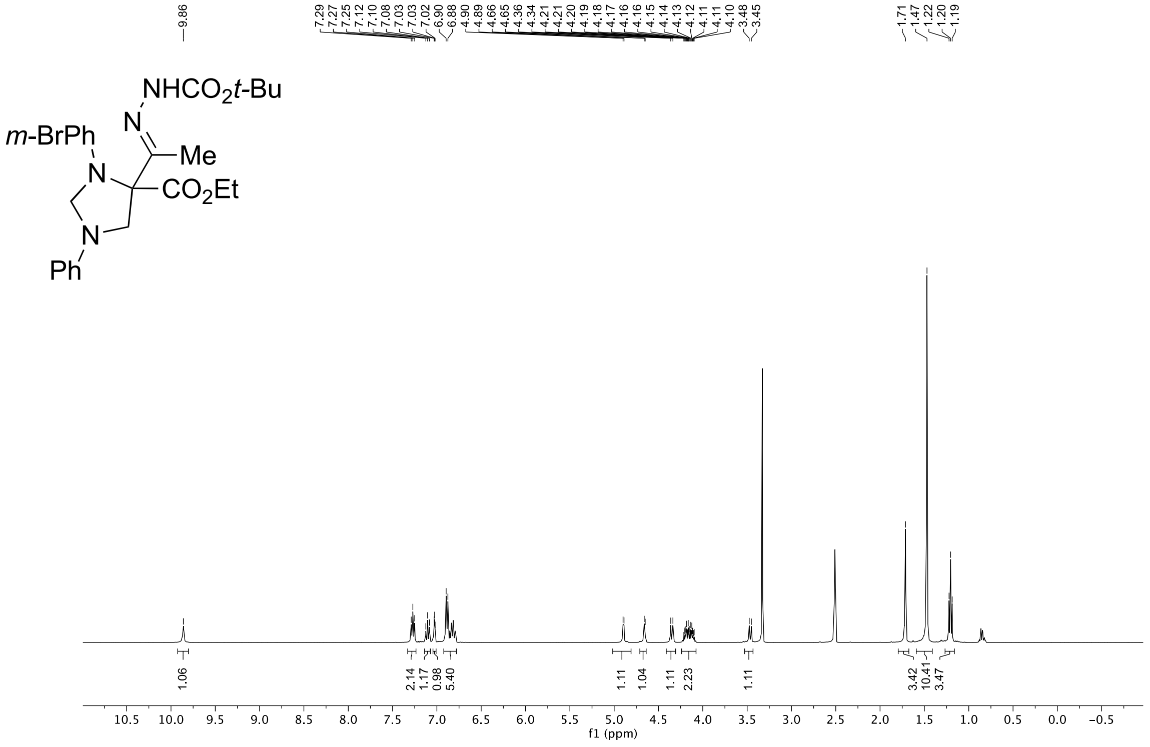

^{13}C {^{1}H} NMR (101 MHz, DMSO-d_6) of **3Fa:**

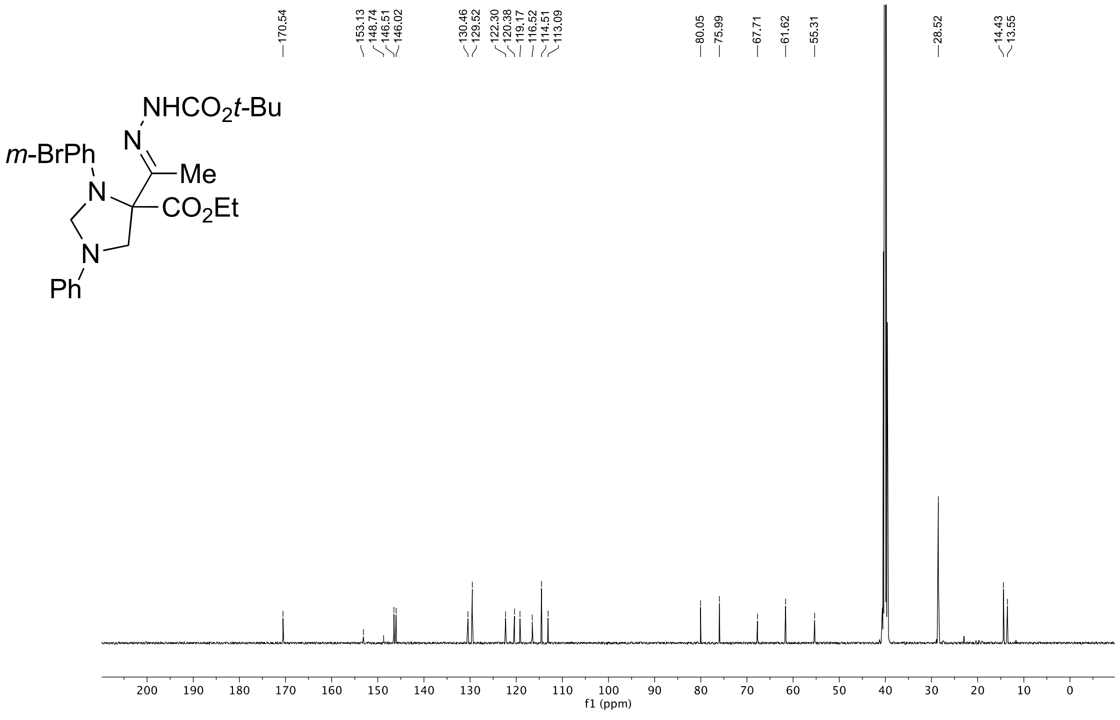

Ethyl 4-acetyl-1,3-diphenylimidazolidine-4-carboxylate (4a)

^{1}H NMR (400 MHz, DMSO-d_6) of **4a:**

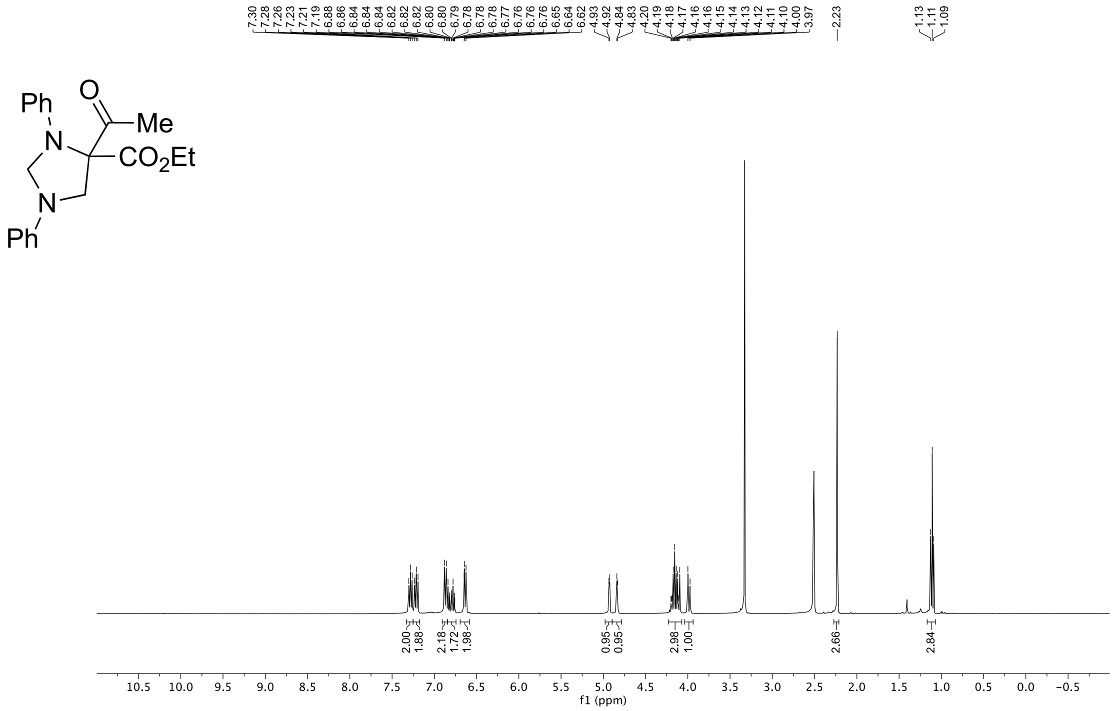

^{13}C {^{1}H} NMR (101 MHz, DMSO-d_6) of **4a:**

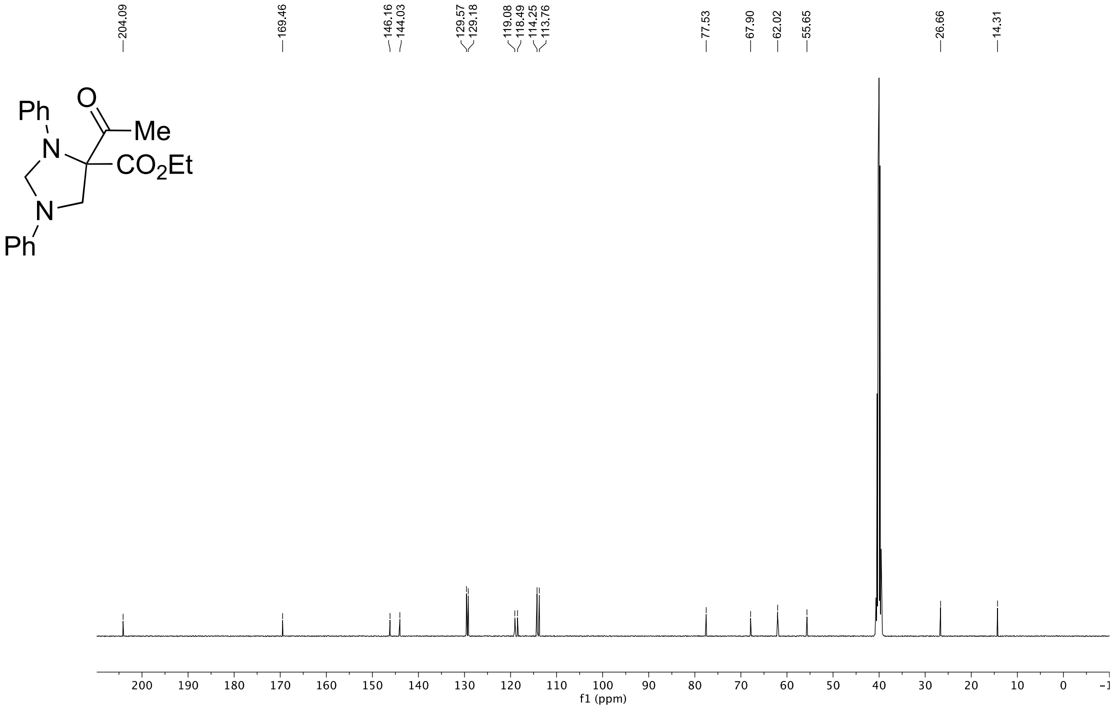

Supplement: Supplementary file 1 [file jo5c01387_si_001.pdf]
